# Detection of Genome‐Wide IGF‐1R Recruitment to Enhancer and Promoter Regions of Chromatin in Clinical Prostate Cancers

**DOI:** 10.1002/cam4.71257

**Published:** 2025-09-30

**Authors:** Jack V. Mills, Avigail Taylor, Reema Singh, Jinseon Kim, Simon Engledow, Richard Colling, Clare Verrill, Ian G. Mills, Valentine M. Macaulay

**Affiliations:** ^1^ Nuffield Department of Surgical Sciences University of Oxford Oxford UK; ^2^ Wellcome Centre for Human Genetics University of Oxford Oxford UK; ^3^ Department of Cellular Pathology Oxford University Hospitals NHS Foundation Trust Oxford UK; ^4^ Oxford NIHR Biomedical Research Centre Oxford United Kingdom

**Keywords:** ChIP‐seq, enhancer, IGF‐1R, promoter, prostate cancer

## Abstract

**Introduction:**

Nuclear insulin‐like growth factor‐1 receptor (IGF‐1R) undergoes IGF‐induced recruitment to cancer cell chromatin in vitro and associates with advanced prostate cancer (PCa) stage in clinical tissue, prompting this investigation of IGF‐1R chromatin recruitment in vivo.

**Methods:**

Human tissues surplus to diagnostic need were obtained from consenting patients undergoing transurethral resection of the prostate (TURP) or radical prostatectomy (RP). Initial tissue samples were processed for H3K4me1‐positive control ChIP to optimise homogenisation, fixation and ChIP conditions. Following successful method optimization, IGF‐1R and H3K4me1 ChIP‐seq was performed on six treatment‐naïve localized PCa samples, along with parallel IGF‐1R immunohistochemistry analysis. MACS2 and LanceOtron peak callers were used to identify binding sites from ChIP‐seq data and MEME Suite was used to identify an IGF‐1R binding motif. In vitro chromatin immunoprecipitation qPCR (ChIP‐qPCR) was used for ChIP‐seq data validation.

**Results:**

We identified 5743 unique IGF‐1R binding sites, with 37% within 3 kb of gene transcription start sites (TSSs). Of these sites, 72.3% coincided with enhancer mark H3K4me1, suggesting regulatory function. Motif analysis identified an IGF‐1R consensus binding motif for the first time, with a sequence resembling that of the insulin receptor and PITX2 transcription factor binding motifs, supporting functional similarities. In vitro ChIP‐qPCR confirmed IGF‐1R recruitment to a site identified in vivo in the *RRM2* TSS, a gene involved in DNA replication and repair and regulated by the IGF‐axis, highlighting potential regulatory function of nuclear IGF‐1R.

**Conclusion:**

Overall, these data represent the first characterization of genome‐wide IGF‐1R recruitment in PCa tissue and are consistent with a transcriptional regulatory role, further elucidating the contribution of nuclear IGF‐1R to advanced clinical stage.

**Abbreviations**
AR: androgen receptor
ARBS: androgen receptor binding sites
ChIP‐qPCR: chromatin immunoprecipitation qPCR
ChIP‐seq: chromatin immunoprecipitation sequencing
CRPC: castrate‐resistant prostate cancer
DSB: double strand break
FFPE: formalin fixed paraffin embedded
GO: gene ontology
HR: homologous recombination
IGF‐1R: insulin‐like growth factor‐1 receptor
IHC: immunohistochemistry
INSR: insulin receptor
IR: ionising radiation
KEGG: Kyoto Encyclopaedia of Genes and Genomes
NHEJ: non‐homologous end joining
PANTHER: protein analysis through evolutionary relationships
PCa: prostate cancer
RNR: ribonucleotide reductase
RP: radical prostatectomy
RTK: receptor tyrosine kinase
TSS: transcription start site
TURP: transurethral resection of the prostate
WCE: whole cell extract

## 1 Introduction

Type 1 insulin‐like growth factor receptor (IGF‐1R) is a transmembrane RTK, the main mediator of IGF signalling and is closely related to the insulin receptor (INSR) [1, 2]. IGF signalling is frequently deregulated in cancers and contributes to cancer growth and metastasis by promoting cell survival, proliferation, and invasion [3].

The androgen receptor (AR) signalling pathway is the principal driver of prostate cancer (PCa) development. AR is a nuclear hormone receptor that, upon ligand binding and nuclear translocation, is recruited to androgen receptor binding sites (ARBS) to drive a pro‐tumorigenic transcriptional programme [4]. Treatment of locally advanced or metastatic PCa involves androgen deprivation therapy, but castrate‐resistant PCa (CRPC) inevitably arises. CRPC can be driven by AR‐independent mechanisms or sustained androgen‐independent AR signalling via AR variants or receptor tyrosine kinase (RTK) cross‐talk, including via IGF‐1R [5, 6, 7]. There is evidence that androgens upregulate IGF‐1R, while IGF‐PI3K activation can promote ligand‐independent activation of AR, including AR splice variants [8, 9]. Recognition of these functions led to the emergence of the IGF axis as a target for therapeutic intervention. However, despite some cases showing exceptional responses [10, 11], the majority of IGF/IGF‐1R inhibitor trials failed due to factors including lack of predictive biomarkers, dose‐limiting hyperglycaemia resulting from INSR co‐inhibition, and incomplete understanding of IGF‐1R biology [12].

In addition to its role in canonical signalling from the cell surface, we and others have detected IGF‐1R in the nucleus [13, 14]. We reported that nuclear IGF‐1R is more frequently detected in malignant vs. benign epithelium and associates with advanced PCa stage and reduced overall survival in renal cancer [14, 15]. There is also evidence that nuclear IGF‐1R associates with response to IGF‐1R antibody therapy in patients with sarcoma [16], suggesting that nuclear IGF‐1R indicates IGF dependence and could be a potential biomarker for response to IGF‐axis targeting treatments. Performing IGF‐1R chromatin immunoprecipitation‐sequencing (ChIP‐seq) in cultured human melanoma cells, Larsson and colleagues identified 568 mostly intergenic IGF‐1R binding regions, with several shown to mediate transcriptional activation [13]. More recently, we reported IGF‐induced IGF‐1R recruitment to chromatin of PCa and Ewing sarcoma cells [15]. In contrast to findings of the work by the Larsson group [13], we identified < 70 unique sites, clustering at or near transcription start sites (TSSs) of genes including *JUN* and *FAM21A*. Of these sites, 95% were coincident with RNA Polymerase II (RNAPolII) recruitment and 87% with H3K4me1 and 50% with H3K4me3, markers of active enhancers and promoters respectively [17]. We found that IGF‐1 treatment of PCa cells induced interaction between IGF‐1R and RNAPolII, promoted recruitment of both IGF‐1R and RNAPolII to the *JUN* and *FAM21A* promoters, and upregulated the expression of these genes [15]. Nuclear IGF‐1R has also been shown to interact with other nuclear proteins including transcription factors to alter their function [18, 19, 20, 21].

Overall, given the identified function of IGF‐1R recruitment to chromatin in vitro and the association of nuclear IGF‐1R with advanced clinical cancer phenotypes and response to IGF‐axis targeting therapy, we therefore wanted to investigate the functional contribution of IGF‐1R recruitment to chromatin in vivo. To investigate this, we here performed IGF‐1R ChIP‐seq from clinical samples of prostate tissue for the first time.

## 2 Materials and Methods

### 2.1 Cell Lines

DU145 PCa cells (Cancer Research UK Clare Hall Laboratories, RRID: CVCL_0105) were cultured in RPMI 1640 medium with 10% fetal calf serum (FCS). Cells were mycoplasma‐free when tested with MycoAlert (Lonza Rockland Inc.) and were authenticated by STR genotyping (Eurofins Medigenomix Forensik GmbH). Early passage stocks were expanded and cryopreserved and used within 20 passages of recovery.

### 2.2 Prostate Tissues

Human tissues surplus to diagnostic need were obtained at the Churchill Hospital, Oxford, UK from consenting patients undergoing transurethral resection of the prostate (TURP) or radical prostatectomy (RP), the latter sampled from benign and cancer areas as described in [22]. Samples were collected and used with the approval of the South Central–Oxford C Research Ethics Committee (REC reference 07/H0606/120) and Oxford Radcliffe Biobank (ORB_20/A032). Fresh tissues were snap frozen and stored at −80°C, and the remaining tissue was processed by formalin fixation and paraffin embedding (FFPE) for histopathological analysis. Unfixed FFPE sections for immunohistochemistry (IHC) were obtained via the Oxford Centre for Histopathology Research (OCHRe). Relevant clinical data were pseudo‐anonymised and stored in password‐protected files.

### 2.3 Western Blotting and IHC

Whole cell extracts (WCE) and chromatin were analysed by western blotting as described in [15] using antibodies to IGF‐1R (#3027, Cell Signalling Technology, Leiden, The Netherlands) and Histone H3 (ab1791, Abcam, Cambridge UK). IHC was performed on freshly cut 4 μm FFPE tissue sections using antibody to IGF‐1R (#9750, Cell Signalling Technology) as in [23] and phospho‐Y1161 IGF‐1R (orb644426, Biorbyt Ltd., Cambridge UK) on a Leica‐Bond autostainer with antigen retrieval in Tris‐EDTA pH 9. IHC signal was semi‐quantified by scoring total and phospho‐IGF‐1R in the membranes, cytoplasm, and nucleus for intensity (0, nil; 1, weak; 2, moderate; 3, heavy) and percentage (0%, nil; 1: 1%–10%; 2: 11%–50%; 3: 51%–80%; 4: 81%–100%) of tumour stained, generating immunoreactive scores (range 0–12) for membrane, cytoplasmic, and nuclear total and phospho‐IGF‐1R as [15].

### 2.4 Chromatin Immunoprecipitation (ChIP) and Quantitative PCR (qPCR)

Cultured cells were fixed with 1% formaldehyde, DNA was fragmented by sonication (Bioruptor Pico sonicator, Diagenode Cat. No. B01060010, 15 cycles, 30 s on/off), and processed for ChIP using the Merck Millipore ChIP kit (Cat. No. 17‐295) as per the manufacturer's protocol with minor modifications as in [15].

Fresh frozen TURP chippings were cut into 1–2 mm^3^ sections, fixed in 1% formaldehyde for 10 min, quenched with 125 mM glycine for 5 min and homogenised and lysed in 500 μL SDS Lysis buffer containing protease inhibitor in Lysing Matrix A ceramic bead tubes (MP biomedical Cat. No. 6910050) using a FastPrep Homogeniser. Lysates were sonicated and processed for ChIP as described above.

Frozen RP punch biopsies were processed as described by Singh et al. [24] with minor modification, cutting tissue into 30 μm slices on a Leica CM1950 cryostat, fixing in 1% formaldehyde and quenching with 125 mM glycine. Tissues were lysed in 500 μL SDS Lysis buffer, homogenised using a Pellet Pestle homogeniser (Sigma‐Aldrich) and sonicated and processed for ChIP as described above.

Further details of optimisation are provided in Data S1: Section 1.1.

Antibodies used for ChIP were IGF‐1R (CST‐3027) and H3K4me1 (Abcam, ab‐8895). Normal Rabbit IgG (CST‐2729) or beads only (salmon sperm DNA/Protein A agarose slurry) were used as controls.

The qPCR used primers to amplify IGF‐1R binding regions of the *JUN* and *FAM21A* promoters [15], or *RRM2* TSS (Forward Primer 5′‐TTGGAAGTCGCGCTAACCTT‐3′, Reverse Primer 5′‐ACTCCACCATTGGTCTGCAC‐3′).

### 2.5 ChIP‐Seq Peak Calling Analysis

ChIP DNAs from RP biopsies were sequenced at the Oxford Genomics Centre, University of Oxford, at 40–60 million read depth. ChIP‐Seq reads were mapped using Bowtie2 [25] and aligned to the human reference genome from UCSC, using hg19 for comparison with previously obtained data from DU145 PCa cells [15]. Quality control measures are as described in Data S1: Section 1.2 and shown in Figure S1 and Table S1.

For all peak‐calling algorithms used (MACS2, MACS2‐broad, and LanceOtron), BEDTools multiple intersect [26] with default settings was used to combine peaks from each algorithm into a single peak and define overlap in peak sets called by the individual algorithms. IGF‐1R and H3K4me1 peak sets were filtered against the control peak set to remove non‐specific/background peaks.

BEDTools merge was used to combine the three sets of merged IGF‐1R peaks identified using MACS2, MACS2‐broad, and LanceOtron [27, 28]. This process was repeated for H3K4me1, and the BEDTools intersect sub‐command was employed to identify IGF‐1R binding peaks coincident with H3K4me1 binding peaks (peaks overlapping by 1 base pair or more).

ChIPseeker was used to annotate IGF‐1R binding peaks with nearby genes and calculate distances to their TSSs [29, 30]. We defined a ‘union’ set of genes as those with a TSS proximal to an IGF‐1R binding peak called by any algorithm in at least one RP, and ‘consensus’ genes with a TSS proximal to an IGF‐1R binding peak called by any algorithm in at least two RPs. Using the same method, we annotated H3K4me1 binding peaks with nearby genes and distances to corresponding TSSs.

The same approaches (Data S1: Section 1.3) were used to reanalyse the ChIP‐seq data we had previously reported in DU145 cells using only the MACS2 ‘narrow’ algorithm [15]. The resulting peak sets called by all 3 peak callers in DU145 were used to assess overlap with peaks called in RP tissues (Data S1: Section 1.4).

To assess coincidence of IGF‐1R binding peaks and ARBS, we accessed AR ChIP‐seq from two cases of treatment‐naïve primary PCa [31] from the GEO accession viewer (GSM698575 and GSM698756; https://www.ncbi.nlm.nih.gov/geo/query/acc.cgi?acc=GSE28219). After using liftOver (https://genome.sph.umich.edu/wiki/LiftOver) to re‐map AR peaks from hg18 to hg19, GSM698575 and GSM698576 had 8559 and 484 peaks, respectively. Both peak sets were combined using BEDTools merge with default settings, resulting in 8777 non‐overlapping ARBS. BEDTools intersect was used with default settings to identify IGF‐1R binding peaks coincident with ARBS. Peaks were visualised using the UCSC genome browser.

### 2.6 IGF‐1R Binding Motif Analysis

From the identified peaks across all samples and all peak calling algorithms, all 5743 unique regions were used as the input sequences for the IGF‐1R binding motif analysis. The MEME Suite 5.5.4 software tools package (https://meme‐suite.org/meme/index.html) was used for this analysis, specifically the XTREME motif discovery programme for comprehensive motif analysis and discovery [32]. Shuffled input sequences were used as control inputs; Tomtom was used with default settings to match discovered motifs to the human Hocomoco v11 database. Statistically significant motifs were those with *E*‐value ≤ 0.05, and the software parameters were set to identify motifs 6–15 bases long, as per standard settings.

### 2.7 Biological Pathway Analysis

The ‘consensus’ set of genes (104 genes with a TSS proximal to an IGF1R peak in ≥ 2 samples) was used as input for PANTHER (Protein ANalysis THrough Evolutionary Relationships) Classification, Gene Ontology (GO), and Kyoto Encyclopaedia of Genes and Genomes (KEGG) over‐representation analyses. These analyses were performed using standard settings as per the online browser tools, with *Homo sapiens* selected as the reference gene list and enrichment defined by FDR and *p*‐value < 0.05.

## 3 Results and Discussion

### 3.1 Optimizing IGF‐1R ChIP for Prostate Cancer Tissue

Previously, we identified the *JUN* and *FAM21A* promoters as IGF‐1R binding sites in cultured PCa cells and were able to detect nuclear IGF‐1R recruitment to these sites in 5 of 6 fresh frozen RP biopsies [15]. Anticipating that ChIP‐seq would require larger amounts of tissue, initial experiments tested IGF‐1R recruitment by ChIP‐qPCR using fresh TURP chippings (0.7–2.0 g) from three CRPC patients. We successfully detected IGF‐1R recruitment to the *JUN* and *FAM21A* promoters in all TURPs (Figure S2A). We therefore intended to use similar amounts of fresh RP tissue, but this would have risked compromising diagnostic assessment of tumour margins. Therefore, we used 4 mm^3^ RP punch biopsies (0.1–0.3 g) from six RPs, sampled as in [22] from malignant tissue (including two separate cancer areas in RP2) and macroscopically benign areas.

Review of H&E‐stained sections around the biopsy sites confirmed that all ‘benign’ biopsies were surrounded by benign tissue. ‘Cancer’ biopsies were surrounded by malignant epithelium, with a mixture of benign and malignant epithelium abutting the biopsy site of RP5 (Table 1). Chromatin was extracted from RP biopsies, and detection of IGF‐1R and Histone H3 by western blot confirmed their presence for detection in ChIP (Figure S2B). We previously detected enhancer mark H3K4me1 at the *JUN* and *FAM21A* promoters in PCa cells [15], so we used this as a positive control to confirm successful ChIP from tissue here. Successful detection of H3K4me1 enrichment by ChIP‐qPCR (Figure S2C–E) suggested that the protocol performed acceptably using small amounts of input tissue material, so samples were therefore processed for IGF‐1R and H3K4me1 ChIP‐seq.

**TABLE 1:** Characteristics of fresh frozen RP biopsies collected for ChIP and ChIP‐seq.

RP no.	Path	GG	pT	IGF‐1R IHC score M/C/N		ChIP‐seq peaks (n)					
						H3K4‐me1			IGF‐1R		
				Total	Phospho	MACS2		Lance‐Otron	MACS2		Lance‐Otron
						N	B		N	B	
1	M	2	2	12/8/0	0/4/9	ND	ND	ND		ND	ND
2	M_1	2	2	6/4/0	0/4/12	ND	ND	ND	42	118	13
	M_2					ND	ND	ND	144	225	41
3	M	2	3a	12/4/0	0/1/9	45,385	86,660	24,550	188	269	14
4	M	2	2	12/8/0	1/4/6	ND	ND	ND	46	179	37
5	B/M	2	2	12/12/0	2/6/12	68,794	104,027	31,924	230	878	65
6	M	2	3a	12/9/2	3/4/9	40,483	75,206	16,718	23	52	17

*Note:* Sample number was assigned in order of tissue collection. Table shows pathology (Path) adjacent to the biopsy punch used for ChIP‐seq (M, malignant; B, benign), grade group (GG), pathological stage (pT), total and phosph‐IGF‐1R IHC score (M, membrane; C; cytoplasm; N, nuclear) and number of peaks of H3K4me1 and IGF‐1R enrichment called by MACS2 narrow (N) and broad (B) parameters and LanceOtron. RP1 was used during initial optimisation of ChIP protocol. Both RP2 samples were malignant biopsies taken from the same RP case. The RP5 punch was surrounded by admixed benign and malignant tissue.

**FIGURE 1:**
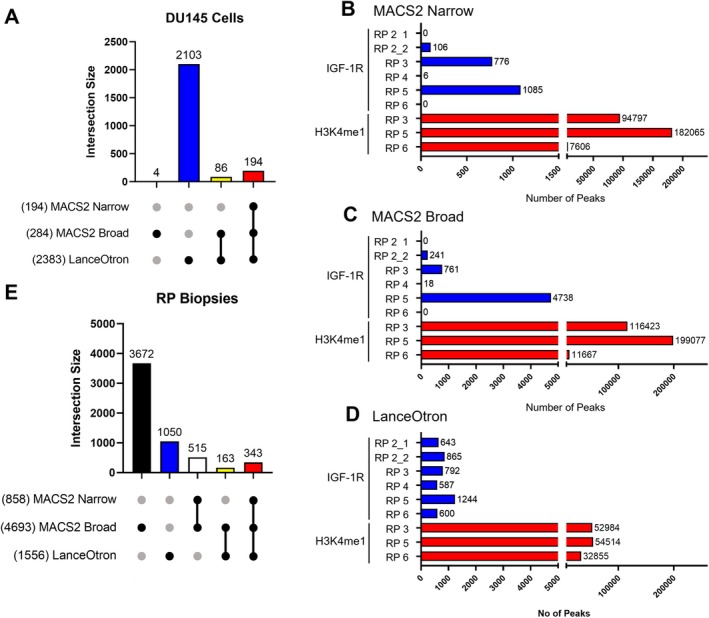
Overlap of ChIP‐seq peaks called by three different peak calling programs in cultured prostate cancer cells and clinical pcrostate cancers. (A) Peaks of IGF‐1R recruitment were called in DU145 prostate cancer ChIP‐seq data using MACS2, MACS2‐broad and LanceOtron. Peaks from the three programs were then merged such that overlapping and “book‐ended” peaks were combined into a single peak spanning all of the combined peaks (see Section 2.5). The UpSet plot shows the total number of combined peaks with at least one contributing peak from each of the individual calling methods (x axis label), and the total number of combined peaks comprised of each combination of contributing calling methods (vertical bar chart). (B–D) Bar charts showing the number of IGF‐1R and H3K4me1 peaks identified in each indicated RP biopsy sample using either MACS2‐Narrow (B) MACS2‐Broad (C) or LanceOtron (D) peak calling software. (E) As for (A) but depicting peaks of IGF‐1R recruitment found in six RP biopsies.

In parallel, we performed IHC for total and phosphorylated IGF‐1R on FFPE RP sections, focusing on the signal around punch biopsy sites. There was more IGF‐1R staining in malignant versus benign glands, consistent with previous work [33]. In all samples, 90% of cells were positive for membrane IGF‐1R and 80% positive for cytoplasmic IGF‐1R. Only one sample contained detectable nuclear IGF‐1R, but all had nuclear phospho‐Y1135/1136 IGF‐1R (Table 1, Figure S3). These results highlight discrepancies between nuclear IGF‐1R detection methods and contrast with our previous report of nuclear IGF‐1R positivity in ~50% of a larger set (*n* = 137) of RPs [15]. All contained nuclear phospho‐IGF‐1R signal, but this may reflect the cross‐reactivity of the phospho‐IGF‐1R antibody with phospho‐INSR, which also undergoes nuclear translocation [34]. However, the IGF‐1R antibody used for IHC does not cross‐react with INSR, therefore likely explaining the higher abundance of signal in phospho‐IGF‐1R detection. The IGF‐1R antibody used for ChIP‐seq also does not cross react with INSR, and given that we were subsequently able to detect evidence of genome‐wide IGF‐1R recruitment using ChIP‐seq (Figure 1), we speculate that IHC has a higher threshold for nuclear IGF‐1R detection than ChIP‐seq.

Furthermore, due to time constraints, FFPE sections were analysed by IHC only after fresh tissues underwent ChIP‐seq. In the future, it will be informative to analyse a larger cohort of RPs representing a range of tumour grades and stages to identify samples with IHC‐detectable nuclear IGF‐1R for analysis by ChIP‐seq. Variable detection of IGF‐1R recruitment may also reflect differing levels of nuclear IGF‐1R, which could have been assessed had nuclear IGF‐1R been detectable by IHC (Figure S3).

### 3.2 IGF‐1R Is Recruited to Chromatin Across the Genome of Clinical PCa

We performed ChIP‐seq on 16 RP‐derived punch biopsy samples: one input (total chromatin) control from RP3, six samples from three malignant biopsies (RPs 2_1, 2_2, 4) processed for control and IGF‐1R ChIP, and nine samples from three biopsies (RPs 3, 5, 6) processed for control, H3K4me1, and IGF‐1R ChIP. For peak calling, we used MACS2, a standard ChIP‐seq peak calling programme commonly used to detect narrow peaks of enrichment typical of authentic transcription factors. However, MACS2 can may fail to call atypically shaped regions of enrichment, including those that can be identified visually as enriched peaks in a genome browser [28, 35]. Therefore, we also used LanceOtron, a new deep‐learning peak caller that incorporates a visualisation tool to optimise peak calling (e.g., by peak shape) according to features of specific datasets rather than by fixed criteria as in MACS2 [27]. LanceOtron was shown to outperform MACS2 in different data types, including ChIP‐seq, and features that were uniquely identified by LanceOtron were found to be enriched for enhancers or promoters [27].

In our previous analysis [15], peaks of IGF‐1R recruitment had been called using MACS2 for only narrow peaks, as appropriate for the analysis of transcription factor binding. Based on this, it may then have been predictable that identified regions of IGF‐1R recruitment would be narrow peaks near to TSSs. However, we reasoned that the shape of IGF‐1R enrichment sites may not necessarily conform to patterns exhibited by canonical transcription factors. Therefore, we first re‐analysed DU145 cell line data we had previously interrogated, using MACS2 with ‘narrow’ peak calling arguments (‘MACS2‐narrow’), MACS2 with ‘broad’ peak calling (‘MACS2‐broad’) and LanceOtron. This analysis identified 2387 unique IGF‐1R peak regions in DU145 (Figure 1A, Table S3). While data generated by LanceOtron would require ChIP‐qPCR validation, this analysis suggests we may previously have failed to detect some IGF‐1R binding regions in PCa cells, where our original analysis using only MACS2‐narrow identified 62 regions of IGF‐1R enrichment [15].

We therefore used all 3 peak callers to analyse the ChIP‐seq from clinical prostate cancers. Consistent with our previous cell line data [15] and other ChIP‐seq studies in clinical tissues [24, 31, 34], initial peak calling identified ~7000–200,000 sites of H3K4me1 enrichment and ~0–1300 sites of IGF‐1R enrichment across all peak calling programs, suggesting the ChIP‐seq protocol had worked successfully (Table 1, Figure 1B–D). When we combined IGF‐1R binding peaks called in any RP biopsy by any peak‐calling algorithm, we identified 5743 non‐overlapping, unique IGF‐1R peak regions. Of these unique regions, 858 spanned at least one peak called by MACS2‐narrow; all of these were spanned by a MACS2‐broad peak, and 343/858 (40%) were also spanned by a LanceOtron peak and were therefore called by all three algorithms (Figure 1E). In contrast to cell line data, only 1556/5743 (27%) unique peak regions spanned a LanceOtron peak, of which only 506/1556 (33%) also spanned a MACS2‐narrow or MACS2‐broad peak. In light of the exclusion by LanceOtron of a large number of IGF‐1R peaks identified in tissues by MACS2 (Figure 1E), further experimental validation and optimisation of LanceOtron peak score cut‐off need to be carried out, together with the LanceOtron peak visualisation tool to guide identification of authentic IGF‐1R binding sites. The visualisation tool within LanceOtron allows optimisation of peak calling for features of specific datasets, i.e., peak shape, rather than using fixed criteria as with MACS2 [27, 28]. Therefore, further optimisation of LanceOtron will likely have identified peaks that may be excluded by MACS2. However, the novelty of LanceOtron means that it currently lacks robust experimental validation by its developers [27].

Overall, this data showed detection of IGF‐1R recruitment to chromatin binding sites across the genome in vivo for the first time.

### 3.3 Sites of IGF‐1R Recruitment in Clinical Cancers Are Distinct From Sites in Cultured Cells and ARBS

**FIGURE 2:**
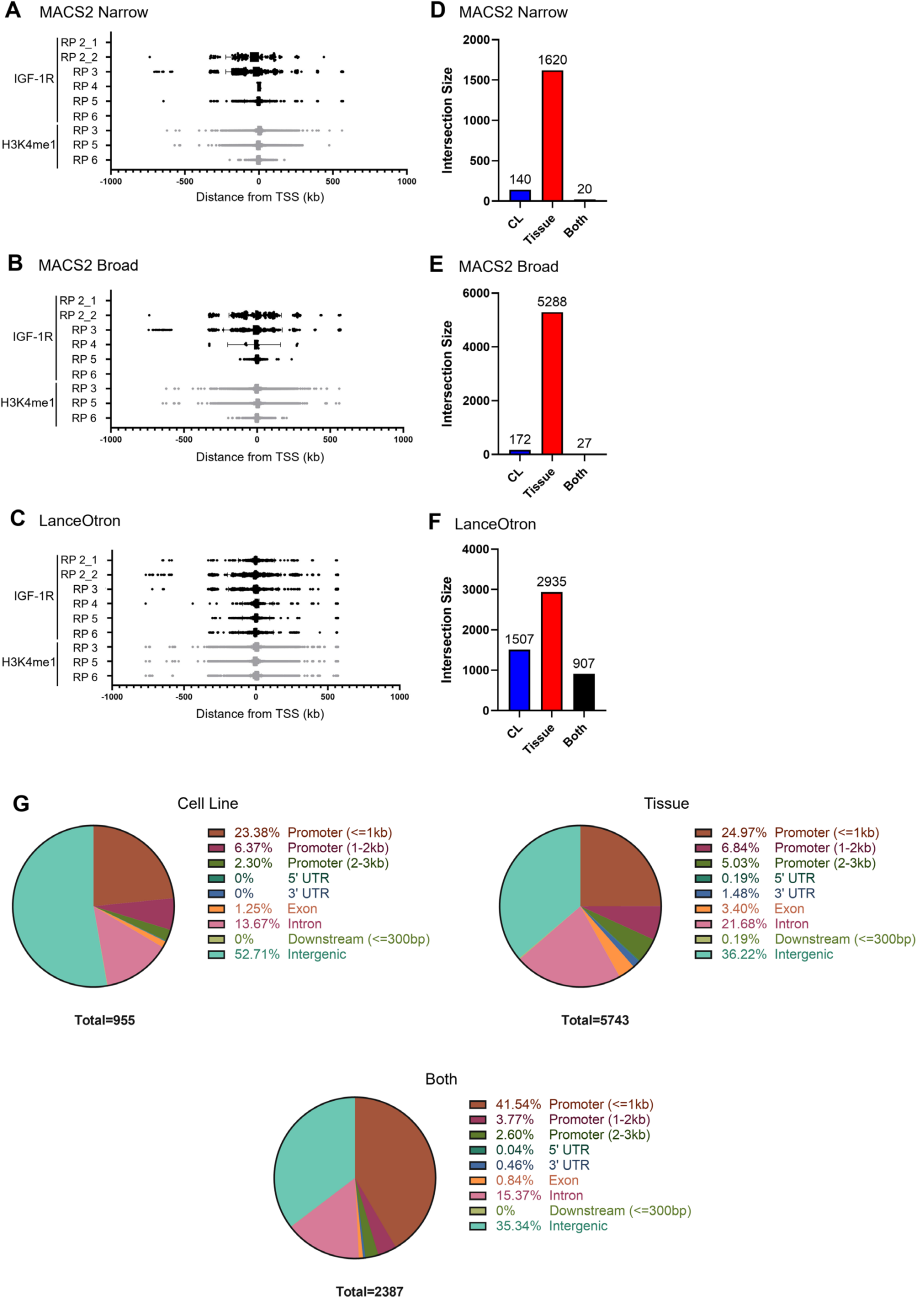
Detection of IGF‐1R recruitment across the genome. (A–C) Graphs show boxplot of distribution of distance (kb) of peak from TSS per sample called by MACS2‐narrow (A) MACS2‐broad (B) or LanceOtron (C). (D–F) For each of MACS2‐narrow (D), MACS2‐broad (E), and LanceOtron (F), peaks called in DU145 cells and in RP biopsies were merged and bar charts were plotted showing the number of these peaks that were identified in DU145 cells only (CL), Tissue samples only (Tissue) or both (Both). Note that we do not expect the total number of combined peaks to necessarily tally between the UpSet plots shown in Figure 1A,B because the way in which peaks are merged depends on the sets of peaks being combined. (G) Summary of peak distribution relative to gene features as determined by ChIPseeker for IGF‐1R peaks identified by any algorithm in DU145 cells (Cell line), the tissue samples processed in this work (Tissue) and the peaks identified in both (Both).

Next, we assessed the location of the 5743 sites of IGF‐1R recruitment, identifying a pattern of clustering around TSSs (Figure 2A–C). Furthermore, 4155 of the 5743 combined peak regions (72.3%) were coincident with peaks of enrichment of the enhancer mark H3K4me1. This is consistent with the pattern of sites of IGF‐1R recruitment we reported in cultured cells [15] and suggests these sites may have a regulatory function.

To assess the coincidence of sites of IGF‐1R recruitment in PCa cells and clinical cancers, we conducted a within‐algorithm comparison of IGF‐1R peaks called in RPs versus cultured cells (see Section 2.5 and Data S1: Section 1.4). Only 20 (1.1%) MACS2‐narrow, 27 (0.5%) MACS2‐broad and 907 (17%) LanceOtron peaks were detected in both DU145 cells and clinical cancers (Figure 2D–F and Table 4). This suggests only a minority of IGF‐1R recruitment sites in tissues were detectable in cultured cells. The reasons for this are unclear, but the fact that DU145 cells are AR‐negative, do not secrete PSA, and are derived from a brain metastasis of a prostate adenocarcinoma may play a role. Cellular heterogeneity within the tissue samples and differences in activation pathways, which are not necessarily IGF1‐dependent, may also likely play a role [31]. Despite multiple attempts, using methods as for DU145 cells and as described in [31], we were unable to optimise DNA fragmentation for IGF‐1R ChIP‐seq in AR‐positive 22Rv1 PCa cells to assess this (data not shown). Overall, across all 3 algorithms, peaks identified in cell lines were most commonly found in intergenic regions (~53%) followed by promoter regions (~32%). However, in tissues, peaks were most commonly found within promoter regions (~37%), and of those peaks identified in both cell lines and tissues, almost half were found within promoter regions (~48%) (Figure 2G). Identified peaks most commonly being found within promoter regions suggests a functional role for nuclear IGF‐1R recruitment to chromatin in vivo.

Given the importance of crosstalk between the IGF‐axis and AR signalling [36, 37, 38], as mentioned previously, we investigated the overlap between ARBS and the 5743 unique IGF‐1R binding sites in RP biopsies. Notably, a previous study by Sharma et al. of AR recruitment to chromatin showed over 1000 highly conserved, tissue‐specific chromatin binding sites for AR detected in CRPC clinical tissue that were not detected in cell lines [31], potentially due to the more heterogeneous nature of clinical tissue compared to immortalised cell lines. To assess any overlap and potential crosstalk of these ARBs with the IGF‐1R binding sites identified in RP biopsies in this work, we accessed the publicly available AR ChIP‐seq data from this previous study from two prostate cancers obtained from treatment naïve patients [31]. The previous study identified 8559 and 484 ARBS in the two cases, which we merged to obtain 8777 non‐overlapping ARBS. Only 7.1% (407/5743) of IGF‐1R peaks overlapped with ARBS (Table S5). Thus, we find that IGF‐1R is recruited to a small set of chromatin regions in clinical cancers that are mostly distinct from ARBS. This suggests that the contribution of the IGF‐axis to androgen signalling is unlikely to be via IGF‐1R recruitment to ARBS, at least in the localised, treatment‐naïve cases we studied here. CRPC can arise from sustained ligand‐independent AR signalling or AR indifferent mechanisms [5, 7]. To explore the possibility that nuclear IGF‐1R contributes to androgen independent AR recruitment, it will be informative in future to include CRPC samples, to investigate whether IGF‐1R is recruited to a greater proportion of ARBS in cancers driven by androgen independent AR activation.

### 3.4 Proximity of IGF‐1R Binding to TSSs of Genes Encoding Proteins That Regulate Translation and Scaffold/Adaptor Function

**FIGURE 3:**
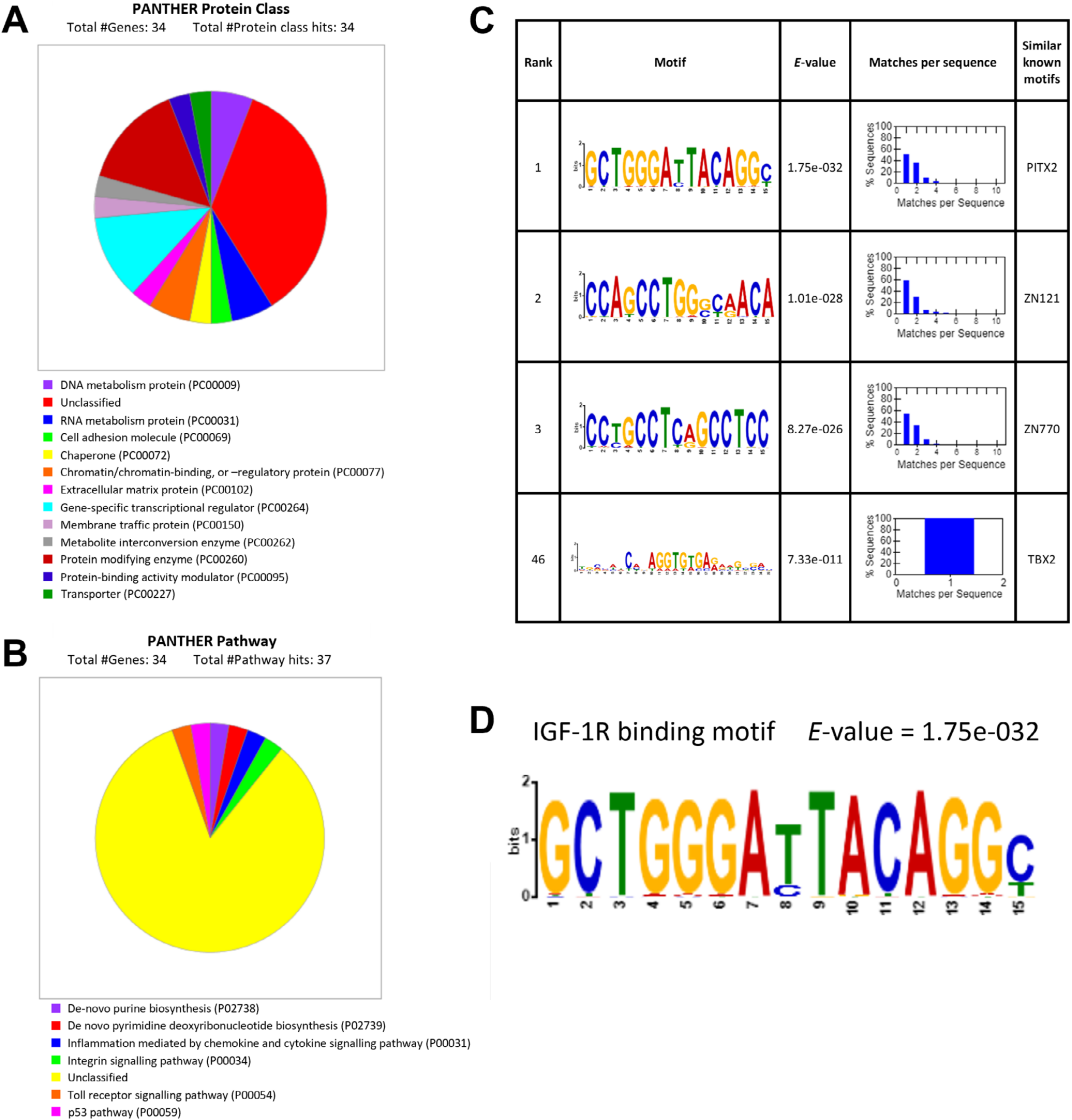
Biological pathway analysis and motif analysis of IGF‐1R binding sites identified in vivo. (A, B) PANTHER analysis showing the protein class (A) and biological pathway processes (B) of consensus genes identified as having IGF‐1R peaks proximal to their TSS in 2 or more of the tissues processed for ChIP‐seq. (C) Motif analysis using the MEME‐suite XSTREME algorithm of 5743 unique IGF‐1R binding sites in prostate tissue. Table shows the top 3 ranked motifs that were significantly enriched. Also worth noting is the 46th ranked motif, the only motif to be identified in all IGF‐1R binding site sequences. (D) The top ranked IGF‐1R DNA binding motif identified using motif discovery on the 5743 unique IGF‐1R binding sites identified here using ChIP‐seq. This binding motif showed similarity with the INSR‐β binding motif previously identified by [34], with both containing a CTGGGA motif.

We used the original 5743 IGF‐1R binding peaks, called in any RP biopsy by any of the three peak‐calling algorithms, to identify a ‘union’ gene set comprising 1875 genes with a TSS proximal to a peak in at least one RP sample, and a ‘consensus’ set of genes comprising 104 genes with a TSS proximal to a peak in at least two or more RP samples (Table S6). Of the ‘consensus’ genes identified, only nine were detected in the majority of RPs (4 or more), and only 1 was detected in all RP samples, the *ROCK1P1* pseudogene (Table S7), which has been shown to play a potential role in mediating PCa migration and invasion via signalling from toll‐like receptor‐9 [39]. PANTHER assigned the ‘consensus’ genes to a range of protein classes, including protein‐modifying enzymes, gene‐specific transcriptional regulators, and both DNA and RNA metabolism proteins. The analysis also assigned biological pathways including signalling via integrins and p53 (Figure 3A,B). We performed GO and KEGG over‐representation analyses for the ‘union’ and ‘consensus’ gene‐lists, identifying significant enrichment in genes contributing to activation of GTPase activity and ribosome biogenesis (Table S8). Due to their contribution to cancer development [40, 41, 42, 43], identification of potential involvement of nuclear IGF‐1R in activation of these pathways demonstrates potential evidence as to why higher levels of nuclear IGF‐1R are associated with more advanced cancer stage in vivo.

### 3.5 Identification of a Consensus IGF‐1R Binding Motif

We also attempted to define a consensus IGF‐1R binding motif, using the XSTREME algorithm of MEME‐suite for novel motif discovery and enrichment analysis of known motifs [32, 44]. All 5743 of the unique IGF‐1R binding sites identified from RP biopsy samples were used as input sequences to identify significantly enriched motifs (*E‐*value < 0.05, Figure 3C, Table S9). Interestingly, the top‐ranked IGF‐1R binding motif shared similarity with the G‐rich binding motif identified from analysis of ~4000 peaks of INSR recruitment to the chromatin of human hepatocellular carcinoma cells [34] (Figure 3D). This is likely due to the structural homology of the closely related IGF‐1R and INSR receptors, and binding to a similar set of DNA binding sites may further contribute to their overlapping functions. To confirm this, further analysis is needed to assess the overlap of IGF‐1R DNA binding sites identified here with INSR DNA binding sites identified in the literature such as in [34]. Identification of a distinct set of binding sites between the two receptors would suggest distinct roles for the nuclear forms of the receptors, in contrast to the large overlap in their signalling pathways. Although the IGF‐1R antibody used in these ChIP‐seq experiments does not cross‐react with the INSR, it is possible that it may bind to IGF‐1R:INSR hybrid receptors. To assess the potential contribution of hybrid receptors, further studies could be carried out including IGF‐1R IP from fresh prostate tissue and reciprocal immunoblotting for INSR to assess levels of hybrid receptors, and further in vitro validation of the identified motif in cell‐based systems lacking either IGF‐1R or INSR.

The top ranked motif (Figure 3D) also shares similarity with the PITX2 transcription factor binding motif. Increased expression of PITX2 was previously shown to be associated with progression from normal prostate to metastatic disease and is upregulated in bone metastases vs. soft tissue metastases [45, 46]. This is of particular interest due to the contribution of IGFs to the development of bone metastasis, with previous work implicating IGFs in all stages of bone metastasis development and showing that high levels of IGF‐1 or IGF‐1R correlate with an increased tendency for bone metastasis [47, 48, 49]. The finding here showing similarity in the DNA binding motif of IGF‐1R and PITX2 suggests that there may be crosstalk between these two pathways via recruitment to a similar set of chromatin binding sites. This warrants further investigation that may confirm another pathway via which the IGF‐axis contributes to the development of bone metastasis. Overall, this would add further evidence to the clinical relevance of nuclear IGF‐1R and its association with advanced tumour stage.

It is also worth noting that one motif was identified as enriched in all 5743 IGF‐1R binding sites (Figure 3C), so it may represent an IGF‐1R specific binding motif. Validation experiments are now needed to confirm the binding of IGF‐1R to these motif sequences, such as luciferase promoter reporter assays, as with our previous work with the *JUN*, *FAM21A*, and *RRM2* promoters [15, 50], using the identified motif sequences cloned into these reporters and manipulation of IGF‐1R localisation.

### 3.6 In Vitro Validation of IGF‐1R Binding Sites

Finally, we undertook in vitro ChIP‐qPCR validation of the ChIP‐seq data obtained here. Previous work from our lab and others found that IGF‐1R targeting sensitises cells to DNA‐damaging cytotoxic drugs and ionising radiation (IR) in vitro and in vivo [51, 52, 53]. We further reported that IGF‐1R inhibition delays repair of IR‐induced DNA double‐strand breaks (DSB) and inhibits both homologous recombination (HR) and nonhomologous end‐joining (NHEJ) [53]. More recently, we showed that regulation of global replication by IGF‐1 is mediated via AKT, MEK/ERK, and JUN to influence expression of ribonucleotide reductase (RNR) subunit RRM2. Consequently, inhibition or depletion of IGF‐1R downregulates RRM2, compromising RNR function and perturbing dNTP supply, leading to delayed fork progression and replication stress [50].

**FIGURE 4:**
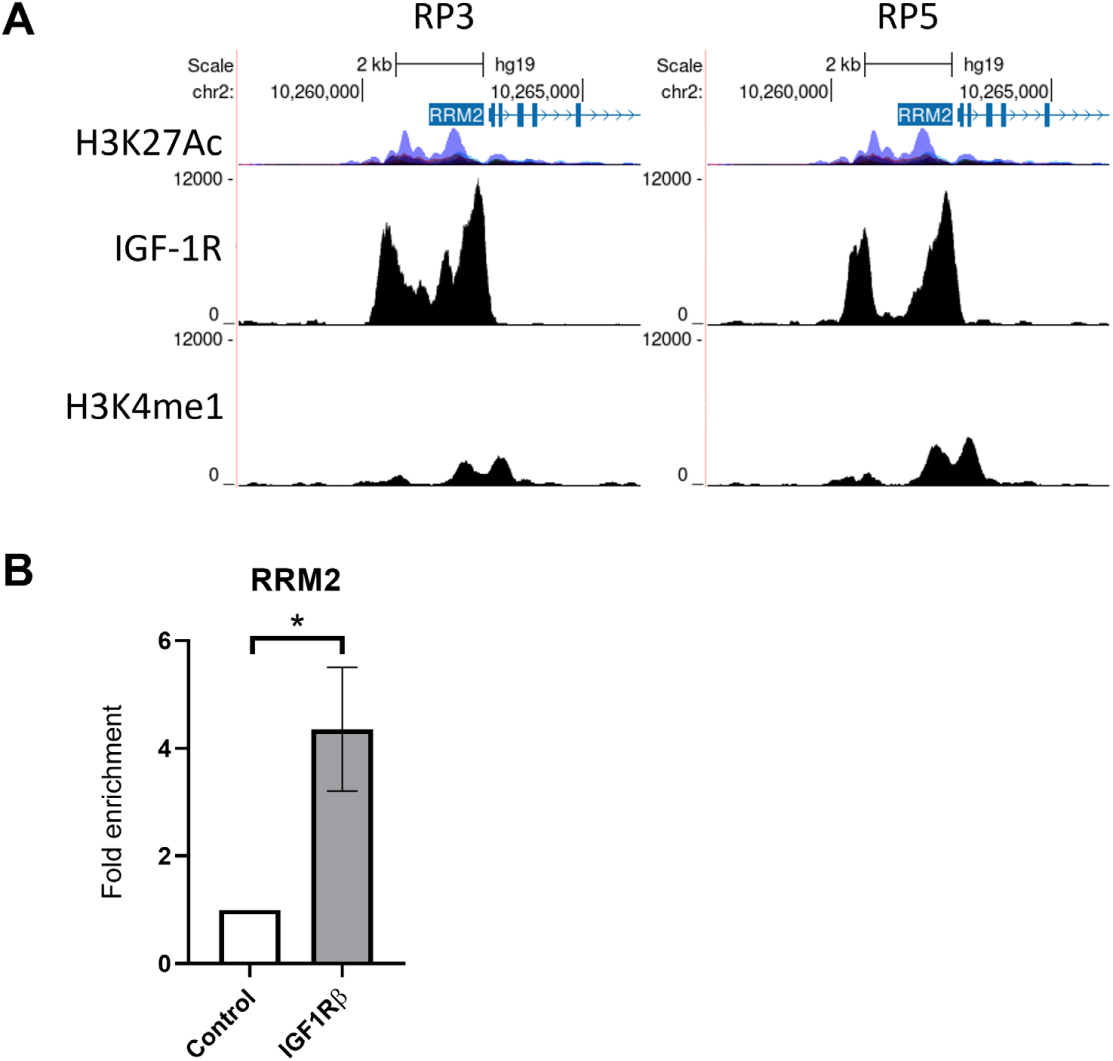
Characterisation of sites of IGF‐1R enrichment in clinical prostate tissues. (A) UCSC Genome Browser images showing IGF1R and H3K4me1 binding sites at the RRM2 TSS in RP3 (left) and RP5 (right) from ChIP‐seq data. Chromosome location is shown at the top of the image, with the location of *RRM2* highlighted in blue. H3K27Ac mark, often found near active regulatory elements, from ENCODE (https://genome.ucsc.edu/ENCODE/), is also shown. (B) IGF‐1R ChIP in DU145 cells showing significantly increased recruitment of IGF‐1R to the *RRM2* TSS (as shown in B). Graphs represent mean ± SEM fold enrichment over control (*p < 0.05, unpaired *t*‐test).

In the ChIP‐seq data obtained here, an IGF‐1R binding site was identified in the TSS of *RRM2* in 2 RP samples (RP3 and RP5) using the LanceOtron peak calling software. This peak was coincident with an H3K4me1 peak, a marker for an enhancer region, suggesting potential regulatory function (Figure 4A). Re‐analysis using LanceOtron of the ChIP‐seq data previously obtained from DU145 cells also detected an IGF‐1R binding site at the same region in the TSS of *RRM2*, which had not previously been identified during our initial analysis [15]. Therefore, due to the influence of the IGF‐axis on RRM2 function, this region was selected for ChIP‐qPCR validation.

IGF‐1R ChIP‐qPCR was performed on DU145 cells and tested using primers designed for the IGF‐1R binding sites detected in the *RRM2* TSS (see Section 2.4). Enriched recruitment of IGF‐1R was detected at a significantly increased level versus control, confirming recruitment of IGF‐1R to this region, validating the peak calling by LanceOtron and suggesting a regulatory role in RRM2 expression (Figure 4B).

Further functional validation is now needed to confirm that recruitment of IGF‐1R to this region is IGF‐1 dependent, and whether IGF‐1R recruitment influences RRM2 expression. This was not investigated in our previous work, as recruitment of IGF‐1R to *RRM2* was not detected in our initial ChIP‐seq analysis [15], highlighting the advantages of the more thorough peak calling analysis undertaken here. This functional validation could be performed using luciferase reporter plasmids containing the IGF‐1R binding sequence from the *RRM2* TSS, analogous to our previous work validating IGF‐1R binding sites at the *JUN* and *FAM21A* promoter regions [15]. Manipulation of IGF‐1R localisation and assessment of changes to *RRM2* expression or downstream functions such as RNR function and dNTP supply, as with our previous work [50], could also be used to assess the functional role of recruitment of IGF‐1R to *RRM2*. However, recruitment of IGF‐1R to *RRM2*, as validated here, suggests another method via which the IGF‐axis may play a regulatory role in the expression of RRM2 to influence DNA replication, highlighting the clinical relevance of nuclear IGF‐1R following detection of IGF‐1R recruitment to this region in vivo.

## 4 Conclusions

Detection of IGF‐1R peaks in multiple samples supports the concept that these regions play a consistent role in mediating actions of nuclear IGF‐1R. Their proximity to TSSs suggests IGF‐1R is binding to regulatory regions of DNA, potentially contributing to transcription, either directly or indirectly by influencing recruitment of other transcriptional regulators. The IGF‐dependent recruitment of RNAPolII we previously reported [15] represents one such example, and there may be others. Additional/alternative functions could include epigenetic regulation and scaffold/adaptor function. Future work is required to validate additional sites of IGF‐1R recruitment and test their IGF dependence and functional relevance, as well as the IGF‐1R DNA binding motifs identified. This will require a model in which IGF‐1R localisation can be manipulated, for example, using cells expressing SUMO‐site mutant IGF‐1R, identified by the Larsson group as able to signal from the cell surface but unable to translocate to the nucleus [13].

Overall, our data represent the first detection of IGF‐1R recruitment across the genome in clinical tissues and provide evidence to support the clinical relevance of nuclear IGF‐1R in vivo. Further functional validation will confirm whether there is a link between IGF‐1R recruitment and gene expression at the transcriptional and/or epigenetic level and shed further light on the association of nuclear IGF‐1R with more advanced PCa stage and its potential as a biomarker for IGF‐targeting cancer therapy.

## Author Contributions

**Jack V. Mills:** writing – original draft, writing – review and editing, methodology, data curation, formal analysis, validation, investigation, funding acquisition. **Avigail Taylor:** formal analysis, data curation. **Reema Singh:** validation, methodology. **Jinseon Kim:** methodology, validation. **Simon Engledow:** formal analysis. **Richard Colling:** resources. **Clare Verrill:** formal analysis. **Ian G. Mills:** data curation, writing – review and editing, supervision, funding acquisition. **Valentine M. Macaulay:** conceptualization, funding acquisition, writing – review and editing, project administration, supervision, data curation.

## Ethics Statement

All tissue samples were collected and used with the approval of the South Central—Oxford C Research Ethics Committee (REC reference 07/H0606/120) and the Oxford Radcliffe Biobank (ORB_20/A032), following relevant patient pre‐operative consent where applicable.

## Conflicts of Interest

The authors declare no conflicts of interest.

## Supporting information


**Data S1:** Supplementary Methods.
**Figure S1:** Quality control of ChIP‐seq data. (A) Example genomic regions with distinct and unique signal for IGF‐1R at a validated IGF‐1R binding site in an intergenic region of chromosome 17 (left), and for H3K4me1 at a known H3K4me1 binding site in the *SOX2* promoter (right). Genomic coordinates are indicated. (B) Distribution of peak widths in IGF‐1R and H3K4me1 ChIP‐seq datasets.
**Figure S2:** ChIP‐qPCR validation of IGF‐R recruitment to the *JUN* and *FAM21A* promoters in TURP and RP tissues.
**Figure S3:** IHC for total and phospho‐IGF‐1R on RPs analysed by ChIP‐seq. (A) Example RP stained for IGF‐1R, tumour area marked in green. Scale bar 5 mm. (B) Benign prostate glands in RP3 showing membrane and faint cytoplasmic IGF‐1R positivity. Scale bar 50 μm. (C) Total and phospho–IGF‐1R IHC in RP3 and RP6 showing representative areas adjacent to the punch biopsy used for ChIP‐seq. Scale bar 50 μm.
**Table S1:** Sequencing read count and quality parameters for each ChIP‐seq sample. Table shows, for each ChIP sample, the number of sequencing reads (*Total reads*), number of reads successfully aligned to the genome (*Reads aligned concordantly*) and number of non‐redundant reads (*Reads remaining after filtering*). For each sample quality parameters are also shown including the percentage of reads successfully mapped to the hg19 genome (*Mapping Ratio*) and the proportion of mapped reads that uniquely map to the genome (*Non‐redundant fraction*).
**Table S2:** TURP chippings collected for IGF‐1R ChIP. Table shows histology of parallel sample processed for routine histological analysis (i.e., not the same chippings processed for ChIP) and Gleason grade. TURP patients had rising PSA on endocrine therapy indicating CRPC. *Gleason score may not be reliable in patients on endocrine therapy. **Gleason grade not assigned.
**Table S3:** Numbers of peaks called in DU145 prostate cancer cells using three peak calling algorithms. Table shows the four datasets generated by re‐calling IGF‐1R peaks using MACS2‐narrow, MACS2‐broad and LanceOtron from duplicate independent IGF‐1R ChIP‐seq datasets (230, 237) against each of 2 independent control (IgG beads) ChIP‐seqs (225, 235) described in [2].
**Table S4:** Sites of IGF‐1R recruitment identified in cultured prostate cancer cells and clinical prostate cancers. Table shows chromosomal (Chr) location of IGF‐1R peaks identified in both re‐analysis of our previous DU145 cell line ChIP‐seq (Aleksic et al., 2018) and identified in ChIP‐seq performed here from fresh RP tissue samples, using MACS2 narrow (blue, 20 peaks) and broad (green, 27) parameters and LanceOtron (yellow, 908). Also shown are annotation of relevant region, distance of peak from nearest TSS, gene name, gene ID and transcript ID. Peaks detected in multiple callers are shown in the relevant colour for each one.
**Table S5:** Sites of IGF‐1R enrichment coincident with ARBSs in clinical prostate cancers. Table shows 407 IGF‐1R peaks identified here in clinical prostate cancers by ChIP‐seq that overlap with ARBSs detected in two cases of treatment naïve prostate cancer tissue by (Sharma et al., 2013). Table shows chromosome coordinates for each peak, annotation of relevant region, distance of peak from nearest TSS, gene name, gene ID and transcript ID.
**Table S6:** Numbers of genes with proximal peak of IGF‐1R recruitment. Table shows numbers of genes per individual tissue sample, union gene set of sites proximal to the TSS of any gene in any sample, and ‘consensus’ genes with a TSS proximal to sites of IGF‐1R recruitment detected by any peak caller in more than one tissue.
**Table S7:** Genes containing IGF‐1R peak proximal to their TSS in 4 or more samples. Table shows number of samples that contained an IGF‐1R peak within gene TSS, gene name and basic description of gene function. From GeneCards: The Human Gene Database (https://www.genecards.org).
**Table S8:** Pathways significantly over‐represented in union and consensus gene‐sets. GO enrichment analysis did not identify significantly over‐represented pathways in the Consensus gene set, and KEGG enrichment analysis did not identify significantly over‐represented pathways in the union gene set.
**Table S9:** IGF‐1R DNA binding motifs identified using MEME‐suite. Table showing significantly enriched de novo motifs identified using the MEME‐suite XSTREME algorithm of the 5743 unique IGF‐1R binding sites identified in RP biopsies. *E*‐values for each motif are shown along with the number of occurrences of the motif within each individual peak sequence and the percentage of peak sequences that the motif was identified in. Known or similar motifs from the human Hocomoco v11 database identified by the Tomtom algorithm are also shown.

## Data Availability

The FASTQ files generated from the ChIP‐seq in this paper will be deposited in the GEO repository. Further data that support the findings of this study are available from the corresponding author upon reasonable request.
