# Supplementary material for: Detection of Genome‐Wide IGF‐1R Recruitment to Enhancer and Promoter Regions of Chromatin in Clinical Prostate Cancers
Source: Cancer Med. 2025 Sep 30;14(19):e71257. doi: 10.1002/cam4.71257 (PMC12483945; doi:10.1002/cam4.71257)
Supplement: Supplementary file 1 — Data S1: Supplementary Methods. Figure S1: Quality control of ChIP‐seq data. (A) Example genomic regions with distinct and unique signal for IGF‐1R at a validated IGF‐1R binding site in an intergenic region of chromosome 17 (left), and for H3K4me1 at a known H3K4me1 binding site in the SOX2 promoter (right). Genomic coordinates are indicated. (B) Distribution of peak widths in IGF‐1R and H3K4me1 ChIP‐seq datasets. Figure S2: ChIP‐qPCR validation of IGF‐R recruitment to the JUN and FAM21A promoters in TURP and RP tissues. Figure S3: IHC for total and phospho‐IGF‐1R on RPs analysed by ChIP‐seq. (A) Example RP stained for IGF‐1R, tumour area marked in green. Scale bar 5 mm. (B) Benign prostate glands in RP3 showing membrane and faint cytoplasmic IGF‐1R positivity. Scale bar 50 μm. (C) Total and phospho–IGF‐1R IHC in RP3 and RP6 showing representative areas adjacent to the punch biopsy used for ChIP‐seq. Scale bar 50 μm. Table S1: Sequencing read count and quality parameters for each ChIP‐seq sample. Table shows, for each ChIP sample, the number of sequencing reads (Total reads), number of reads successfully aligned to the genome (Reads aligned concordantly) and number of non‐redundant reads (Reads remaining after filtering). For each sample quality parameters are also shown including the percentage of reads successfully mapped to the hg19 genome (Mapping Ratio) and the proportion of mapped reads that uniquely map to the genome (Non‐redundant fraction). Table S2: TURP chippings collected for IGF‐1R ChIP. Table shows histology of parallel sample processed for routine histological analysis (i.e., not the same chippings processed for ChIP) and Gleason grade. TURP patients had rising PSA on endocrine therapy indicating CRPC. *Gleason score may not be reliable in patients on endocrine therapy. **Gleason grade not assigned. Table S3: Numbers of peaks called in DU145 prostate cancer cells using three peak calling algorithms. Table shows the four datasets generated by re‐callin [file CAM4-14-e71257-s001.docx]

**Mills et al: Supporting Information**

# *Supplementary methods*

**1.1 Optimisation of ChIP for prostate cancer tissue**. Initial optimisation was performed using fresh TURP tissue from three CRPC patients. Two samples contained admixed benign and cancer, and one was reported as benign despite coming from a patient with CRPC (**Supplementary Table S2)**. IGF-1R recruitment to the *JUN* and *FAM21A* promoters was detected in all TURPs (**Supplementary Figure S2A).** Using a method adapted from [[1](#_ENREF_1)], chromatin was extracted from RP biopsies and RP1 was used in optimisation of tissue cutting, fixation and sonication. We analysed 50 µg chromatin from the RP4 benign punch by western blot for IGF-1R and Histone H3, confirming their presence for detection in ChIP (**Supplementary Figure S2B**). The remainder of this sample was processed by ChIP for enhancer mark H3K4me1, previously detected at the *JUN* and *FAM21A* promoters in PCa cells [[2](#_ENREF_2)] and successfully detected here by ChIP-qPCR (**Supplementary Figure S2C**). Following confirmation that the ChIP protocol was performing correctly, we processed three tissue biopsies for IGF-1R ChIP, but IGF-1R enrichment was not detected by qPCR at the *JUN* promoter (**Supplementary Figure S2D**). To check whether this was a genuine negative or a technical issue, we used the remaining 3 malignant samples in ChIP-qPCR for IGF-1R with H3K4me1 as positive control. IGF-1R recruitment was detectable at the *FAM21A* promoter in only one (RP5; **Supplementary Figure S2D-E)**, but H3K4me1 enrichment was detected in all three RPs suggesting the protocol performed acceptably using small amounts of input material.

**1.2 ChIP-seq data analysis and quality control.** Peaks were called using three algorithms: MACS2 ‘narrow’, MACS2 ‘broad’ and LanceOtron [[3](#_ENREF_3),[4](#_ENREF_4)] as outlined in Section 2.5 of Methods. For each sample, peaks were called in the IGF-1R pulldown ChIP-seq data filtering against a corresponding ‘beads only’ pulldown control sample to remove non-specific peak signals. Peaks that had been aligned to an unmapped contig in the hg19 build were also removed. Within this framework, the following program-specific parameters were set: (1) For MACS2 with default ‘narrow’ peak calling arguments a cut off of FDR < 0.05, was used for IGF-1R pulldown peaks. (2) For MACS2-broad, the FDR threshold for inner peaks was also set at 0.05 but the FDR threshold for broad peaks (--broad-cutoff) was kept at the default value of 0.1. (3) For each putative peak, LanceOtron provides a Peak Score metric which is “the probability of the assessed region’s signal arising from a biological event”, and the authors recommend using this Peak Score to filter peaks instead of an adjusted p-value. With this is in mind, IGF-1R peaks were filtered to include only those that had a Peak Score > 0.8. Prior to analysis, peaks were uploaded to and visualised using the UCSC genome browser and visual inspection of regions previously shown to bind IGF-1R or known to contain H3K4me1 modifications was performed (http://genome.ucsc.edu). Examples of regions are shown in Supplementary Figure 1, with detection of an IGF-1R peak previously identified and validated *in vitro* at an intergenic region of chromosome 17 [[5](#_ENREF_5)] (Supplementary Figure 1A, left), and detection of a H3K4me1 peak at a known binding site in the promoter of SOX2 [[6](#_ENREF_6),[7](#_ENREF_7)] (Supplementary Figure 1A, right). Distinct and unique peak signals in each case support the validity of the ChIP-seq data. The peaks of H3K4me1 histone modification are often a mixture of sharp narrow peaks and broader peaks, depending on genomic localistation [[8-10](#_ENREF_8)]. Here, peaks for H3K4me1 show a wider spread of peak size compared to peaks detected for IGF-1R, and this spread incorporates both narrow and broader peak sizes as shown by the density distributions in Supplementary Figure 1B. Identfiication of peaks within the expected peak size ranges also supports the validity of the ChIP-seq data obtained here.

While there is not complete consensus on the quality control metrics for ChIP-enrichment and ChIP-seq, commonly the number of reads and peaks detected are reported [[11-13](#_ENREF_11)]. The total number of reads and number of uniquely aligned reads for each ChIP-seq sample are shown in Supplementary Table 1, with corresponding number of peaks for each sample shown and reported in Figure 1 of the main text. Also shown in Supplementary Table 1 are the Mapping ratio and Non-redundant fraction (NRF) for each sample, two quality control measures also suggested by the ENCODE consortium for assessing ChIP-seq data quality [[11](#_ENREF_11),[13](#_ENREF_13)]. However, it is worth noting that these guidelines have been developed in the analysis of data obtained from *in vitro* experiments. Therefore, due to intrinsic heterogeneity within tumour samples, and the further biological variation between tumour samples obtained from different patient sources, it is unlikely that tumour derived ChIP-seq data will be able to fully meet these stringent guidelines.

The ENCODE consortium suggests at least 10 million uniquely mapped reads as a minimum to analyze peaks in human samples, with a larger number (e.g. >40 million) suggested for histone marks [[11](#_ENREF_11),[14](#_ENREF_14)]. All ChIP samples generated here meet this quality control criteria (Supplementary Table 1). However, not all of the samples meet the suggested thresholds of Mapping ratio (≥70%)[[15](#_ENREF_15)] and NRF (≥ 0.8)[[11](#_ENREF_11)]. The data for the Input sample and all H3K4me1 ChIP samples successfully meet these criteria, with all showing a mapping ratio of at least 80% or higher, and all showing NRF ≥ 0.8. However, of the IGF-1R ChIP samples, only RP4 shows a Mapping ratio above the 70% threshold (83.65%) and none of the samples meet the threshold of NRF ≥ 0.8 (RP4 shows the highest at 0.78) (Supplementary Table 1). However, traditionally non-DNA-binding proteins, such as IGF-1R, are known to often have a lower mapping ratio of only approximately 60% [[16](#_ENREF_16)] and the majority of IGF-1R ChIP samples processed here meet this threshold.

Overall, the quality metrics for IGF-1R ChIP-seq samples are lower than those for the H3K4me1 ChIP samples, however this is not surprising due to a variety of factors. Firstly, ChIP of histone marks is more efficient as histones are a vital structural component of chromatin [[17-19](#_ENREF_17)]. ChIP of histone marks is robust and highly reproducible [[19](#_ENREF_19),[20](#_ENREF_20)], leading to the selection of H3K4me1 ChIP as a positive control for successful ChIP-seq workflow in this work. Secondly, IGF-1R is not a traditional transcription factor and has only relatively recently been identified to interact with chromatin [[2](#_ENREF_2)]. The lower prevalence of this interaction is therefore highly likely to reduce the efficiency of IGF-1R ChIP. Thirdly, translocation of IGF-1R to the nucleus and subsequent interaction with chromatin has been shown to be IGF dependent [[2](#_ENREF_2)]. This therefore means IGF-1R interactions with chromatin are dependent on physiological levels of IGF which are highly likely to vary from patient to patient and localised levels of IGF may vary even within tumour [[21](#_ENREF_21),[22](#_ENREF_22)]. This can further reduce levels of IGF-1R – chromatin interactions and therefore reduce IGF-1R ChIP efficiency. In future work, if possible, it would be useful to obtain measures of physiological levels of IGF from tissue-donating patients to assess this. Finally, high quality ChIP-seq datasets with lower numbers of genuine binding sites, as is likely the case with IGF-1R, may produce lower quality control values [[11](#_ENREF_11)]. Overall, the fact that all positive control H3K4me1 ChIP samples in this work meet the quality control criteria outlined previously gives more confidence in the quality of the workflow and therefore data produced from ChIP IGF-1R samples that were processed in parallel.

Identified IGF-1R peaks that were found in at least two tumor samples were considered for analysis, resulting in 5743 consensus peaks of IGF-1R binding. This high number of consensus peaks, and the validation of recruitment of IGF-1R to identified peaks (Figure 3, main text) further supports the quality of the data.

**1.3 Re-analysis of ChIP-seq data from DU145 prostate cancer cells**. From our previous work analysing DU145 prostate cancer cells (RRID: CVCL_0105) [[2](#_ENREF_2)], ChIP-seq data had been obtained from four DU145 cell-line samples: two of which had been processed for ChIP using antibodies to IGF-1R (termed “IGF-1R pulldown ChIP-seq DU145” samples), and two control samples processed for ChIP using Immunoglobulin G (IgG) antibody (termed “IgG pulldown ChIP-seq DU145” samples). Mirroring the workflow for the analysis of ChIP-seq data of RP biopsies, ChIP-Seq reads were mapped using Bowtie2 [[23](#_ENREF_23)] and aligned to the human reference genome hg19. Then, as for the RP biopsies data, MACS2 [[3](#_ENREF_3)] was used with default ‘narrow’ peak calling arguments, MACS2 with ‘broad’ peak calling switched on, and LanceOtron [[4](#_ENREF_4)] to call peaks of IGF-1R enrichment in the aligned reads. The same program-specific parameters used for the analysis of the RP biopsies data were set, as described above. With each algorithm, peaks were called in the two IGF-1R pulldown DU145 ChIP-seq samples twice, once with each of the two IgG pulldown ChIP-seq DU145 samples as a control, thus yielding four sets of peak calls from each of the three algorithms. For each algorithm, these four sets of peaks were merged using the BEDTools merge sub-command [[24](#_ENREF_24)] with default settings, such that overlapping and “book-ended” peaks were combined into a single peak spanning all of the combined peaks. Thus, three sets of merged IGF-1R binding peaks were derived from the DU145 cell line data; one from each of the three employed algorithms. To estimate the overlap in peak sets called by MACS2, MACS2-broad and LanceOtron, the BEDTools merge sub-command with default settings was used to combine the three sets of merged IGF-1R peaks obtained from the individual algorithms. Next, each of the resultant combined peaks were annotated to denote whether it was comprised of peaks from just one of the algorithms, peaks from all three algorithms, or peaks from: MACS2 & MACS2-broad; MACS2 & LanceOtron; or MACS2-broad & LanceOtron. The number of combined peaks in each annotation category were then reported.

**1.4** **Within algorithm comparison of ChIP-seq peaks called in RP biopsies versus prostate cancer cells**. For each of MACS2, MACS2-broad and LanceOtron the overlap of peaks called in prostate cancer tissues versus peaks called in prostate cancer cells was assessed. Firstly, the set of merged IGF-1R binding peaks obtained with a specific peak calling program (MACS2, MACS2-broad or LanceOtron) in the RP biopsies was merged with the corresponding set of merged IGF-1R binding peaks obtained with that the same program in the DU145 cells. As before, this was done using the BEDTools merge sub-command with default settings. Each of the resultant combined peaks was then annotated to denote whether it was comprised of peaks from just tissue, peaks from just cells, or peaks from both. the number of combined peaks in each of these three annotation categories was then reported.

# *Supplementary Figures*


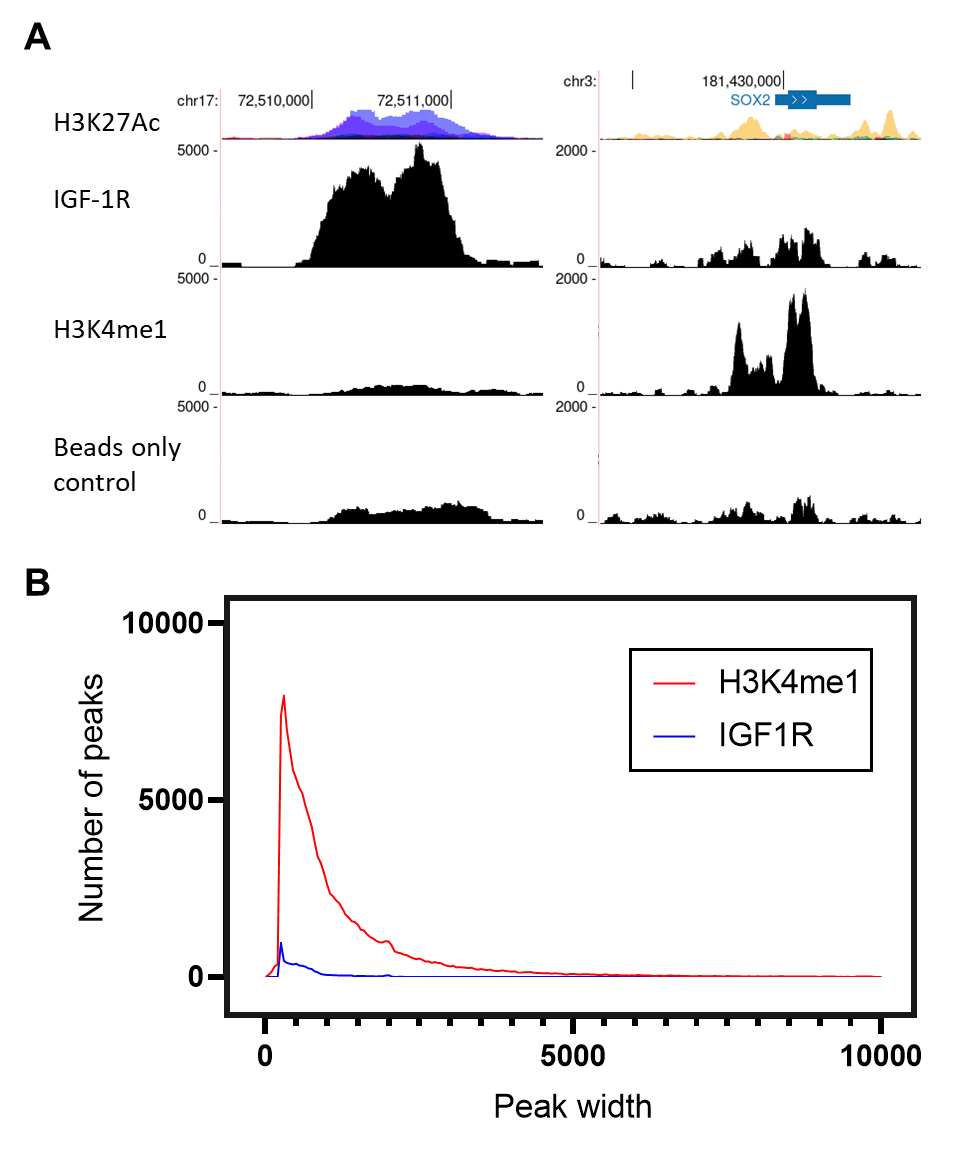


**Supplementary Figure S1: Quality control of ChIP-seq data. A.** Example genomic regions with distinct and unique signal for IGF-1R at a validated IGF-1R binding site in an intergenic region of chromosome 17 (left), and for H3K4me1 at a known H3K4me1 binding site in the *SOX2* promoter (right). Genomic coordinates are indicated. **B.** Distribution of peak widths in IGF-1R and H3K4me1 ChIP-seq datasets.

**
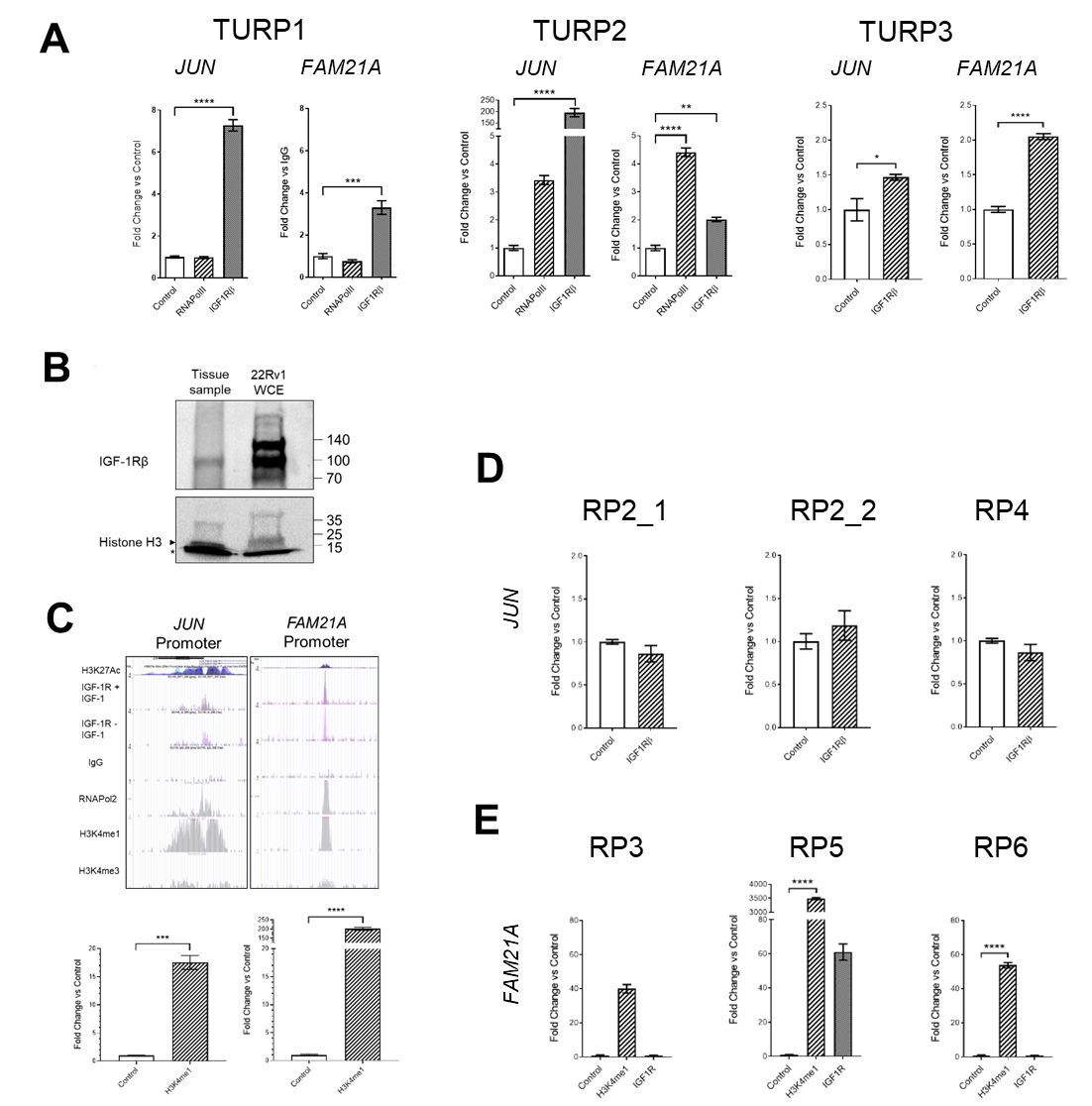
**

**Supplementary Figure S2: ChIP-qPCR validation of IGF-R recruitment to the *JUN* and *FAM21A* promoters in TURP and RP tissues.**

**A.** Graphs show ChIP-qPCR quantification of recruitment of RNAPolII and IGF-1R to the *JUN* and *FAM21A* promoters in 3 TURP tissues. Results represent mean ± SEM fold change vs control (beads only) in 3 technical replicates. **B.** Western blot for IGF-1Rβ and Histone H3 in chromatin extracted from RP4 benign biopsy alongside 22Rv1 whole cell extract (WCE) as positive control. Arrowhead: histone H3. Asterisk: signal likely due to dye front.  **C.** UCSC genome browser image from (Aleksic et al., 2018) showing recruitment of IGF-1R and H3K4me1 to the *JUN* and *FAM21A* promoters. Graphs below: ChIP-qPCR quantification showing enrichment of H3K4me1 at both the *JUN* and *FAM21A* promoters in the RP4 benign biopsy analysed in B. Results represent mean ± SEM of triplicate technical replicates (*p<0.05, **p<0.005, *** p< 0.0005, **** p< 0.0001 by unpaired t test to compare 2 samples and one-way ANOVA for >2). **D.** Graphs: ChIP-qPCR for IGF-1R recruitment to *JUN* promoter. Lack of significant enrichment was confirmed by unpaired t test. **E.** ChIP-qPCR to quantify IGF-1R and H3K4me1 enrichment at the *FAM21A* promoter. Results in D and E represent mean ± SEM of 3 technical replicates. H3K4me1 enrichment was detected in all RP biopsies but  IGF-1R enrichment was detected only on the *FAM21A* promoter of RP5 (****p< 0.0001 by one-way ANOVA).

**
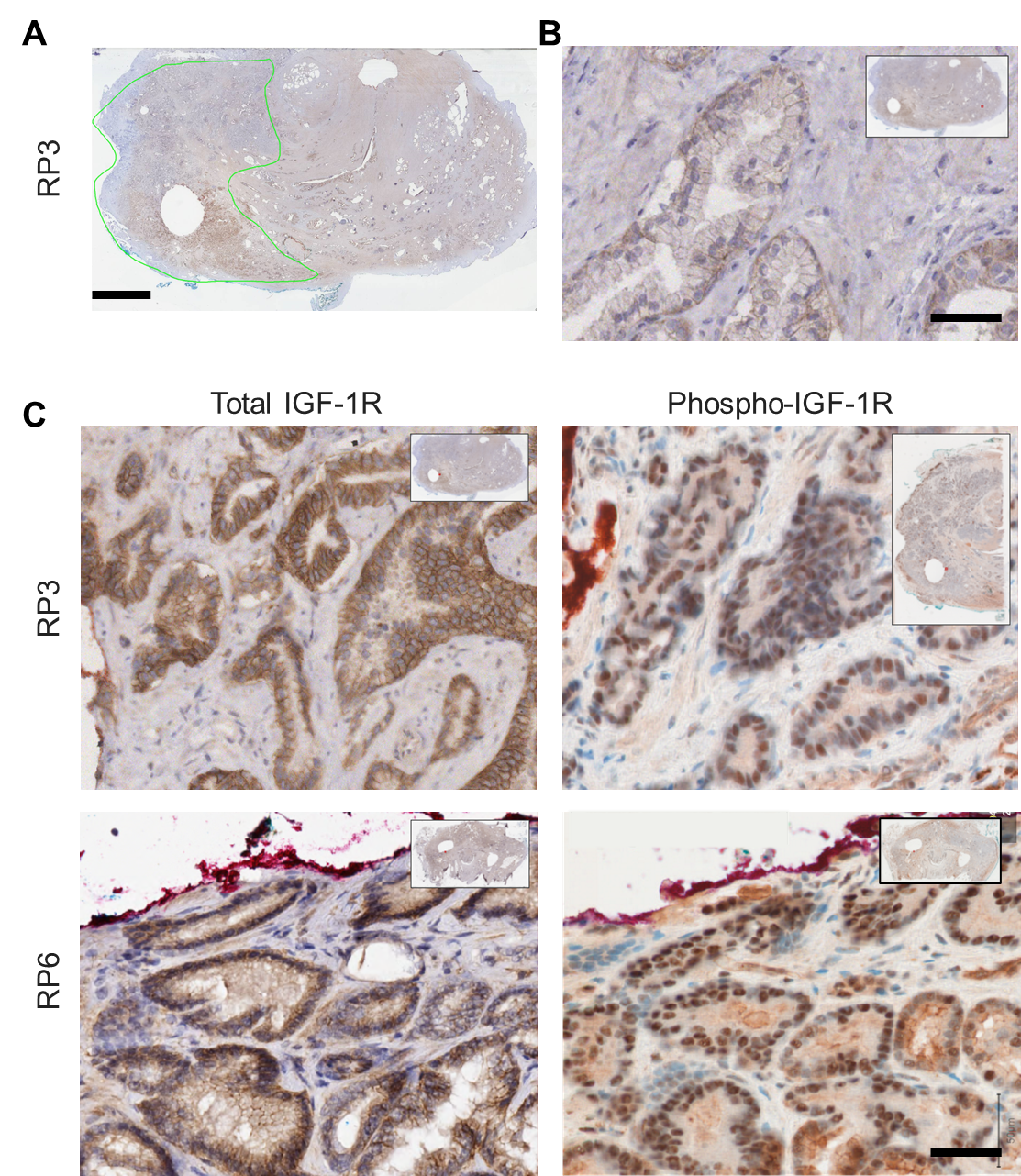
**

**Supplementary Figure S3: IHC for total and phospho-IGF-1R on RPs analysed by ChIP-seq. A.** Example RP stained for IGF-1R, tumour area marked in green. Scale bar 5mm. **B.** Benign prostate glands in RP3 showing membrane and faint cytoplasmic IGF-1R positivity. Scale bar 50µm. **C.** Total and phospho –IGF-1R IHC in RP3 and RP6 showing representative areas adjacent to the punch biopsy used for ChIP-seq. Scale bar 50µm.

*Supplementary Tables*

| ***Sample Name*** | ***Total reads*** | ***Reads aligned concordantly*** | ***Reads remaining after filtering*** | ***Mapping Ratio (%)*** | ***Non-redundant fraction*** |
| --- | --- | --- | --- | --- | --- |
| Input control | 60782829 | 52130666 | 42264484 | 85.77 | 0.81 |
|  |  |  |  |  |  |
| ***Beads only ChIP sample*** | ***Total reads*** | ***Reads aligned concordantly*** | ***Reads remaining after filtering*** | ***Mapping Ratio (%)*** | ***Non-redundant fraction*** |
| RP2_1 Control | 44298628 | 24391864 | 8929403 | 55.06 | 0.37 |
| RP2_2 Control | 50661500 | 30984843 | 22581039 | 61.16 | 0.73 |
| RP3 Control | 54013666 | 40061961 | 24694195 | 74.17 | 0.62 |
| RP4 Control | 52355317 | 42372074 | 28448850 | 80.93 | 0.67 |
| RP5 Control | 63092040 | 44545237 | 30142379 | 70.60 | 0.68 |
| RP6 Control | 40723744 | 20611830 | 8036392 | 50.61 | 0.39 |
|  |  |  |  |  |  |
| ***H3K4me1 ChIP sample*** | ***Total reads*** | ***Reads aligned concordantly*** | ***Reads remaining after filtering*** | ***Mapping Ratio (%)*** | ***Non-redundant fraction*** |
| RP3 H3K4me1 | 58334211 | 47853350 | 40380788 | 82.03 | 0.84 |
| RP5 H3K4me1 | 51739573 | 43468634 | 34914870 | 84.01 | 0.80 |
| RP6 H3K4me1 | 59005228 | 50280484 | 40707010 | 85.21 | 0.81 |
|  |  |  |  |  |  |
| ***IGF-1R ChIP sample*** | ***Total reads*** | ***Reads aligned concordantly*** | ***Reads remaining after filtering*** | ***Mapping Ratio (%)*** | ***Non-redundant fraction*** |
| RP2_1 IGF-1R | 52821645 | 25338994 | 11129482 | 47.97 | 0.44 |
| RP2_2 IGF-1R | 56014153 | 34472140 | 24305796 | 61.54 | 0.71 |
| RP3 IGF-1R | 48586983 | 29525103 | 10993998 | 60.77 | 0.37 |
| RP4 IGF-1R | 48368336 | 40461772 | 31504471 | 83.65 | 0.78 |
| RP5 IGF-1R | 49222416 | 29131461 | 18089419 | 59.18 | 0.62 |
| RP6 IGF-1R | 47052075 | 25391583 | 13198104 | 53.96 | 0.52 |

**Supplementary Table S1: Sequencing read count and quality parameters for each ChIP-seq sample**. Table shows, for each ChIP sample, the number of sequencing reads (*Total reads*), number of reads successfully aligned to the genome (*Reads aligned concordantly*) and number of non-redundant reads (*Reads remaining after filtering*). For each sample quality parameters are also shown including the percentage of reads successfully mapped to the hg19 genome (*Mapping Ratio*) and the proportion of mapped reads that uniquely map to the genome (*Non-redundant fraction*).

| ***Sample Number*** | ***Histology*** | ***Gleason Grade*** | ***Grade Group*** | ***CRPC?*** |
| --- | --- | --- | --- | --- |
| TURP 1 | >80% of chippings contain prostatic adenocarcinoma | 4+5 = 9* | 5* | Yes |
| TURP 2 | ~50% of chippings contain prostatic adenocarcinoma | N/A** | N/A** | Yes |
| TURP 3 | Benign | - | - | Yes |

**Supplementary Table S2: TURP chippings collected for IGF-1R ChIP**. Table shows histology of parallel sample processed for routine histological analysis (i.e. not the same chippings processed for ChIP) and Gleason grade. TURP patients had rising PSA on endocrine therapy indicating CRPC. *Gleason score may not be reliable in patients on endocrine therapy. **Gleason grade not assigned.

| ***Sample*** | ***Input*** | ***ChIP-seq peaks (n)*** | | |
| --- | --- | --- | --- | --- |
|  |  | ***MACS2-narrow*** | ***MACS2-broad*** | ***LanceOtron*** |
| IGF1R_1 (230) | IgG_1 (225) | 139 | 215 | 1665 |
| IGF1R_1 (230) | IgG_2 (235) | 27 | 57 | 915 |
| IGF1R_2 (237) | IgG_1 (225) | 115 | 165 | 1008 |
| IGF1R_2 (237) | IgG_2 (235) | 25 | 38 | 707 |

**Supplementary Table S3: Numbers of peaks called in DU145 prostate cancer cells using three peak calling algorithms**. Table shows the 4 datasets generated by re-calling IGF-1R peaks using MACS2-narrow, MACS2-broad and LanceOtron from duplicate independent IGF-1R ChIP-seq datasets (230, 237) against each of 2 independent control (IgG beads) ChIP-seqs (225, 235) described in [[2](#_ENREF_2)].

| ***Chrom*** | ***Start*** | ***End*** | ***Annotation*** | ***Distance to TSS (kb)*** | ***Gene Name*** | ***Gene ID*** | ***Transcript ID*** |
| --- | --- | --- | --- | --- | --- | --- | --- |
| 5 | 415547 | 415732 | Intron (ENST00000505113.1/ENSG00000063438.12, intron 5 of 10) | -3.086 | CTD-2228K2.1 | ENSG00000250645.1 | ENST00000509301.1 |
| 8 | 43094685 | 43094766 | Distal Intergenic | 7.312 | RP11-726G23.2 | ENSG00000253884.1 | ENST00000518796.1 |
| 8 | 43094766 | 43094982 | Distal Intergenic | 7.096 | RP11-726G23.2 | ENSG00000253884.1 | ENST00000518796.1 |
| 8 | 43094982 | 43094990 | Distal Intergenic | 7.088 | RP11-726G23.2 | ENSG00000253884.1 | ENST00000518796.1 |
| 8 | 43092895 | 43093138 | Distal Intergenic | 8.94 | RP11-726G23.2 | ENSG00000253884.1 | ENST00000518796.1 |
| 8 | 43093138 | 43093152 | Distal Intergenic | 8.926 | RP11-726G23.2 | ENSG00000253884.1 | ENST00000518796.1 |
| 17 | 72510352 | 72510386 | Distal Intergenic | 17.219 | CD300LB | ENSG00000178789.4 | ENST00000392621.1 |
| 17 | 72510386 | 72510417 | Distal Intergenic | 17.188 | CD300LB | ENSG00000178789.4 | ENST00000392621.1 |
| 17 | 72510417 | 72510666 | Distal Intergenic | 16.939 | CD300LB | ENSG00000178789.4 | ENST00000392621.1 |
| 17 | 72510146 | 72510352 | Distal Intergenic | 17.253 | CD300LB | ENSG00000178789.4 | ENST00000392621.1 |
| 17 | 19091348 | 19091458 | Promoter (<=1kb) | 0.02 | SNORD3A | ENSG00000263934.2 | ENST00000584923.1 |
| 17 | 19091458 | 19091484 | Promoter (<=1kb) | 0.13 | SNORD3A | ENSG00000263934.2 | ENST00000584923.1 |
| 17 | 72510124 | 72510146 | Distal Intergenic | 17.459 | CD300LB | ENSG00000178789.4 | ENST00000392621.1 |
| 17 | 72510666 | 72510712 | Distal Intergenic | 16.893 | CD300LB | ENSG00000178789.4 | ENST00000392621.1 |
| 17 | 72510712 | 72510908 | Distal Intergenic | 16.697 | CD300LB | ENSG00000178789.4 | ENST00000392621.1 |
| 21 | 9826128 | 9826223 | Promoter (<=1kb) | 0 | MIR3687 | ENSG00000264063.1 | ENST00000577708.1 |
| 21 | 9826223 | 9826382 | Promoter (<=1kb) | 0.021 | MIR3687 | ENSG00000264063.1 | ENST00000577708.1 |
| 21 | 9825663 | 9825785 | Promoter (<=1kb) | -0.047 | MIR3648 | ENSG00000264462.1 | ENST00000581792.1 |
| 21 | 9825785 | 9825845 | Promoter (<=1kb) | 0 | MIR3648 | ENSG00000264462.1 | ENST00000581792.1 |
| 21 | 9825845 | 9826128 | Promoter (<=1kb) | 0.014 | MIR3648 | ENSG00000264462.1 | ENST00000581792.1 |
| 1 | 59249630 | 59249738 | Promoter (<=1kb) | 0.047 | JUN | ENSG00000177606.5 | ENST00000371222.2 |
| 1 | 59249738 | 59249769 | Promoter (<=1kb) | 0.016 | JUN | ENSG00000177606.5 | ENST00000371222.2 |
| 1 | 59249769 | 59249868 | Promoter (<=1kb) | 0 | JUN | ENSG00000177606.5 | ENST00000371222.2 |
| 2 | 1218256 | 1218382 | Intron (ENST00000308624.5/ENSG00000172554.7, intron 9 of 16) | 13.442 | SNTG2 | ENSG00000172554.7 | ENST00000498321.1 |
| 5 | 415545 | 415769 | Intron (ENST00000505113.1/ENSG00000063438.12, intron 5 of 10) | -3.049 | CTD-2228K2.1 | ENSG00000250645.1 | ENST00000509301.1 |
| 8 | 43092791 | 43092814 | Distal Intergenic | 9.264 | RP11-726G23.2 | ENSG00000253884.1 | ENST00000518796.1 |
| 8 | 43092814 | 43093139 | Distal Intergenic | 8.939 | RP11-726G23.2 | ENSG00000253884.1 | ENST00000518796.1 |
| 8 | 43093139 | 43093152 | Distal Intergenic | 8.926 | RP11-726G23.2 | ENSG00000253884.1 | ENST00000518796.1 |
| 8 | 43094683 | 43095219 | Distal Intergenic | 6.859 | RP11-726G23.2 | ENSG00000253884.1 | ENST00000518796.1 |
| 8 | 43095219 | 43095233 | Distal Intergenic | 6.845 | RP11-726G23.2 | ENSG00000253884.1 | ENST00000518796.1 |
| 8 | 43096831 | 43097052 | Distal Intergenic | 5.026 | RP11-726G23.2 | ENSG00000253884.1 | ENST00000518796.1 |
| 15 | 44580649 | 44580752 | Promoter (<=1kb) | -0.175 | CASC4 | ENSG00000166734.14 | ENST00000559222.1 |
| 15 | 86439555 | 86439679 | Distal Intergenic | 62.523 | RP11-23A22.1 | ENSG00000259608.1 | ENST00000561364.1 |
| 17 | 72510171 | 72510789 | Distal Intergenic | 16.816 | CD300LB | ENSG00000178789.4 | ENST00000392621.1 |
| 17 | 72510789 | 72510835 | Distal Intergenic | 16.77 | CD300LB | ENSG00000178789.4 | ENST00000392621.1 |
| 17 | 72510835 | 72510976 | Distal Intergenic | 16.629 | CD300LB | ENSG00000178789.4 | ENST00000392621.1 |
| 17 | 72510124 | 72510146 | Distal Intergenic | 17.459 | CD300LB | ENSG00000178789.4 | ENST00000392621.1 |
| 17 | 72510146 | 72510171 | Distal Intergenic | 17.434 | CD300LB | ENSG00000178789.4 | ENST00000392621.1 |
| 17 | 19091346 | 19091359 | Promoter (<=1kb) | 0.018 | SNORD3A | ENSG00000263934.2 | ENST00000584923.1 |
| 17 | 19091359 | 19091458 | Promoter (<=1kb) | 0.031 | SNORD3A | ENSG00000263934.2 | ENST00000584923.1 |
| 17 | 19091458 | 19091484 | Promoter (<=1kb) | 0.13 | SNORD3A | ENSG00000263934.2 | ENST00000584923.1 |
| 17 | 19091484 | 19091553 | Promoter (<=1kb) | 0.156 | SNORD3A | ENSG00000263934.2 | ENST00000584923.1 |
| 21 | 9825864 | 9826223 | Promoter (<=1kb) | 0 | MIR3687 | ENSG00000264063.1 | ENST00000577708.1 |
| 21 | 9826223 | 9826382 | Promoter (<=1kb) | 0.021 | MIR3687 | ENSG00000264063.1 | ENST00000577708.1 |
| 21 | 9826858 | 9827047 | Promoter (<=1kb) | 0.656 | MIR3687 | ENSG00000264063.1 | ENST00000577708.1 |
| 21 | 9825658 | 9825659 | Promoter (<=1kb) | -0.173 | MIR3648 | ENSG00000264462.1 | ENST00000581792.1 |
| 21 | 9825659 | 9825864 | Promoter (<=1kb) | 0 | MIR3648 | ENSG00000264462.1 | ENST00000581792.1 |
| 1 | 2.37E+08 | 2.37E+08 | Intron (ENST00000542672.1/ENSG00000077522.8, intron 1 of 20) | -17.472 | ACTN2 | ENSG00000077522.8 | ENST00000546208.1 |
| 1 | 2.37E+08 | 2.37E+08 | Intron (ENST00000542672.1/ENSG00000077522.8, intron 1 of 20) | -17.282 | ACTN2 | ENSG00000077522.8 | ENST00000546208.1 |
| 1 | 2.37E+08 | 2.37E+08 | Intron (ENST00000542672.1/ENSG00000077522.8, intron 1 of 20) | -16.348 | ACTN2 | ENSG00000077522.8 | ENST00000546208.1 |
| 1 | 2.37E+08 | 2.37E+08 | Intron (ENST00000542672.1/ENSG00000077522.8, intron 1 of 20) | -16.335 | ACTN2 | ENSG00000077522.8 | ENST00000546208.1 |
| 1 | 2.37E+08 | 2.37E+08 | Intron (ENST00000542672.1/ENSG00000077522.8, intron 1 of 20) | -16.334 | ACTN2 | ENSG00000077522.8 | ENST00000546208.1 |
| 1 | 2.38E+08 | 2.38E+08 | Intron (ENST00000450451.1/ENSG00000237250.3, intron 4 of 11) | 6.751 | ZP4 | ENSG00000116996.5 | ENST00000366570.4 |
| 1 | 1.61E+08 | 1.61E+08 | Distal Intergenic | -49 | FCGR2A | ENSG00000143226.9 | ENST00000367972.4 |
| 1 | 1.61E+08 | 1.61E+08 | Distal Intergenic | -39.472 | FCGR2A | ENSG00000143226.9 | ENST00000367972.4 |
| 1 | 2.29E+08 | 2.29E+08 | Promoter (<=1kb) | -0.291 | DUSP5P1 | ENSG00000183929.6 | ENST00000327449.3 |
| 1 | 2.29E+08 | 2.29E+08 | Promoter (<=1kb) | -0.228 | DUSP5P1 | ENSG00000183929.6 | ENST00000327449.3 |
| 1 | 2.29E+08 | 2.29E+08 | Promoter (<=1kb) | -0.203 | DUSP5P1 | ENSG00000183929.6 | ENST00000327449.3 |
| 1 | 2.29E+08 | 2.29E+08 | Promoter (<=1kb) | 0 | DUSP5P1 | ENSG00000183929.6 | ENST00000327449.3 |
| 1 | 2.29E+08 | 2.29E+08 | Promoter (<=1kb) | 0.376 | DUSP5P1 | ENSG00000183929.6 | ENST00000327449.3 |
| 1 | 1.55E+08 | 1.55E+08 | Promoter (<=1kb) | -0.349 | MUC1 | ENSG00000185499.12 | ENST00000462317.1 |
| 1 | 1.55E+08 | 1.55E+08 | Promoter (<=1kb) | -0.41 | MUC1 | ENSG00000185499.12 | ENST00000462317.1 |
| 1 | 1.55E+08 | 1.55E+08 | Promoter (<=1kb) | -0.812 | MUC1 | ENSG00000185499.12 | ENST00000462317.1 |
| 1 | 2.25E+08 | 2.25E+08 | Promoter (1-2kb) | -1.432 | DNAH14 | ENSG00000185842.10 | ENST00000328556.5 |
| 1 | 1.52E+08 | 1.52E+08 | Exon (ENST00000368801.2/ENSG00000197915.5, exon 3 of 3) | 8.968 | HRNR | ENSG00000197915.5 | ENST00000368801.2 |
| 1 | 1.52E+08 | 1.52E+08 | Exon (ENST00000368801.2/ENSG00000197915.5, exon 3 of 3) | 7.132 | HRNR | ENSG00000197915.5 | ENST00000368801.2 |
| 1 | 568943 | 570038 | Promoter (<=1kb) | 0 | RP5-857K21.11 | ENSG00000198744.5 | ENST00000416718.2 |
| 1 | 2.29E+08 | 2.29E+08 | Promoter (<=1kb) | 0.302 | RNA5S12 | ENSG00000199270.1 | ENST00000362400.1 |
| 1 | 2.29E+08 | 2.29E+08 | Promoter (<=1kb) | 0 | RNA5S12 | ENSG00000199270.1 | ENST00000362400.1 |
| 1 | 2.29E+08 | 2.29E+08 | Promoter (<=1kb) | 0.829 | RNA5S11 | ENSG00000199334.1 | ENST00000362464.1 |
| 1 | 2.29E+08 | 2.29E+08 | Promoter (<=1kb) | 0.72 | RNA5S11 | ENSG00000199334.1 | ENST00000362464.1 |
| 1 | 2.29E+08 | 2.29E+08 | Promoter (<=1kb) | 0 | RNA5S11 | ENSG00000199334.1 | ENST00000362464.1 |
| 1 | 2.29E+08 | 2.29E+08 | Promoter (<=1kb) | -0.537 | RNA5S11 | ENSG00000199334.1 | ENST00000362464.1 |
| 1 | 2.29E+08 | 2.29E+08 | Promoter (1-2kb) | -1.039 | RNA5S11 | ENSG00000199334.1 | ENST00000362464.1 |
| 1 | 2.29E+08 | 2.29E+08 | Promoter (<=1kb) | 0.688 | RNA5S3 | ENSG00000199337.1 | ENST00000362467.1 |
| 1 | 2.29E+08 | 2.29E+08 | Promoter (<=1kb) | 0.671 | RNA5S3 | ENSG00000199337.1 | ENST00000362467.1 |
| 1 | 2.29E+08 | 2.29E+08 | Promoter (<=1kb) | 0.187 | RNA5S3 | ENSG00000199337.1 | ENST00000362467.1 |
| 1 | 2.29E+08 | 2.29E+08 | Promoter (<=1kb) | 0 | RNA5S3 | ENSG00000199337.1 | ENST00000362467.1 |
| 1 | 2.29E+08 | 2.29E+08 | Promoter (<=1kb) | -0.364 | RNA5S3 | ENSG00000199337.1 | ENST00000362467.1 |
| 1 | 2.29E+08 | 2.29E+08 | Promoter (<=1kb) | 0.163 | RNA5S1 | ENSG00000199352.1 | ENST00000362482.1 |
| 1 | 2.29E+08 | 2.29E+08 | Promoter (<=1kb) | 0 | RNA5S1 | ENSG00000199352.1 | ENST00000362482.1 |
| 1 | 2.29E+08 | 2.29E+08 | Promoter (<=1kb) | -0.141 | RNA5S1 | ENSG00000199352.1 | ENST00000362482.1 |
| 1 | 2.29E+08 | 2.29E+08 | Promoter (<=1kb) | -0.378 | RNA5S1 | ENSG00000199352.1 | ENST00000362482.1 |
| 1 | 2.29E+08 | 2.29E+08 | Promoter (<=1kb) | 0.548 | RNA5S5 | ENSG00000199396.1 | ENST00000362526.1 |
| 1 | 2.29E+08 | 2.29E+08 | Promoter (<=1kb) | 0.295 | RNA5S5 | ENSG00000199396.1 | ENST00000362526.1 |
| 1 | 2.29E+08 | 2.29E+08 | Promoter (<=1kb) | 0 | RNA5S5 | ENSG00000199396.1 | ENST00000362526.1 |
| 1 | 2.29E+08 | 2.29E+08 | Promoter (<=1kb) | 0 | RNA5S10 | ENSG00000199910.1 | ENST00000363040.1 |
| 1 | 2.29E+08 | 2.29E+08 | Promoter (<=1kb) | -0.159 | RNA5S10 | ENSG00000199910.1 | ENST00000363040.1 |
| 1 | 2.29E+08 | 2.29E+08 | Promoter (<=1kb) | -0.419 | RNA5S10 | ENSG00000199910.1 | ENST00000363040.1 |
| 1 | 2.29E+08 | 2.29E+08 | Promoter (<=1kb) | -0.53 | RNA5S10 | ENSG00000199910.1 | ENST00000363040.1 |
| 1 | 2.29E+08 | 2.29E+08 | Promoter (<=1kb) | -0.645 | RNA5S10 | ENSG00000199910.1 | ENST00000363040.1 |
| 1 | 2.29E+08 | 2.29E+08 | Promoter (<=1kb) | -0.699 | RNA5S10 | ENSG00000199910.1 | ENST00000363040.1 |
| 1 | 2.29E+08 | 2.29E+08 | Promoter (<=1kb) | -0.763 | RNA5S10 | ENSG00000199910.1 | ENST00000363040.1 |
| 1 | 2.29E+08 | 2.29E+08 | Promoter (<=1kb) | 0 | RNA5S8 | ENSG00000200343.1 | ENST00000363473.1 |
| 1 | 2.29E+08 | 2.29E+08 | Promoter (<=1kb) | 0.323 | RNA5S17 | ENSG00000200370.1 | ENST00000363500.1 |
| 1 | 2.29E+08 | 2.29E+08 | Promoter (<=1kb) | 0 | RNA5S4 | ENSG00000200381.1 | ENST00000363511.1 |
| 1 | 2.29E+08 | 2.29E+08 | Promoter (<=1kb) | -0.146 | RNA5S4 | ENSG00000200381.1 | ENST00000363511.1 |
| 1 | 2.29E+08 | 2.29E+08 | Promoter (<=1kb) | -0.22 | RNA5S4 | ENSG00000200381.1 | ENST00000363511.1 |
| 1 | 2.29E+08 | 2.29E+08 | Promoter (<=1kb) | -0.421 | RNA5S4 | ENSG00000200381.1 | ENST00000363511.1 |
| 1 | 2.29E+08 | 2.29E+08 | Promoter (<=1kb) | 0.457 | RNA5S6 | ENSG00000200624.1 | ENST00000363754.1 |
| 1 | 2.29E+08 | 2.29E+08 | Promoter (<=1kb) | 0.368 | RNA5S6 | ENSG00000200624.1 | ENST00000363754.1 |
| 1 | 2.29E+08 | 2.29E+08 | Promoter (<=1kb) | 0 | RNA5S6 | ENSG00000200624.1 | ENST00000363754.1 |
| 1 | 2.29E+08 | 2.29E+08 | Promoter (<=1kb) | -0.025 | RNA5S6 | ENSG00000200624.1 | ENST00000363754.1 |
| 1 | 2.29E+08 | 2.29E+08 | Promoter (<=1kb) | -0.194 | RNA5S6 | ENSG00000200624.1 | ENST00000363754.1 |
| 1 | 2.29E+08 | 2.29E+08 | Promoter (<=1kb) | -0.268 | RNA5S6 | ENSG00000200624.1 | ENST00000363754.1 |
| 1 | 2.29E+08 | 2.29E+08 | Promoter (<=1kb) | -0.563 | RNA5S9 | ENSG00000201321.1 | ENST00000364451.1 |
| 1 | 2.29E+08 | 2.29E+08 | Promoter (<=1kb) | 0.597 | RNA5S14 | ENSG00000201355.1 | ENST00000364485.1 |
| 1 | 2.29E+08 | 2.29E+08 | Promoter (<=1kb) | 0 | RNA5S14 | ENSG00000201355.1 | ENST00000364485.1 |
| 1 | 2.29E+08 | 2.29E+08 | Promoter (<=1kb) | -0.335 | RNA5S14 | ENSG00000201355.1 | ENST00000364485.1 |
| 1 | 2.29E+08 | 2.29E+08 | Promoter (<=1kb) | 0.75 | RNA5S2 | ENSG00000201588.1 | ENST00000364718.1 |
| 1 | 2.29E+08 | 2.29E+08 | Promoter (<=1kb) | 0.71 | RNA5S2 | ENSG00000201588.1 | ENST00000364718.1 |
| 1 | 2.29E+08 | 2.29E+08 | Promoter (<=1kb) | 0 | RNA5S2 | ENSG00000201588.1 | ENST00000364718.1 |
| 1 | 2.29E+08 | 2.29E+08 | Promoter (<=1kb) | -0.338 | RNA5S2 | ENSG00000201588.1 | ENST00000364718.1 |
| 1 | 2.29E+08 | 2.29E+08 | Promoter (<=1kb) | -0.418 | RNA5S2 | ENSG00000201588.1 | ENST00000364718.1 |
| 1 | 2.29E+08 | 2.29E+08 | Promoter (<=1kb) | -0.686 | RNA5S2 | ENSG00000201588.1 | ENST00000364718.1 |
| 1 | 2.29E+08 | 2.29E+08 | Promoter (<=1kb) | -0.701 | RNA5S2 | ENSG00000201588.1 | ENST00000364718.1 |
| 1 | 2.29E+08 | 2.29E+08 | Promoter (<=1kb) | -0.702 | RNA5S2 | ENSG00000201588.1 | ENST00000364718.1 |
| 1 | 2.29E+08 | 2.29E+08 | Promoter (<=1kb) | -0.794 | RNA5S2 | ENSG00000201588.1 | ENST00000364718.1 |
| 1 | 2.29E+08 | 2.29E+08 | Promoter (<=1kb) | 0.559 | RNA5S15 | ENSG00000201925.1 | ENST00000365055.1 |
| 1 | 2.29E+08 | 2.29E+08 | Promoter (<=1kb) | 0.412 | RNA5S15 | ENSG00000201925.1 | ENST00000365055.1 |
| 1 | 2.29E+08 | 2.29E+08 | Promoter (<=1kb) | 0 | RNA5S15 | ENSG00000201925.1 | ENST00000365055.1 |
| 1 | 2.29E+08 | 2.29E+08 | Promoter (<=1kb) | -0.289 | RNA5S15 | ENSG00000201925.1 | ENST00000365055.1 |
| 1 | 2.29E+08 | 2.29E+08 | Promoter (1-2kb) | -1.033 | RNA5S15 | ENSG00000201925.1 | ENST00000365055.1 |
| 1 | 2.29E+08 | 2.29E+08 | Promoter (<=1kb) | 0.545 | RNA5S16 | ENSG00000202257.1 | ENST00000365387.1 |
| 1 | 2.29E+08 | 2.29E+08 | Promoter (<=1kb) | 0.352 | RNA5S16 | ENSG00000202257.1 | ENST00000365387.1 |
| 1 | 2.29E+08 | 2.29E+08 | Promoter (<=1kb) | 0.027 | RNA5S7 | ENSG00000202521.1 | ENST00000365651.1 |
| 1 | 2.29E+08 | 2.29E+08 | Promoter (<=1kb) | -0.121 | RNA5S7 | ENSG00000202521.1 | ENST00000365651.1 |
| 1 | 2.29E+08 | 2.29E+08 | Promoter (1-2kb) | 1.067 | RNA5S13 | ENSG00000202526.1 | ENST00000365656.1 |
| 1 | 2.29E+08 | 2.29E+08 | Promoter (<=1kb) | 0.418 | RNA5S13 | ENSG00000202526.1 | ENST00000365656.1 |
| 1 | 2.29E+08 | 2.29E+08 | Promoter (<=1kb) | 0 | RNA5S13 | ENSG00000202526.1 | ENST00000365656.1 |
| 1 | 2.29E+08 | 2.29E+08 | Promoter (<=1kb) | -0.391 | RNA5S13 | ENSG00000202526.1 | ENST00000365656.1 |
| 1 | 2.29E+08 | 2.29E+08 | Promoter (<=1kb) | -0.752 | RNA5S13 | ENSG00000202526.1 | ENST00000365656.1 |
| 1 | 2.29E+08 | 2.29E+08 | Promoter (1-2kb) | -1.032 | RNA5S13 | ENSG00000202526.1 | ENST00000365656.1 |
| 1 | 2.29E+08 | 2.29E+08 | Promoter (1-2kb) | -1.044 | RNA5S13 | ENSG00000202526.1 | ENST00000365656.1 |
| 1 | 1.49E+08 | 1.49E+08 | Promoter (1-2kb) | 1.197 | RNVU1-18 | ENSG00000206737.1 | ENST00000384010.1 |
| 1 | 1.49E+08 | 1.49E+08 | Promoter (<=1kb) | 0.85 | RNVU1-18 | ENSG00000206737.1 | ENST00000384010.1 |
| 1 | 1.49E+08 | 1.49E+08 | Promoter (<=1kb) | 0.405 | RNVU1-18 | ENSG00000206737.1 | ENST00000384010.1 |
| 1 | 16993273 | 16993999 | Promoter (<=1kb) | 0 | RNU1-3 | ENSG00000207513.1 | ENST00000384782.1 |
| 1 | 16993999 | 16994006 | Promoter (<=1kb) | -0.557 | RNU1-3 | ENSG00000207513.1 | ENST00000384782.1 |
| 1 | 1.61E+08 | 1.61E+08 | Distal Intergenic | 40.237 | RP11-122G18.7 | ENSG00000215840.3 | ENST00000400984.2 |
| 1 | 1.61E+08 | 1.61E+08 | Distal Intergenic | 40.507 | RP11-122G18.7 | ENSG00000215840.3 | ENST00000400984.2 |
| 1 | 1.61E+08 | 1.61E+08 | Distal Intergenic | 40.893 | RP11-122G18.7 | ENSG00000215840.3 | ENST00000400984.2 |
| 1 | 1.61E+08 | 1.61E+08 | Distal Intergenic | 42.021 | RP11-122G18.7 | ENSG00000215840.3 | ENST00000400984.2 |
| 1 | 1.61E+08 | 1.61E+08 | Distal Intergenic | 42.941 | RP11-122G18.7 | ENSG00000215840.3 | ENST00000400984.2 |
| 1 | 1.61E+08 | 1.61E+08 | Distal Intergenic | 43.666 | RP11-122G18.7 | ENSG00000215840.3 | ENST00000400984.2 |
| 1 | 1.61E+08 | 1.61E+08 | Distal Intergenic | 44.387 | RP11-122G18.7 | ENSG00000215840.3 | ENST00000400984.2 |
| 1 | 1.61E+08 | 1.61E+08 | Distal Intergenic | 44.391 | RP11-122G18.7 | ENSG00000215840.3 | ENST00000400984.2 |
| 1 | 1.61E+08 | 1.61E+08 | Distal Intergenic | 46.568 | RP11-122G18.7 | ENSG00000215840.3 | ENST00000400984.2 |
| 1 | 1.61E+08 | 1.61E+08 | Distal Intergenic | 46.643 | RP11-122G18.7 | ENSG00000215840.3 | ENST00000400984.2 |
| 1 | 1.61E+08 | 1.61E+08 | Distal Intergenic | 47.767 | RP11-122G18.7 | ENSG00000215840.3 | ENST00000400984.2 |
| 1 | 1.61E+08 | 1.61E+08 | Distal Intergenic | 49.163 | RP11-122G18.7 | ENSG00000215840.3 | ENST00000400984.2 |
| 1 | 1.61E+08 | 1.61E+08 | Distal Intergenic | 49.178 | RP11-122G18.7 | ENSG00000215840.3 | ENST00000400984.2 |
| 1 | 2583782 | 2584021 | Intron (ENST00000401094.6/ENSG00000215912.7, intron 4 of 8) | -12.632 | TTC34 | ENSG00000215912.7 | ENST00000579787.1 |
| 1 | 2584384 | 2584445 | Intron (ENST00000401094.6/ENSG00000215912.7, intron 4 of 8) | -13.234 | TTC34 | ENSG00000215912.7 | ENST00000579787.1 |
| 1 | 2584445 | 2584454 | Intron (ENST00000401094.6/ENSG00000215912.7, intron 4 of 8) | -13.295 | TTC34 | ENSG00000215912.7 | ENST00000579787.1 |
| 1 | 2584454 | 2584738 | Intron (ENST00000401094.6/ENSG00000215912.7, intron 4 of 8) | -13.304 | TTC34 | ENSG00000215912.7 | ENST00000579787.1 |
| 1 | 2584738 | 2585776 | Intron (ENST00000401094.6/ENSG00000215912.7, intron 4 of 8) | -13.588 | TTC34 | ENSG00000215912.7 | ENST00000579787.1 |
| 1 | 2585776 | 2585787 | Intron (ENST00000401094.6/ENSG00000215912.7, intron 4 of 8) | -14.626 | TTC34 | ENSG00000215912.7 | ENST00000579787.1 |
| 1 | 2585790 | 2585867 | Intron (ENST00000401094.6/ENSG00000215912.7, intron 4 of 8) | -14.64 | TTC34 | ENSG00000215912.7 | ENST00000579787.1 |
| 1 | 2585867 | 2585880 | Intron (ENST00000401094.6/ENSG00000215912.7, intron 4 of 8) | -14.717 | TTC34 | ENSG00000215912.7 | ENST00000579787.1 |
| 1 | 2585880 | 2585904 | Intron (ENST00000401094.6/ENSG00000215912.7, intron 4 of 8) | -14.73 | TTC34 | ENSG00000215912.7 | ENST00000579787.1 |
| 1 | 2585904 | 2585925 | Intron (ENST00000401094.6/ENSG00000215912.7, intron 4 of 8) | -14.754 | TTC34 | ENSG00000215912.7 | ENST00000579787.1 |
| 1 | 2585932 | 2586005 | Intron (ENST00000401094.6/ENSG00000215912.7, intron 4 of 8) | -14.782 | TTC34 | ENSG00000215912.7 | ENST00000579787.1 |
| 1 | 2586005 | 2586049 | Intron (ENST00000401094.6/ENSG00000215912.7, intron 4 of 8) | -14.855 | TTC34 | ENSG00000215912.7 | ENST00000579787.1 |
| 1 | 2586049 | 2586595 | Intron (ENST00000401094.6/ENSG00000215912.7, intron 4 of 8) | -14.899 | TTC34 | ENSG00000215912.7 | ENST00000579787.1 |
| 1 | 2586595 | 2586788 | Intron (ENST00000401094.6/ENSG00000215912.7, intron 4 of 8) | -15.445 | TTC34 | ENSG00000215912.7 | ENST00000579787.1 |
| 1 | 2586788 | 2586884 | Intron (ENST00000401094.6/ENSG00000215912.7, intron 4 of 8) | -15.638 | TTC34 | ENSG00000215912.7 | ENST00000579787.1 |
| 1 | 2586884 | 2586974 | Intron (ENST00000401094.6/ENSG00000215912.7, intron 4 of 8) | -15.734 | TTC34 | ENSG00000215912.7 | ENST00000579787.1 |
| 1 | 2586974 | 2587123 | Intron (ENST00000401094.6/ENSG00000215912.7, intron 4 of 8) | -15.824 | TTC34 | ENSG00000215912.7 | ENST00000579787.1 |
| 1 | 2587123 | 2587360 | Intron (ENST00000401094.6/ENSG00000215912.7, intron 4 of 8) | -15.973 | TTC34 | ENSG00000215912.7 | ENST00000579787.1 |
| 1 | 2587360 | 2587361 | Intron (ENST00000401094.6/ENSG00000215912.7, intron 4 of 8) | -16.21 | TTC34 | ENSG00000215912.7 | ENST00000579787.1 |
| 1 | 2618796 | 2619938 | Intron (ENST00000401094.6/ENSG00000215912.7, intron 4 of 8) | -47.646 | TTC34 | ENSG00000215912.7 | ENST00000579787.1 |
| 1 | 2626898 | 2627718 | Intron (ENST00000401094.6/ENSG00000215912.7, intron 4 of 8) | -55.748 | TTC34 | ENSG00000215912.7 | ENST00000579787.1 |
| 1 | 2628341 | 2628344 | Intron (ENST00000401094.6/ENSG00000215912.7, intron 4 of 8) | -57.191 | TTC34 | ENSG00000215912.7 | ENST00000579787.1 |
| 1 | 2628344 | 2629618 | Intron (ENST00000401094.6/ENSG00000215912.7, intron 4 of 8) | -57.194 | TTC34 | ENSG00000215912.7 | ENST00000579787.1 |
| 1 | 2629618 | 2629739 | Intron (ENST00000401094.6/ENSG00000215912.7, intron 4 of 8) | -58.468 | TTC34 | ENSG00000215912.7 | ENST00000579787.1 |
| 1 | 2629983 | 2630221 | Intron (ENST00000401094.6/ENSG00000215912.7, intron 4 of 8) | -58.833 | TTC34 | ENSG00000215912.7 | ENST00000579787.1 |
| 1 | 16939089 | 16939221 | Promoter (<=1kb) | 0.761 | NBPF1 | ENSG00000219481.6 | ENST00000430580.2 |
| 1 | 16939221 | 16939641 | Promoter (<=1kb) | 0.341 | NBPF1 | ENSG00000219481.6 | ENST00000430580.2 |
| 1 | 16939641 | 16940620 | Promoter (<=1kb) | 0 | NBPF1 | ENSG00000219481.6 | ENST00000430580.2 |
| 1 | 1.21E+08 | 1.21E+08 | Distal Intergenic | -63.576 | RP11-344P13.1 | ENSG00000224857.1 | ENST00000450546.1 |
| 1 | 1.21E+08 | 1.21E+08 | Distal Intergenic | -157.501 | RP11-344P13.1 | ENSG00000224857.1 | ENST00000450546.1 |
| 1 | 1.21E+08 | 1.21E+08 | Distal Intergenic | -160.759 | RP11-344P13.1 | ENSG00000224857.1 | ENST00000450546.1 |
| 1 | 1.21E+08 | 1.21E+08 | Distal Intergenic | -163.319 | RP11-344P13.1 | ENSG00000224857.1 | ENST00000450546.1 |
| 1 | 1.21E+08 | 1.21E+08 | Distal Intergenic | -163.32 | RP11-344P13.1 | ENSG00000224857.1 | ENST00000450546.1 |
| 1 | 1.43E+08 | 1.43E+08 | Distal Intergenic | -16.674 | RP11-417J8.1 | ENSG00000227552.1 | ENST00000445662.1 |
| 1 | 1.43E+08 | 1.43E+08 | Distal Intergenic | -14.142 | RP11-417J8.1 | ENSG00000227552.1 | ENST00000445662.1 |
| 1 | 567175 | 568111 | Promoter (<=1kb) | -0.026 | RP5-857K21.7 | ENSG00000229344.1 | ENST00000427426.1 |
| 1 | 1.43E+08 | 1.43E+08 | Distal Intergenic | 5.828 | RP11-423O2.3 | ENSG00000230446.1 | ENST00000444462.1 |
| 1 | 1.43E+08 | 1.43E+08 | Distal Intergenic | 65.467 | RP11-423O2.7 | ENSG00000231182.2 | ENST00000424640.2 |
| 1 | 2.49E+08 | 2.49E+08 | Distal Intergenic | 8.755 | AL672183.2 | ENSG00000233084.2 | ENST00000430973.1 |
| 1 | 2.49E+08 | 2.49E+08 | Distal Intergenic | 8.976 | AL672183.2 | ENSG00000233084.2 | ENST00000430973.1 |
| 1 | 2.49E+08 | 2.49E+08 | Distal Intergenic | 8.977 | AL672183.2 | ENSG00000233084.2 | ENST00000430973.1 |
| 1 | 2.49E+08 | 2.49E+08 | Distal Intergenic | 9.91 | AL672183.2 | ENSG00000233084.2 | ENST00000430973.1 |
| 1 | 2.49E+08 | 2.49E+08 | Distal Intergenic | 9.947 | AL672183.2 | ENSG00000233084.2 | ENST00000430973.1 |
| 1 | 1.43E+08 | 1.43E+08 | Distal Intergenic | 34.376 | RP11-782C8.8 | ENSG00000234654.1 | ENST00000445742.1 |
| 1 | 1.43E+08 | 1.43E+08 | Distal Intergenic | 34.862 | RP11-782C8.8 | ENSG00000234654.1 | ENST00000445742.1 |
| 1 | 1.43E+08 | 1.43E+08 | Distal Intergenic | 34.902 | RP11-782C8.8 | ENSG00000234654.1 | ENST00000445742.1 |
| 1 | 1.43E+08 | 1.43E+08 | Distal Intergenic | 37.184 | RP11-782C8.8 | ENSG00000234654.1 | ENST00000445742.1 |
| 1 | 1.43E+08 | 1.43E+08 | Distal Intergenic | 38.428 | RP11-782C8.8 | ENSG00000234654.1 | ENST00000445742.1 |
| 1 | 2.36E+08 | 2.36E+08 | Distal Intergenic | -13.088 | RP4-764D2.1 | ENSG00000235371.1 | ENST00000446607.1 |
| 1 | 2.36E+08 | 2.36E+08 | Distal Intergenic | -12.558 | RP4-764D2.1 | ENSG00000235371.1 | ENST00000446607.1 |
| 1 | 2.36E+08 | 2.36E+08 | Distal Intergenic | -12.547 | RP4-764D2.1 | ENSG00000235371.1 | ENST00000446607.1 |
| 1 | 1.52E+08 | 1.52E+08 | Exon (ENST00000368801.2/ENSG00000197915.5, exon 3 of 3) | 7.858 | RP11-107M16.2 | ENSG00000236427.1 | ENST00000429352.1 |
| 1 | 1.52E+08 | 1.52E+08 | Exon (ENST00000368801.2/ENSG00000197915.5, exon 3 of 3) | 7.962 | RP11-107M16.2 | ENSG00000236427.1 | ENST00000429352.1 |
| 1 | 568610 | 568890 | Promoter (<=1kb) | -0.025 | MTATP8P1 | ENSG00000240409.1 | ENST00000467115.1 |
| 1 | 34712900 | 34712906 | Distal Intergenic | 29.987 | RP4-657M3.2 | ENSG00000270241.1 | ENST00000604298.1 |
| 1 | 34712906 | 34713361 | Distal Intergenic | 29.532 | RP4-657M3.2 | ENSG00000270241.1 | ENST00000604298.1 |
| 1 | 34713361 | 34713391 | Distal Intergenic | 29.502 | RP4-657M3.2 | ENSG00000270241.1 | ENST00000604298.1 |
| 2 | 45642781 | 45643109 | Intron (ENST00000263736.4/ENSG00000068784.8, intron 18 of 20) | 32.125 | SRBD1 | ENSG00000068784.8 | ENST00000490133.1 |
| 2 | 91815082 | 91815889 | Intron (ENST00000609777.1/ENSG00000143429.5, intron 2 of 2) | 27.515 | AC027612.6 | ENSG00000143429.5 | ENST00000608018.1 |
| 2 | 88480665 | 88480935 | Promoter (1-2kb) | -1.139 | THNSL2 | ENSG00000144115.12 | ENST00000464022.1 |
| 2 | 1.14E+08 | 1.14E+08 | Promoter (<=1kb) | 0 | WASH2P | ENSG00000146556.10 | ENST00000326632.7 |
| 2 | 10262154 | 10262938 | Promoter (<=1kb) | 0 | RRM2 | ENSG00000171848.9 | ENST00000360566.2 |
| 2 | 10262938 | 10263067 | Promoter (<=1kb) | 0.06 | RRM2 | ENSG00000171848.9 | ENST00000491447.1 |
| 2 | 1217420 | 1217536 | Intron (ENST00000308624.5/ENSG00000172554.7, intron 9 of 16) | 12.606 | SNTG2 | ENSG00000172554.7 | ENST00000498321.1 |
| 2 | 1217536 | 1217550 | Intron (ENST00000308624.5/ENSG00000172554.7, intron 9 of 16) | 12.722 | SNTG2 | ENSG00000172554.7 | ENST00000498321.1 |
| 2 | 1217550 | 1218687 | Intron (ENST00000308624.5/ENSG00000172554.7, intron 9 of 16) | 12.736 | SNTG2 | ENSG00000172554.7 | ENST00000498321.1 |
| 2 | 1218687 | 1218701 | Intron (ENST00000308624.5/ENSG00000172554.7, intron 9 of 16) | 13.873 | SNTG2 | ENSG00000172554.7 | ENST00000498321.1 |
| 2 | 1218701 | 1218727 | Intron (ENST00000308624.5/ENSG00000172554.7, intron 9 of 16) | 13.887 | SNTG2 | ENSG00000172554.7 | ENST00000498321.1 |
| 2 | 1.55E+08 | 1.55E+08 | Distal Intergenic | -13.456 | RNA5SP107 | ENSG00000223290.1 | ENST00000411358.1 |
| 2 | 1.55E+08 | 1.55E+08 | Distal Intergenic | -12.908 | RNA5SP107 | ENSG00000223290.1 | ENST00000411358.1 |
| 2 | 1.55E+08 | 1.55E+08 | Distal Intergenic | -12.855 | RNA5SP107 | ENSG00000223290.1 | ENST00000411358.1 |
| 2 | 92268284 | 92268299 | Distal Intergenic | -45.259 | AC128677.4 | ENSG00000223816.4 | ENST00000451163.2 |
| 2 | 92268299 | 92268537 | Distal Intergenic | -45.274 | AC128677.4 | ENSG00000223816.4 | ENST00000451163.2 |
| 2 | 92268537 | 92268539 | Distal Intergenic | -45.512 | AC128677.4 | ENSG00000223816.4 | ENST00000451163.2 |
| 2 | 92274342 | 92274790 | Distal Intergenic | -51.317 | AC128677.4 | ENSG00000223816.4 | ENST00000451163.2 |
| 2 | 92285698 | 92285757 | Distal Intergenic | -62.673 | AC128677.4 | ENSG00000223816.4 | ENST00000451163.2 |
| 2 | 92285757 | 92286558 | Distal Intergenic | -62.732 | AC128677.4 | ENSG00000223816.4 | ENST00000451163.2 |
| 2 | 92286558 | 92286652 | Distal Intergenic | -63.533 | AC128677.4 | ENSG00000223816.4 | ENST00000451163.2 |
| 2 | 92303106 | 92303716 | Distal Intergenic | -80.081 | AC128677.4 | ENSG00000223816.4 | ENST00000451163.2 |
| 2 | 87623132 | 87624294 | Distal Intergenic | -15.243 | AC068279.3 | ENSG00000224881.1 | ENST00000444323.1 |
| 2 | 87641498 | 87643031 | Distal Intergenic | -33.609 | AC068279.3 | ENSG00000224881.1 | ENST00000444323.1 |
| 2 | 2299052 | 2299094 | Intron (ENST00000399161.2/ENSG00000186487.13, intron 1 of 24) | -23.91 | AC009232.2 | ENSG00000225619.1 | ENST00000422175.1 |
| 2 | 2299094 | 2299133 | Intron (ENST00000399161.2/ENSG00000186487.13, intron 1 of 24) | -23.871 | AC009232.2 | ENSG00000225619.1 | ENST00000422175.1 |
| 2 | 2299133 | 2299741 | Intron (ENST00000399161.2/ENSG00000186487.13, intron 1 of 24) | -23.263 | AC009232.2 | ENSG00000225619.1 | ENST00000422175.1 |
| 2 | 1.62E+08 | 1.62E+08 | Promoter (<=1kb) | 0 | AC009299.4 | ENSG00000225813.1 | ENST00000429684.1 |
| 2 | 1.62E+08 | 1.62E+08 | Promoter (<=1kb) | 0.821 | AC009299.4 | ENSG00000225813.1 | ENST00000429684.1 |
| 2 | 91601743 | 91602234 | Distal Intergenic | -33.697 | AC018696.1 | ENSG00000226615.1 | ENST00000428749.1 |
| 2 | 1.1E+08 | 1.1E+08 | Intron (ENST00000418513.1/ENSG00000172985.8, intron 1 of 7) | -52.215 | SNRPGP9 | ENSG00000228551.1 | ENST00000456025.1 |
| 2 | 1.1E+08 | 1.1E+08 | Intron (ENST00000418513.1/ENSG00000172985.8, intron 1 of 7) | -51.51 | SNRPGP9 | ENSG00000228551.1 | ENST00000456025.1 |
| 2 | 1.1E+08 | 1.1E+08 | Intron (ENST00000418513.1/ENSG00000172985.8, intron 1 of 7) | -51.459 | SNRPGP9 | ENSG00000228551.1 | ENST00000456025.1 |
| 2 | 1.33E+08 | 1.33E+08 | Intron (ENST00000440802.1/ENSG00000230803.1, intron 2 of 2) | 8.076 | AC097532.2 | ENSG00000230803.1 | ENST00000440802.1 |
| 2 | 1.33E+08 | 1.33E+08 | Intron (ENST00000440802.1/ENSG00000230803.1, intron 2 of 2) | 7.097 | AC097532.2 | ENSG00000230803.1 | ENST00000440802.1 |
| 2 | 91761944 | 91762644 | Distal Intergenic | -3.893 | AC018696.4 | ENSG00000230964.1 | ENST00000454518.1 |
| 2 | 91762925 | 91763742 | Promoter (2-3kb) | -2.795 | AC018696.4 | ENSG00000230964.1 | ENST00000454518.1 |
| 2 | 91793277 | 91794149 | Intron (ENST00000443031.1/ENSG00000233991.2, intron 2 of 4) | 15.742 | AC116050.1 | ENSG00000233991.2 | ENST00000443031.1 |
| 2 | 91807833 | 91808156 | Intron (ENST00000443031.1/ENSG00000233991.2, intron 4 of 4) | 30.298 | AC116050.1 | ENSG00000233991.2 | ENST00000443031.1 |
| 2 | 1.99E+08 | 1.99E+08 | Distal Intergenic | 36.82 | AC005235.1 | ENSG00000236653.1 | ENST00000451266.1 |
| 2 | 89851990 | 89852593 | Distal Intergenic | -38.008 | IGKV2D-40 | ENSG00000251039.2 | ENST00000429992.2 |
| 2 | 89852673 | 89853724 | Distal Intergenic | -36.877 | IGKV2D-40 | ENSG00000251039.2 | ENST00000429992.2 |
| 2 | 89874575 | 89876158 | Distal Intergenic | -14.443 | IGKV2D-40 | ENSG00000251039.2 | ENST00000429992.2 |
| 2 | 89878322 | 89880130 | Distal Intergenic | -10.471 | IGKV2D-40 | ENSG00000251039.2 | ENST00000429992.2 |
| 2 | 90382855 | 90382886 | Distal Intergenic | -75.315 | CH17-132F21.1 | ENSG00000270999.1 | ENST00000603238.1 |
| 2 | 90382886 | 90384685 | Distal Intergenic | -73.516 | CH17-132F21.1 | ENSG00000270999.1 | ENST00000603238.1 |
| 2 | 90384685 | 90384717 | Distal Intergenic | -73.484 | CH17-132F21.1 | ENSG00000270999.1 | ENST00000603238.1 |
| 2 | 90481504 | 90481552 | Distal Intergenic | 23.304 | CH17-132F21.1 | ENSG00000270999.1 | ENST00000603238.1 |
| 2 | 90481552 | 90483395 | Distal Intergenic | 23.352 | CH17-132F21.1 | ENSG00000270999.1 | ENST00000603238.1 |
| 3 | 73159786 | 73159817 | Promoter (<=1kb) | -0.326 | RNU2-64P | ENSG00000223247.1 | ENST00000411315.1 |
| 3 | 73159817 | 73160419 | Promoter (<=1kb) | 0 | RNU2-64P | ENSG00000223247.1 | ENST00000411315.1 |
| 3 | 73160419 | 73160436 | Promoter (<=1kb) | 0.277 | RNU2-64P | ENSG00000223247.1 | ENST00000411315.1 |
| 3 | 75717971 | 75719264 | Promoter (<=1kb) | 0 | RP11-413E6.7 | ENSG00000236138.4 | ENST00000489078.1 |
| 3 | 1.95E+08 | 1.95E+08 | Intron (ENST00000432194.3/ENSG00000242086.4, intron 3 of 3) | 4.149 | LINC00969 | ENSG00000242086.4 | ENST00000432601.1 |
| 3 | 90447855 | 90448584 | Distal Intergenic | -136.998 | RP11-557B13.1 | ENSG00000271024.1 | ENST00000603327.1 |
| 4 | 49098081 | 49098133 | Distal Intergenic | 67.373 | CWH43 | ENSG00000109182.7 | ENST00000507372.1 |
| 4 | 49098133 | 49098550 | Distal Intergenic | 67.425 | CWH43 | ENSG00000109182.7 | ENST00000507372.1 |
| 4 | 49098550 | 49098807 | Distal Intergenic | 67.842 | CWH43 | ENSG00000109182.7 | ENST00000507372.1 |
| 4 | 49099675 | 49101147 | Distal Intergenic | 68.967 | CWH43 | ENSG00000109182.7 | ENST00000507372.1 |
| 4 | 49101404 | 49102015 | Distal Intergenic | 70.696 | CWH43 | ENSG00000109182.7 | ENST00000507372.1 |
| 4 | 49109301 | 49109308 | Distal Intergenic | 78.593 | CWH43 | ENSG00000109182.7 | ENST00000507372.1 |
| 4 | 49109308 | 49109776 | Distal Intergenic | 78.6 | CWH43 | ENSG00000109182.7 | ENST00000507372.1 |
| 4 | 49109776 | 49109781 | Distal Intergenic | 79.068 | CWH43 | ENSG00000109182.7 | ENST00000507372.1 |
| 4 | 49109830 | 49109846 | Distal Intergenic | 79.122 | CWH43 | ENSG00000109182.7 | ENST00000507372.1 |
| 4 | 49109846 | 49109873 | Distal Intergenic | 79.138 | CWH43 | ENSG00000109182.7 | ENST00000507372.1 |
| 4 | 49109873 | 49111624 | Distal Intergenic | 79.165 | CWH43 | ENSG00000109182.7 | ENST00000507372.1 |
| 4 | 49111624 | 49111696 | Distal Intergenic | 80.916 | CWH43 | ENSG00000109182.7 | ENST00000507372.1 |
| 4 | 49111696 | 49111744 | Distal Intergenic | 80.988 | CWH43 | ENSG00000109182.7 | ENST00000507372.1 |
| 4 | 49111793 | 49111804 | Distal Intergenic | 81.085 | CWH43 | ENSG00000109182.7 | ENST00000507372.1 |
| 4 | 49111804 | 49112174 | Distal Intergenic | 81.096 | CWH43 | ENSG00000109182.7 | ENST00000507372.1 |
| 4 | 49112174 | 49112318 | Distal Intergenic | 81.466 | CWH43 | ENSG00000109182.7 | ENST00000507372.1 |
| 4 | 49112466 | 49112496 | Distal Intergenic | 81.758 | CWH43 | ENSG00000109182.7 | ENST00000507372.1 |
| 4 | 49112496 | 49112762 | Distal Intergenic | 81.788 | CWH43 | ENSG00000109182.7 | ENST00000507372.1 |
| 4 | 49112762 | 49112929 | Distal Intergenic | 82.054 | CWH43 | ENSG00000109182.7 | ENST00000507372.1 |
| 4 | 7865543 | 7865556 | Promoter (1-2kb) | -1.506 | AFAP1 | ENSG00000196526.6 | ENST00000513856.1 |
| 4 | 7865556 | 7866019 | Promoter (1-2kb) | -1.519 | AFAP1 | ENSG00000196526.6 | ENST00000513856.1 |
| 4 | 49136259 | 49137170 | Distal Intergenic | -62.05 | AC118282.3 | ENSG00000222437.1 | ENST00000410505.1 |
| 4 | 49137856 | 49138367 | Distal Intergenic | -60.853 | AC118282.3 | ENSG00000222437.1 | ENST00000410505.1 |
| 4 | 49139799 | 49140425 | Distal Intergenic | -58.795 | AC118282.3 | ENSG00000222437.1 | ENST00000410505.1 |
| 4 | 49145808 | 49147071 | Distal Intergenic | -52.149 | AC118282.3 | ENSG00000222437.1 | ENST00000410505.1 |
| 4 | 49150889 | 49150896 | Distal Intergenic | -48.324 | AC118282.3 | ENSG00000222437.1 | ENST00000410505.1 |
| 4 | 49150896 | 49150901 | Distal Intergenic | -48.319 | AC118282.3 | ENSG00000222437.1 | ENST00000410505.1 |
| 4 | 49150901 | 49152244 | Distal Intergenic | -46.976 | AC118282.3 | ENSG00000222437.1 | ENST00000410505.1 |
| 4 | 49152255 | 49152850 | Distal Intergenic | -46.37 | AC118282.3 | ENSG00000222437.1 | ENST00000410505.1 |
| 4 | 49152850 | 49152866 | Distal Intergenic | -46.354 | AC118282.3 | ENSG00000222437.1 | ENST00000410505.1 |
| 4 | 49152866 | 49152885 | Distal Intergenic | -46.335 | AC118282.3 | ENSG00000222437.1 | ENST00000410505.1 |
| 4 | 49153019 | 49153293 | Distal Intergenic | -45.927 | AC118282.3 | ENSG00000222437.1 | ENST00000410505.1 |
| 4 | 49154965 | 49155979 | Distal Intergenic | -43.241 | AC118282.3 | ENSG00000222437.1 | ENST00000410505.1 |
| 4 | 49155979 | 49155980 | Distal Intergenic | -43.24 | AC118282.3 | ENSG00000222437.1 | ENST00000410505.1 |
| 4 | 49157325 | 49157705 | Distal Intergenic | -41.515 | AC118282.3 | ENSG00000222437.1 | ENST00000410505.1 |
| 4 | 49157912 | 49158643 | Distal Intergenic | -40.577 | AC118282.3 | ENSG00000222437.1 | ENST00000410505.1 |
| 4 | 49635830 | 49635862 | Distal Intergenic | -34.305 | AC119751.4 | ENSG00000223099.1 | ENST00000411167.1 |
| 4 | 49635862 | 49635884 | Distal Intergenic | -34.337 | AC119751.4 | ENSG00000223099.1 | ENST00000411167.1 |
| 4 | 49635884 | 49637779 | Distal Intergenic | -34.359 | AC119751.4 | ENSG00000223099.1 | ENST00000411167.1 |
| 4 | 49637779 | 49637782 | Distal Intergenic | -36.254 | AC119751.4 | ENSG00000223099.1 | ENST00000411167.1 |
| 4 | 49638818 | 49638914 | Distal Intergenic | -37.293 | AC119751.4 | ENSG00000223099.1 | ENST00000411167.1 |
| 4 | 49638914 | 49640465 | Distal Intergenic | -37.389 | AC119751.4 | ENSG00000223099.1 | ENST00000411167.1 |
| 4 | 49640465 | 49640551 | Distal Intergenic | -38.94 | AC119751.4 | ENSG00000223099.1 | ENST00000411167.1 |
| 4 | 49640596 | 49640633 | Distal Intergenic | -39.071 | AC119751.4 | ENSG00000223099.1 | ENST00000411167.1 |
| 4 | 49640902 | 49641449 | Distal Intergenic | -39.377 | AC119751.4 | ENSG00000223099.1 | ENST00000411167.1 |
| 4 | 49641449 | 49641471 | Distal Intergenic | -39.924 | AC119751.4 | ENSG00000223099.1 | ENST00000411167.1 |
| 4 | 49642083 | 49643697 | Distal Intergenic | -40.558 | AC119751.4 | ENSG00000223099.1 | ENST00000411167.1 |
| 4 | 49643925 | 49643970 | Distal Intergenic | -42.4 | AC119751.4 | ENSG00000223099.1 | ENST00000411167.1 |
| 4 | 49643970 | 49644227 | Distal Intergenic | -42.445 | AC119751.4 | ENSG00000223099.1 | ENST00000411167.1 |
| 4 | 49644227 | 49644401 | Distal Intergenic | -42.702 | AC119751.4 | ENSG00000223099.1 | ENST00000411167.1 |
| 4 | 49644401 | 49645149 | Distal Intergenic | -42.876 | AC119751.4 | ENSG00000223099.1 | ENST00000411167.1 |
| 4 | 49645149 | 49645155 | Distal Intergenic | -43.624 | AC119751.4 | ENSG00000223099.1 | ENST00000411167.1 |
| 4 | 49650493 | 49651283 | Distal Intergenic | -48.968 | AC119751.4 | ENSG00000223099.1 | ENST00000411167.1 |
| 4 | 1.91E+08 | 1.91E+08 | Promoter (<=1kb) | 0 | DUX4L9 | ENSG00000224807.5 | ENST00000449051.3 |
| 4 | 1.91E+08 | 1.91E+08 | Promoter (<=1kb) | -0.142 | DUX4L9 | ENSG00000224807.5 | ENST00000449051.3 |
| 4 | 49272350 | 49273803 | Distal Intergenic | 23.733 | RP11-1281K21.7 | ENSG00000248946.2 | ENST00000510932.2 |
| 4 | 49292800 | 49294594 | Distal Intergenic | 44.183 | RP11-1281K21.7 | ENSG00000248946.2 | ENST00000510932.2 |
| 4 | 49305313 | 49305413 | Distal Intergenic | 56.696 | RP11-1281K21.7 | ENSG00000248946.2 | ENST00000510932.2 |
| 4 | 49305413 | 49306159 | Distal Intergenic | 56.796 | RP11-1281K21.7 | ENSG00000248946.2 | ENST00000510932.2 |
| 4 | 49306159 | 49306234 | Distal Intergenic | 57.542 | RP11-1281K21.7 | ENSG00000248946.2 | ENST00000510932.2 |
| 4 | 49324667 | 49324897 | Distal Intergenic | 76.05 | RP11-1281K21.7 | ENSG00000248946.2 | ENST00000510932.2 |
| 4 | 49324897 | 49325850 | Distal Intergenic | 76.28 | RP11-1281K21.7 | ENSG00000248946.2 | ENST00000510932.2 |
| 4 | 49325850 | 49325873 | Distal Intergenic | 77.233 | RP11-1281K21.7 | ENSG00000248946.2 | ENST00000510932.2 |
| 4 | 40297255 | 40297909 | Distal Intergenic | -20.593 | AC195454.1 | ENSG00000249241.1 | ENST00000510551.1 |
| 4 | 1.9E+08 | 1.9E+08 | Distal Intergenic | 13.989 | RP11-706F1.2 | ENSG00000249877.1 | ENST00000504057.1 |
| 4 | 1.9E+08 | 1.9E+08 | Distal Intergenic | 13.486 | RP11-706F1.2 | ENSG00000249877.1 | ENST00000504057.1 |
| 4 | 1.9E+08 | 1.9E+08 | Distal Intergenic | 13.471 | RP11-706F1.2 | ENSG00000249877.1 | ENST00000504057.1 |
| 4 | 49514814 | 49514837 | Distal Intergenic | -3.992 | RP11-241F15.3 | ENSG00000250769.2 | ENST00000514077.2 |
| 4 | 49514837 | 49514908 | Distal Intergenic | -4.015 | RP11-241F15.3 | ENSG00000250769.2 | ENST00000514077.2 |
| 4 | 49514908 | 49515057 | Distal Intergenic | -4.086 | RP11-241F15.3 | ENSG00000250769.2 | ENST00000514077.2 |
| 4 | 49515057 | 49515112 | Distal Intergenic | -4.235 | RP11-241F15.3 | ENSG00000250769.2 | ENST00000514077.2 |
| 4 | 49515112 | 49515199 | Distal Intergenic | -4.29 | RP11-241F15.3 | ENSG00000250769.2 | ENST00000514077.2 |
| 4 | 49515199 | 49515996 | Distal Intergenic | -4.377 | RP11-241F15.3 | ENSG00000250769.2 | ENST00000514077.2 |
| 4 | 49515996 | 49516074 | Distal Intergenic | -5.174 | RP11-241F15.3 | ENSG00000250769.2 | ENST00000514077.2 |
| 4 | 49516091 | 49516131 | Distal Intergenic | -5.269 | RP11-241F15.3 | ENSG00000250769.2 | ENST00000514077.2 |
| 4 | 49516131 | 49516335 | Distal Intergenic | -5.309 | RP11-241F15.3 | ENSG00000250769.2 | ENST00000514077.2 |
| 4 | 49516335 | 49516345 | Distal Intergenic | -5.513 | RP11-241F15.3 | ENSG00000250769.2 | ENST00000514077.2 |
| 4 | 49516345 | 49516351 | Distal Intergenic | -5.523 | RP11-241F15.3 | ENSG00000250769.2 | ENST00000514077.2 |
| 4 | 49516413 | 49516422 | Distal Intergenic | -5.591 | RP11-241F15.3 | ENSG00000250769.2 | ENST00000514077.2 |
| 4 | 49516422 | 49516432 | Distal Intergenic | -5.6 | RP11-241F15.3 | ENSG00000250769.2 | ENST00000514077.2 |
| 4 | 49516432 | 49517117 | Distal Intergenic | -5.61 | RP11-241F15.3 | ENSG00000250769.2 | ENST00000514077.2 |
| 4 | 49517117 | 49517127 | Distal Intergenic | -6.295 | RP11-241F15.3 | ENSG00000250769.2 | ENST00000514077.2 |
| 4 | 49517127 | 49517138 | Distal Intergenic | -6.305 | RP11-241F15.3 | ENSG00000250769.2 | ENST00000514077.2 |
| 4 | 49517138 | 49517146 | Distal Intergenic | -6.316 | RP11-241F15.3 | ENSG00000250769.2 | ENST00000514077.2 |
| 5 | 1025713 | 1026420 | Intron (ENST00000296849.5/ENSG00000145506.9, intron 3 of 9) | -7.831 | NKD2 | ENSG00000145506.9 | ENST00000519933.1 |
| 5 | 49405593 | 49406118 | Distal Intergenic | 301.714 | EMB | ENSG00000170571.7 | ENST00000505896.1 |
| 5 | 49431283 | 49432390 | Distal Intergenic | 275.442 | EMB | ENSG00000170571.7 | ENST00000505896.1 |
| 5 | 49438230 | 49438654 | Distal Intergenic | 269.178 | EMB | ENSG00000170571.7 | ENST00000505896.1 |
| 5 | 49438654 | 49438926 | Distal Intergenic | 268.906 | EMB | ENSG00000170571.7 | ENST00000505896.1 |
| 5 | 34189687 | 34190340 | Promoter (<=1kb) | 0 | RP11-1023L17.2 | ENSG00000215156.5 | ENST00000505001.2 |
| 5 | 34190340 | 34191107 | Promoter (<=1kb) | 0.18 | RP11-1023L17.2 | ENSG00000215156.5 | ENST00000505001.2 |
| 5 | 34191310 | 34191392 | Promoter (1-2kb) | 1.15 | RP11-1023L17.2 | ENSG00000215156.5 | ENST00000505001.2 |
| 5 | 34191548 | 34192022 | Promoter (1-2kb) | 1.388 | RP11-1023L17.2 | ENSG00000215156.5 | ENST00000505001.2 |
| 5 | 34192171 | 34192206 | Promoter (2-3kb) | 2.011 | RP11-1023L17.2 | ENSG00000215156.5 | ENST00000505001.2 |
| 5 | 34192206 | 34192511 | Promoter (2-3kb) | 2.046 | RP11-1023L17.2 | ENSG00000215156.5 | ENST00000505001.2 |
| 5 | 34192794 | 34193458 | Promoter (2-3kb) | 2.634 | RP11-1023L17.2 | ENSG00000215156.5 | ENST00000505001.2 |
| 5 | 34193458 | 34193566 | Exon (ENST00000505001.2/ENSG00000215156.5, exon 1 of 1) | 3.298 | RP11-1023L17.2 | ENSG00000215156.5 | ENST00000505001.2 |
| 5 | 34179773 | 34180867 | Promoter (2-3kb) | 2.103 | RP11-1023L17.1 | ENSG00000215158.5 | ENST00000380721.4 |
| 5 | 17597084 | 17597126 | Promoter (<=1kb) | 0.811 | RP11-432M8.5 | ENSG00000249329.1 | ENST00000503077.1 |
| 5 | 17597126 | 17598148 | Promoter (<=1kb) | 0 | RP11-432M8.5 | ENSG00000249329.1 | ENST00000503077.1 |
| 5 | 17598148 | 17598203 | Promoter (<=1kb) | -0.212 | RP11-432M8.5 | ENSG00000249329.1 | ENST00000503077.1 |
| 5 | 17598203 | 17598233 | Promoter (<=1kb) | -0.267 | RP11-432M8.5 | ENSG00000249329.1 | ENST00000503077.1 |
| 5 | 17521005 | 17522234 | Promoter (<=1kb) | 0 | RP11-321E2.7 | ENSG00000249339.1 | ENST00000512357.1 |
| 5 | 9915 | 11897 | Distal Intergenic | -46.416 | RP11-811I15.1 | ENSG00000250020.1 | ENST00000506872.1 |
| 5 | 11897 | 11899 | Distal Intergenic | -46.414 | RP11-811I15.1 | ENSG00000250020.1 | ENST00000506872.1 |
| 5 | 11899 | 11905 | Distal Intergenic | -46.408 | RP11-811I15.1 | ENSG00000250020.1 | ENST00000506872.1 |
| 5 | 11905 | 11912 | Distal Intergenic | -46.401 | RP11-811I15.1 | ENSG00000250020.1 | ENST00000506872.1 |
| 5 | 11912 | 11913 | Distal Intergenic | -46.4 | RP11-811I15.1 | ENSG00000250020.1 | ENST00000506872.1 |
| 5 | 17525175 | 17526846 | Promoter (<=1kb) | 0 | RP11-321E2.8 | ENSG00000250055.1 | ENST00000510009.1 |
| 5 | 17590151 | 17590555 | Promoter (<=1kb) | 0.514 | RP11-432M8.3 | ENSG00000250558.1 | ENST00000505742.1 |
| 5 | 415359 | 415377 | Intron (ENST00000505113.1/ENSG00000063438.12, intron 5 of 10) | -3.441 | CTD-2228K2.1 | ENSG00000250645.1 | ENST00000509301.1 |
| 5 | 415377 | 415812 | Intron (ENST00000505113.1/ENSG00000063438.12, intron 5 of 10) | -3.006 | CTD-2228K2.1 | ENSG00000250645.1 | ENST00000509301.1 |
| 5 | 17583750 | 17583986 | Promoter (1-2kb) | 1.881 | RP11-432M8.2 | ENSG00000250807.2 | ENST00000506051.1 |
| 5 | 17584938 | 17585851 | Promoter (<=1kb) | 0.016 | RP11-432M8.2 | ENSG00000250807.2 | ENST00000506051.1 |
| 6 | 1.58E+08 | 1.58E+08 | Intron (ENST00000400788.4/ENSG00000215712.6, intron 3 of 3) | 10.284 | TMEM242 | ENSG00000215712.6 | ENST00000400788.4 |
| 6 | 1.58E+08 | 1.58E+08 | Intron (ENST00000400788.4/ENSG00000215712.6, intron 3 of 3) | -10.284 | LDHAL6FP | ENSG00000217783.2 | ENST00000405916.2 |
| 6 | 1.58E+08 | 1.58E+08 | Intron (ENST00000400788.4/ENSG00000215712.6, intron 3 of 3) | -11.05 | LDHAL6FP | ENSG00000217783.2 | ENST00000405916.2 |
| 6 | 1.58E+08 | 1.58E+08 | Intron (ENST00000400788.4/ENSG00000215712.6, intron 3 of 3) | -11.397 | LDHAL6FP | ENSG00000217783.2 | ENST00000405916.2 |
| 6 | 1.58E+08 | 1.58E+08 | Intron (ENST00000400788.4/ENSG00000215712.6, intron 3 of 3) | -11.646 | LDHAL6FP | ENSG00000217783.2 | ENST00000405916.2 |
| 6 | 468812 | 469655 | Distal Intergenic | -54.516 | RP1-20B11.2 | ENSG00000230433.1 | ENST00000430842.1 |
| 6 | 469655 | 469659 | Distal Intergenic | -54.512 | RP1-20B11.2 | ENSG00000230433.1 | ENST00000430842.1 |
| 6 | 469659 | 469674 | Distal Intergenic | -54.497 | RP1-20B11.2 | ENSG00000230433.1 | ENST00000430842.1 |
| 6 | 1.7E+08 | 1.7E+08 | Distal Intergenic | -4.911 | RP11-302L19.3 | ENSG00000273100.1 | ENST00000610240.1 |
| 6 | 1.7E+08 | 1.7E+08 | Distal Intergenic | -4.899 | RP11-302L19.3 | ENSG00000273100.1 | ENST00000610240.1 |
| 6 | 1.7E+08 | 1.7E+08 | Distal Intergenic | -4.251 | RP11-302L19.3 | ENSG00000273100.1 | ENST00000610240.1 |
| 6 | 1.7E+08 | 1.7E+08 | Distal Intergenic | -4.244 | RP11-302L19.3 | ENSG00000273100.1 | ENST00000610240.1 |
| 7 | 1.59E+08 | 1.59E+08 | Intron (ENST00000407559.3/ENSG00000126870.11, intron 14 of 24) | -18.971 | WDR60 | ENSG00000126870.11 | ENST00000454771.1 |
| 7 | 1.59E+08 | 1.59E+08 | Intron (ENST00000407559.3/ENSG00000126870.11, intron 14 of 24) | -18.248 | WDR60 | ENSG00000126870.11 | ENST00000454771.1 |
| 7 | 71262896 | 71263294 | Intron (ENST00000329008.5/ENSG00000183166.6, intron 5 of 5) | -112.165 | RN7SKP75 | ENSG00000199940.1 | ENST00000363070.1 |
| 7 | 1.01E+08 | 1.01E+08 | Exon (ENST00000379442.3/ENSG00000205277.5, exon 5 of 15) | -13.831 | MUC12 | ENSG00000205277.5 | ENST00000305119.3 |
| 7 | 1.01E+08 | 1.01E+08 | Exon (ENST00000379442.3/ENSG00000205277.5, exon 5 of 15) | -13.446 | MUC12 | ENSG00000205277.5 | ENST00000305119.3 |
| 7 | 1.01E+08 | 1.01E+08 | Exon (ENST00000379442.3/ENSG00000205277.5, exon 5 of 15) | -13.221 | MUC12 | ENSG00000205277.5 | ENST00000305119.3 |
| 7 | 1.01E+08 | 1.01E+08 | Exon (ENST00000379442.3/ENSG00000205277.5, exon 5 of 15) | -12.369 | MUC12 | ENSG00000205277.5 | ENST00000305119.3 |
| 7 | 1.41E+08 | 1.41E+08 | Intron (ENST00000488785.1/ENSG00000228775.3, intron 2 of 6) | 3.56 | RNU1-82P | ENSG00000212153.1 | ENST00000390851.1 |
| 7 | 1.41E+08 | 1.41E+08 | Intron (ENST00000488785.1/ENSG00000228775.3, intron 2 of 6) | 4.073 | RNU1-82P | ENSG00000212153.1 | ENST00000390851.1 |
| 7 | 56439008 | 56440478 | Distal Intergenic | -8.191 | RP11-814E24.3 | ENSG00000224370.1 | ENST00000450682.1 |
| 7 | 56440478 | 56440593 | Distal Intergenic | -8.076 | RP11-814E24.3 | ENSG00000224370.1 | ENST00000450682.1 |
| 7 | 56440988 | 56441041 | Distal Intergenic | -7.628 | RP11-814E24.3 | ENSG00000224370.1 | ENST00000450682.1 |
| 7 | 56441041 | 56441761 | Distal Intergenic | -6.908 | RP11-814E24.3 | ENSG00000224370.1 | ENST00000450682.1 |
| 7 | 56441761 | 56441810 | Distal Intergenic | -6.859 | RP11-814E24.3 | ENSG00000224370.1 | ENST00000450682.1 |
| 7 | 56438346 | 56438378 | Distal Intergenic | 8.196 | CICP8 | ENSG00000225371.1 | ENST00000455800.1 |
| 7 | 56438378 | 56438808 | Distal Intergenic | 8.228 | CICP8 | ENSG00000225371.1 | ENST00000455800.1 |
| 7 | 56438896 | 56438914 | Distal Intergenic | 8.746 | CICP8 | ENSG00000225371.1 | ENST00000455800.1 |
| 7 | 56438914 | 56439008 | Distal Intergenic | 8.764 | CICP8 | ENSG00000225371.1 | ENST00000455800.1 |
| 7 | 1.58E+08 | 1.58E+08 | Intron (ENST00000389413.3/ENSG00000155093.13, intron 6 of 21) | 119.751 | AC011899.10 | ENSG00000231980.1 | ENST00000436489.1 |
| 7 | 9876 | 10267 | Distal Intergenic | 61.568 | AC093627.7 | ENSG00000232325.3 | ENST00000465755.1 |
| 7 | 23910 | 23983 | Distal Intergenic | 47.852 | AC093627.7 | ENSG00000232325.3 | ENST00000465755.1 |
| 7 | 23983 | 24501 | Distal Intergenic | 47.334 | AC093627.7 | ENSG00000232325.3 | ENST00000465755.1 |
| 7 | 24501 | 24508 | Distal Intergenic | 47.327 | AC093627.7 | ENSG00000232325.3 | ENST00000465755.1 |
| 7 | 61054961 | 61055937 | Distal Intergenic | -765.932 | RP11-715L17.1 | ENSG00000233918.1 | ENST00000454392.1 |
| 7 | 61785602 | 61786643 | Distal Intergenic | -35.226 | RP11-715L17.1 | ENSG00000233918.1 | ENST00000454392.1 |
| 7 | 61794035 | 61794081 | Distal Intergenic | -27.788 | RP11-715L17.1 | ENSG00000233918.1 | ENST00000454392.1 |
| 7 | 61794081 | 61794930 | Distal Intergenic | -26.939 | RP11-715L17.1 | ENSG00000233918.1 | ENST00000454392.1 |
| 7 | 61825021 | 61826122 | Distal Intergenic | 3.153 | RP11-715L17.1 | ENSG00000233918.1 | ENST00000454392.1 |
| 7 | 61971888 | 61973303 | Distal Intergenic | 150.02 | RP11-715L17.1 | ENSG00000233918.1 | ENST00000454392.1 |
| 7 | 61975820 | 61976732 | Distal Intergenic | 153.952 | RP11-715L17.1 | ENSG00000233918.1 | ENST00000454392.1 |
| 7 | 1.57E+08 | 1.57E+08 | Distal Intergenic | -5.216 | AC006372.4 | ENSG00000234210.1 | ENST00000444158.1 |
| 7 | 1.57E+08 | 1.57E+08 | Distal Intergenic | -5.202 | AC006372.4 | ENSG00000234210.1 | ENST00000444158.1 |
| 7 | 1.57E+08 | 1.57E+08 | Distal Intergenic | -4.381 | AC006372.4 | ENSG00000234210.1 | ENST00000444158.1 |
| 7 | 1.57E+08 | 1.57E+08 | Distal Intergenic | -4.366 | AC006372.4 | ENSG00000234210.1 | ENST00000444158.1 |
| 7 | 1.57E+08 | 1.57E+08 | Distal Intergenic | -4.358 | AC006372.4 | ENSG00000234210.1 | ENST00000444158.1 |
| 7 | 1.57E+08 | 1.57E+08 | Distal Intergenic | -4.353 | AC006372.4 | ENSG00000234210.1 | ENST00000444158.1 |
| 8 | 68888544 | 68889089 | Intron (ENST00000288368.4/ENSG00000046889.14, intron 1 of 39) | 23.821 | PREX2 | ENSG00000046889.14 | ENST00000517617.1 |
| 8 | 82754444 | 82754969 | Promoter (<=1kb) | 0 | SNX16 | ENSG00000104497.9 | ENST00000396330.2 |
| 8 | 22253746 | 22253765 | Promoter (1-2kb) | -1.976 | SLC39A14 | ENSG00000104635.9 | ENST00000519960.1 |
| 8 | 22253765 | 22253793 | Promoter (1-2kb) | -1.948 | SLC39A14 | ENSG00000104635.9 | ENST00000519960.1 |
| 8 | 22253793 | 22254336 | Promoter (1-2kb) | -1.405 | SLC39A14 | ENSG00000104635.9 | ENST00000519960.1 |
| 8 | 22254336 | 22254338 | Promoter (1-2kb) | -1.403 | SLC39A14 | ENSG00000104635.9 | ENST00000519960.1 |
| 8 | 22254338 | 22254355 | Promoter (1-2kb) | -1.386 | SLC39A14 | ENSG00000104635.9 | ENST00000519960.1 |
| 8 | 1.42E+08 | 1.42E+08 | Distal Intergenic | 10.88 | DENND3 | ENSG00000105339.6 | ENST00000520725.1 |
| 8 | 1.44E+08 | 1.44E+08 | Promoter (<=1kb) | -0.382 | C8orf31 | ENSG00000177335.6 | ENST00000517653.1 |
| 8 | 86574584 | 86575081 | Promoter (<=1kb) | 0.645 | REXO1L1 | ENSG00000205176.2 | ENST00000379010.2 |
| 8 | 86575171 | 86575245 | Promoter (<=1kb) | 0.481 | REXO1L1 | ENSG00000205176.2 | ENST00000379010.2 |
| 8 | 86575607 | 86576226 | Promoter (<=1kb) | 0 | REXO1L1 | ENSG00000205176.2 | ENST00000379010.2 |
| 8 | 86747042 | 86748280 | Promoter (<=1kb) | 0 | REXO1L11P | ENSG00000223524.2 | ENST00000425429.2 |
| 8 | 86748477 | 86750090 | Promoter (<=1kb) | 0.935 | REXO1L11P | ENSG00000223524.2 | ENST00000425429.2 |
| 8 | 1.43E+08 | 1.43E+08 | Intron (ENST00000521053.1/ENSG00000226807.2, intron 3 of 27) | 4.516 | MROH5 | ENSG00000226807.2 | ENST00000521161.1 |
| 8 | 1.43E+08 | 1.43E+08 | Intron (ENST00000521053.1/ENSG00000226807.2, intron 3 of 27) | 4.035 | MROH5 | ENSG00000226807.2 | ENST00000521161.1 |
| 8 | 1.43E+08 | 1.43E+08 | Intron (ENST00000521053.1/ENSG00000226807.2, intron 3 of 27) | 3.543 | MROH5 | ENSG00000226807.2 | ENST00000521161.1 |
| 8 | 1.45E+08 | 1.45E+08 | Exon (ENST00000525985.1/ENSG00000227184.3, exon 2 of 2) | 11.765 | EPPK1 | ENSG00000227184.3 | ENST00000525985.1 |
| 8 | 43834727 | 43835093 | Distal Intergenic | -304.994 | RP11-643N23.1 | ENSG00000253198.1 | ENST00000519417.1 |
| 8 | 43835093 | 43835799 | Distal Intergenic | -305.36 | RP11-643N23.1 | ENSG00000253198.1 | ENST00000519417.1 |
| 8 | 43835799 | 43835810 | Distal Intergenic | -306.066 | RP11-643N23.1 | ENSG00000253198.1 | ENST00000519417.1 |
| 8 | 43835810 | 43835835 | Distal Intergenic | -306.077 | RP11-643N23.1 | ENSG00000253198.1 | ENST00000519417.1 |
| 8 | 58118200 | 58118258 | Intron (ENST00000519241.1/ENSG00000253301.1, intron 3 of 3) | 12.022 | RP11-513O17.2 | ENSG00000253301.1 | ENST00000523341.1 |
| 8 | 58118258 | 58118974 | Intron (ENST00000519241.1/ENSG00000253301.1, intron 3 of 3) | -11.861 | RP11-513O17.2 | ENSG00000253301.1 | ENST00000520929.1 |
| 8 | 58118974 | 58119084 | Intron (ENST00000519241.1/ENSG00000253301.1, intron 3 of 3) | -11.751 | RP11-513O17.2 | ENSG00000253301.1 | ENST00000520929.1 |
| 8 | 58119165 | 58119189 | Intron (ENST00000519241.1/ENSG00000253301.1, intron 3 of 3) | -11.646 | RP11-513O17.2 | ENSG00000253301.1 | ENST00000520929.1 |
| 8 | 58122060 | 58123118 | Intron (ENST00000519241.1/ENSG00000253301.1, intron 3 of 3) | -7.717 | RP11-513O17.2 | ENSG00000253301.1 | ENST00000520929.1 |
| 8 | 43092557 | 43092618 | Distal Intergenic | 9.46 | RP11-726G23.2 | ENSG00000253884.1 | ENST00000518796.1 |
| 8 | 43092618 | 43093369 | Distal Intergenic | 8.709 | RP11-726G23.2 | ENSG00000253884.1 | ENST00000518796.1 |
| 8 | 43093369 | 43093467 | Distal Intergenic | 8.611 | RP11-726G23.2 | ENSG00000253884.1 | ENST00000518796.1 |
| 8 | 43094479 | 43094569 | Distal Intergenic | 7.509 | RP11-726G23.2 | ENSG00000253884.1 | ENST00000518796.1 |
| 8 | 43094569 | 43095390 | Distal Intergenic | 6.688 | RP11-726G23.2 | ENSG00000253884.1 | ENST00000518796.1 |
| 8 | 43095390 | 43095540 | Distal Intergenic | 6.538 | RP11-726G23.2 | ENSG00000253884.1 | ENST00000518796.1 |
| 8 | 43096526 | 43097184 | Distal Intergenic | 4.894 | RP11-726G23.2 | ENSG00000253884.1 | ENST00000518796.1 |
| 8 | 9113798 | 9113813 | Intron (ENST00000523246.1/ENSG00000254235.1, intron 1 of 2) | 3.992 | RP11-10A14.7 | ENSG00000253887.1 | ENST00000518589.1 |
| 8 | 9113813 | 9114241 | Intron (ENST00000523246.1/ENSG00000254235.1, intron 1 of 2) | 3.564 | RP11-10A14.7 | ENSG00000253887.1 | ENST00000518589.1 |
| 8 | 9114241 | 9114257 | Intron (ENST00000523246.1/ENSG00000254235.1, intron 1 of 2) | 3.548 | RP11-10A14.7 | ENSG00000253887.1 | ENST00000518589.1 |
| 8 | 46841342 | 46842821 | Distal Intergenic | -329.361 | AC113134.1 | ENSG00000255915.1 | ENST00000539774.1 |
| 8 | 46842821 | 46842834 | Distal Intergenic | -329.348 | AC113134.1 | ENSG00000255915.1 | ENST00000539774.1 |
| 8 | 46848156 | 46848817 | Distal Intergenic | -323.365 | AC113134.1 | ENSG00000255915.1 | ENST00000539774.1 |
| 8 | 86766284 | 86767068 | Distal Intergenic | -7.54 | REXO1L10P | ENSG00000255940.1 | ENST00000540724.1 |
| 8 | 86814506 | 86814949 | Distal Intergenic | 25.928 | REXO1L2P | ENSG00000270375.1 | ENST00000605109.1 |
| 8 | 86814949 | 86815993 | Distal Intergenic | 24.884 | REXO1L2P | ENSG00000270375.1 | ENST00000605109.1 |
| 8 | 86816127 | 86816416 | Distal Intergenic | 24.461 | REXO1L2P | ENSG00000270375.1 | ENST00000605109.1 |
| 8 | 86816416 | 86816429 | Distal Intergenic | 24.448 | REXO1L2P | ENSG00000270375.1 | ENST00000605109.1 |
| 8 | 86838696 | 86839282 | Promoter (1-2kb) | 1.595 | REXO1L2P | ENSG00000270375.1 | ENST00000605109.1 |
| 8 | 86839316 | 86840645 | Promoter (<=1kb) | 0.232 | REXO1L2P | ENSG00000270375.1 | ENST00000605109.1 |
| 8 | 86555099 | 86556231 | Promoter (<=1kb) | 0.47 | REXO1L8P | ENSG00000270971.1 | ENST00000604378.1 |
| 8 | 86557514 | 86557524 | Promoter (<=1kb) | -0.814 | REXO1L8P | ENSG00000270971.1 | ENST00000604378.1 |
| 8 | 86557524 | 86558083 | Promoter (<=1kb) | -0.824 | REXO1L8P | ENSG00000270971.1 | ENST00000604378.1 |
| 8 | 86558083 | 86558854 | Promoter (1-2kb) | -1.383 | REXO1L8P | ENSG00000270971.1 | ENST00000604378.1 |
| 8 | 86786902 | 86787358 | Distal Intergenic | -10.067 | REXO1L9P | ENSG00000271381.1 | ENST00000604264.1 |
| 8 | 86787358 | 86787414 | Distal Intergenic | -10.523 | REXO1L9P | ENSG00000271381.1 | ENST00000604264.1 |
| 8 | 86787414 | 86788032 | Distal Intergenic | -10.579 | REXO1L9P | ENSG00000271381.1 | ENST00000604264.1 |
| 8 | 86802041 | 86803593 | Distal Intergenic | -25.206 | REXO1L9P | ENSG00000271381.1 | ENST00000604264.1 |
| 8 | 86806117 | 86807544 | Distal Intergenic | -29.282 | REXO1L9P | ENSG00000271381.1 | ENST00000604264.1 |
| 9 | 44992142 | 44992177 | Promoter (1-2kb) | 1.829 | FAM27C | ENSG00000154537.4 | ENST00000377542.3 |
| 9 | 44992177 | 44992249 | Promoter (1-2kb) | 1.864 | FAM27C | ENSG00000154537.4 | ENST00000377542.3 |
| 9 | 44992249 | 44993511 | Promoter (1-2kb) | 1.936 | FAM27C | ENSG00000154537.4 | ENST00000377542.3 |
| 9 | 44993511 | 44993540 | Distal Intergenic | 3.198 | FAM27C | ENSG00000154537.4 | ENST00000377542.3 |
| 9 | 28354996 | 28355063 | Intron (ENST00000379992.2/ENSG00000174482.6, intron 3 of 5) | -59.668 | LINGO2 | ENSG00000174482.6 | ENST00000493941.1 |
| 9 | 28355063 | 28355071 | Intron (ENST00000379992.2/ENSG00000174482.6, intron 3 of 5) | -59.735 | LINGO2 | ENSG00000174482.6 | ENST00000493941.1 |
| 9 | 28355071 | 28355513 | Intron (ENST00000379992.2/ENSG00000174482.6, intron 3 of 5) | -59.743 | LINGO2 | ENSG00000174482.6 | ENST00000493941.1 |
| 9 | 28355513 | 28355514 | Intron (ENST00000379992.2/ENSG00000174482.6, intron 3 of 5) | -60.185 | LINGO2 | ENSG00000174482.6 | ENST00000493941.1 |
| 9 | 28355514 | 28355547 | Intron (ENST00000379992.2/ENSG00000174482.6, intron 3 of 5) | -60.186 | LINGO2 | ENSG00000174482.6 | ENST00000493941.1 |
| 9 | 1.4E+08 | 1.4E+08 | Intron (ENST00000491734.2/ENSG00000187609.11, intron 11 of 14) | 24.519 | EXD3 | ENSG00000187609.11 | ENST00000487745.1 |
| 9 | 1.4E+08 | 1.4E+08 | Intron (ENST00000491734.2/ENSG00000187609.11, intron 11 of 14) | 24.408 | EXD3 | ENSG00000187609.11 | ENST00000487745.1 |
| 9 | 1.36E+08 | 1.36E+08 | Intron (ENST00000354484.4/ENSG00000197859.5, intron 12 of 18) | 16.575 | FAM163B | ENSG00000196990.4 | ENST00000356873.3 |
| 9 | 1.36E+08 | 1.36E+08 | Intron (ENST00000354484.4/ENSG00000197859.5, intron 12 of 18) | 15.371 | FAM163B | ENSG00000196990.4 | ENST00000356873.3 |
| 9 | 66457897 | 66458619 | Promoter (<=1kb) | 0 | RNA5SP283 | ENSG00000202474.1 | ENST00000365604.1 |
| 9 | 66458619 | 66458685 | Promoter (<=1kb) | -0.358 | RNA5SP283 | ENSG00000202474.1 | ENST00000365604.1 |
| 9 | 66458685 | 66458731 | Promoter (<=1kb) | -0.424 | RNA5SP283 | ENSG00000202474.1 | ENST00000365604.1 |
| 9 | 66460781 | 66460826 | Promoter (2-3kb) | -2.52 | RNA5SP283 | ENSG00000202474.1 | ENST00000365604.1 |
| 9 | 66460826 | 66461130 | Promoter (2-3kb) | -2.565 | RNA5SP283 | ENSG00000202474.1 | ENST00000365604.1 |
| 9 | 66461130 | 66461812 | Promoter (2-3kb) | -2.869 | RNA5SP283 | ENSG00000202474.1 | ENST00000365604.1 |
| 9 | 66461812 | 66461855 | Intron (ENST00000427509.1/ENSG00000238113.2, intron 2 of 2) | -3.551 | RNA5SP283 | ENSG00000202474.1 | ENST00000365604.1 |
| 9 | 66461855 | 66461924 | Intron (ENST00000427509.1/ENSG00000238113.2, intron 2 of 2) | -3.594 | RNA5SP283 | ENSG00000202474.1 | ENST00000365604.1 |
| 9 | 66461924 | 66461987 | Intron (ENST00000427509.1/ENSG00000238113.2, intron 2 of 2) | -3.663 | RNA5SP283 | ENSG00000202474.1 | ENST00000365604.1 |
| 9 | 1.41E+08 | 1.41E+08 | Intron (ENST00000462942.1/ENSG00000181090.13, intron 20 of 21) | 6.573 | MIR602 | ENSG00000207693.1 | ENST00000384960.1 |
| 9 | 1.41E+08 | 1.41E+08 | Intron (ENST00000462942.1/ENSG00000181090.13, intron 20 of 21) | 6.601 | MIR602 | ENSG00000207693.1 | ENST00000384960.1 |
| 9 | 1.41E+08 | 1.41E+08 | Intron (ENST00000462942.1/ENSG00000181090.13, intron 20 of 21) | 7.825 | MIR602 | ENSG00000207693.1 | ENST00000384960.1 |
| 9 | 87879607 | 87880258 | Distal Intergenic | 105.011 | UBE2V1P10 | ENSG00000226590.1 | ENST00000414685.1 |
| 9 | 68412433 | 68412461 | Promoter (1-2kb) | 1.735 | LINC00537 | ENSG00000232815.1 | ENST00000455245.1 |
| 9 | 68412461 | 68412792 | Promoter (1-2kb) | 1.404 | LINC00537 | ENSG00000232815.1 | ENST00000455245.1 |
| 9 | 68412792 | 68412813 | Promoter (1-2kb) | 1.383 | LINC00537 | ENSG00000232815.1 | ENST00000455245.1 |
| 9 | 68412877 | 68412882 | Promoter (1-2kb) | 1.314 | LINC00537 | ENSG00000232815.1 | ENST00000455245.1 |
| 9 | 68412882 | 68412936 | Promoter (1-2kb) | 1.26 | LINC00537 | ENSG00000232815.1 | ENST00000455245.1 |
| 9 | 68412936 | 68413100 | Promoter (1-2kb) | 1.096 | LINC00537 | ENSG00000232815.1 | ENST00000455245.1 |
| 9 | 68413253 | 68414457 | Promoter (<=1kb) | 0 | LINC00537 | ENSG00000232815.1 | ENST00000455245.1 |
| 9 | 68414457 | 68414567 | Promoter (<=1kb) | -0.262 | LINC00537 | ENSG00000232815.1 | ENST00000455245.1 |
| 9 | 1.41E+08 | 1.41E+08 | Intron (ENST00000540522.1/ENSG00000233013.4, intron 5 of 6) | 14.373 | FAM157B | ENSG00000233013.4 | ENST00000446912.2 |
| 9 | 1.41E+08 | 1.41E+08 | Intron (ENST00000540522.1/ENSG00000233013.4, intron 5 of 6) | 15.312 | FAM157B | ENSG00000233013.4 | ENST00000446912.2 |
| 9 | 1.41E+08 | 1.41E+08 | Intron (ENST00000540522.1/ENSG00000233013.4, intron 5 of 6) | 15.396 | FAM157B | ENSG00000233013.4 | ENST00000446912.2 |
| 9 | 1.41E+08 | 1.41E+08 | Intron (ENST00000540522.1/ENSG00000233013.4, intron 5 of 6) | 15.532 | FAM157B | ENSG00000233013.4 | ENST00000446912.2 |
| 9 | 1.41E+08 | 1.41E+08 | Intron (ENST00000540522.1/ENSG00000233013.4, intron 5 of 6) | 16.243 | FAM157B | ENSG00000233013.4 | ENST00000446912.2 |
| 9 | 67790749 | 67790774 | Promoter (1-2kb) | 1.076 | RP11-12A20.6 | ENSG00000233172.2 | ENST00000419793.2 |
| 9 | 67790774 | 67790852 | Promoter (1-2kb) | 1.101 | RP11-12A20.6 | ENSG00000233172.2 | ENST00000419793.2 |
| 9 | 69048112 | 69048175 | Distal Intergenic | -17.48 | RP11-87H9.2 | ENSG00000233961.1 | ENST00000424707.1 |
| 9 | 69048175 | 69048210 | Distal Intergenic | -17.445 | RP11-87H9.2 | ENSG00000233961.1 | ENST00000424707.1 |
| 9 | 69048210 | 69049120 | Distal Intergenic | -16.535 | RP11-87H9.2 | ENSG00000233961.1 | ENST00000424707.1 |
| 9 | 45352004 | 45352017 | Distal Intergenic | -11.464 | CDK2AP2P1 | ENSG00000234299.4 | ENST00000492018.2 |
| 9 | 45352017 | 45352933 | Distal Intergenic | -10.548 | CDK2AP2P1 | ENSG00000234299.4 | ENST00000492018.2 |
| 9 | 45352933 | 45352966 | Distal Intergenic | -10.515 | CDK2AP2P1 | ENSG00000234299.4 | ENST00000492018.2 |
| 9 | 45352966 | 45353020 | Distal Intergenic | -10.461 | CDK2AP2P1 | ENSG00000234299.4 | ENST00000492018.2 |
| 9 | 45355656 | 45357465 | Distal Intergenic | -6.016 | CDK2AP2P1 | ENSG00000234299.4 | ENST00000492018.2 |
| 9 | 67790852 | 67792174 | Promoter (<=1kb) | 0 | RP11-12A20.7 | ENSG00000236233.3 | ENST00000315762.5 |
| 9 | 67792174 | 67792200 | Promoter (<=1kb) | 0.008 | RP11-12A20.7 | ENSG00000236233.3 | ENST00000315762.5 |
| 9 | 67792200 | 67792238 | Promoter (<=1kb) | 0.034 | RP11-12A20.7 | ENSG00000236233.3 | ENST00000315762.5 |
| 9 | 66456777 | 66457217 | Promoter (<=1kb) | -0.068 | RP11-262H14.1 | ENSG00000238113.2 | ENST00000427509.1 |
| 9 | 66457217 | 66457534 | Promoter (<=1kb) | 0 | RP11-262H14.1 | ENSG00000238113.2 | ENST00000427509.1 |
| 9 | 66457725 | 66457731 | Promoter (<=1kb) | -0.169 | RP11-262H14.1 | ENSG00000238113.2 | ENST00000452184.2 |
| 9 | 66457731 | 66457881 | Promoter (<=1kb) | -0.019 | RP11-262H14.1 | ENSG00000238113.2 | ENST00000452184.2 |
| 9 | 66457881 | 66457897 | Promoter (<=1kb) | -0.003 | RP11-262H14.1 | ENSG00000238113.2 | ENST00000452184.2 |
| 9 | 68425212 | 68425247 | Distal Intergenic | 9.905 | MIR4477B | ENSG00000266017.1 | ENST00000581659.1 |
| 9 | 66834576 | 66834599 | Promoter (2-3kb) | -2.063 | RP11-318K12.3 | ENSG00000270092.2 | ENST00000602407.2 |
| 9 | 66834599 | 66835246 | Promoter (1-2kb) | -1.416 | RP11-318K12.3 | ENSG00000270092.2 | ENST00000602407.2 |
| 9 | 66835246 | 66835248 | Promoter (1-2kb) | -1.414 | RP11-318K12.3 | ENSG00000270092.2 | ENST00000602407.2 |
| 10 | 6509093 | 6509116 | Intron (ENST00000263125.5/ENSG00000065675.10, intron 12 of 17) | 113.085 | PRKCQ | ENSG00000065675.10 | ENST00000539722.1 |
| 10 | 6509116 | 6509151 | Intron (ENST00000263125.5/ENSG00000065675.10, intron 12 of 17) | 113.05 | PRKCQ | ENSG00000065675.10 | ENST00000539722.1 |
| 10 | 6509151 | 6509559 | Intron (ENST00000263125.5/ENSG00000065675.10, intron 12 of 17) | 112.642 | PRKCQ | ENSG00000065675.10 | ENST00000539722.1 |
| 10 | 6509559 | 6509582 | Intron (ENST00000263125.5/ENSG00000065675.10, intron 12 of 17) | 112.619 | PRKCQ | ENSG00000065675.10 | ENST00000539722.1 |
| 10 | 1.27E+08 | 1.27E+08 | Intron (ENST00000337195.5/ENSG00000175029.12, intron 2 of 10) | 39.773 | CTBP2 | ENSG00000175029.12 | ENST00000530930.1 |
| 10 | 1.27E+08 | 1.27E+08 | Intron (ENST00000337195.5/ENSG00000175029.12, intron 2 of 10) | 39.271 | CTBP2 | ENSG00000175029.12 | ENST00000530930.1 |
| 10 | 1.27E+08 | 1.27E+08 | Intron (ENST00000337195.5/ENSG00000175029.12, intron 2 of 10) | 39.214 | CTBP2 | ENSG00000175029.12 | ENST00000530930.1 |
| 10 | 1.35E+08 | 1.35E+08 | Intron (ENST00000607359.1/ENSG00000197177.11, intron 14 of 15) | 9.055 | GPR123 | ENSG00000197177.11 | ENST00000392606.2 |
| 10 | 1.35E+08 | 1.35E+08 | Intron (ENST00000607359.1/ENSG00000197177.11, intron 14 of 15) | 9.069 | GPR123 | ENSG00000197177.11 | ENST00000392606.2 |
| 10 | 1.35E+08 | 1.35E+08 | Intron (ENST00000607359.1/ENSG00000197177.11, intron 14 of 15) | 9.539 | GPR123 | ENSG00000197177.11 | ENST00000392606.2 |
| 10 | 42355357 | 42355362 | Distal Intergenic | 289.635 | KSR1P1 | ENSG00000229485.1 | ENST00000446298.1 |
| 10 | 42355362 | 42355641 | Distal Intergenic | 289.356 | KSR1P1 | ENSG00000229485.1 | ENST00000446298.1 |
| 10 | 42358898 | 42359837 | Distal Intergenic | 285.16 | KSR1P1 | ENSG00000229485.1 | ENST00000446298.1 |
| 10 | 42359915 | 42359922 | Distal Intergenic | 285.075 | KSR1P1 | ENSG00000229485.1 | ENST00000446298.1 |
| 10 | 42359922 | 42359991 | Distal Intergenic | 285.006 | KSR1P1 | ENSG00000229485.1 | ENST00000446298.1 |
| 10 | 42359991 | 42360001 | Distal Intergenic | 284.996 | KSR1P1 | ENSG00000229485.1 | ENST00000446298.1 |
| 10 | 42360165 | 42360360 | Distal Intergenic | 284.637 | KSR1P1 | ENSG00000229485.1 | ENST00000446298.1 |
| 10 | 42360360 | 42361401 | Distal Intergenic | 283.596 | KSR1P1 | ENSG00000229485.1 | ENST00000446298.1 |
| 10 | 42361401 | 42361418 | Distal Intergenic | 283.579 | KSR1P1 | ENSG00000229485.1 | ENST00000446298.1 |
| 10 | 42361539 | 42361619 | Distal Intergenic | 283.378 | KSR1P1 | ENSG00000229485.1 | ENST00000446298.1 |
| 10 | 42362698 | 42362977 | Distal Intergenic | 282.02 | KSR1P1 | ENSG00000229485.1 | ENST00000446298.1 |
| 10 | 42363117 | 42363809 | Distal Intergenic | 281.188 | KSR1P1 | ENSG00000229485.1 | ENST00000446298.1 |
| 10 | 42364450 | 42365132 | Distal Intergenic | 279.865 | KSR1P1 | ENSG00000229485.1 | ENST00000446298.1 |
| 10 | 42367596 | 42368260 | Distal Intergenic | 276.737 | KSR1P1 | ENSG00000229485.1 | ENST00000446298.1 |
| 10 | 42368260 | 42368265 | Distal Intergenic | 276.732 | KSR1P1 | ENSG00000229485.1 | ENST00000446298.1 |
| 10 | 42368310 | 42368630 | Distal Intergenic | 276.367 | KSR1P1 | ENSG00000229485.1 | ENST00000446298.1 |
| 10 | 42368774 | 42369517 | Distal Intergenic | 275.48 | KSR1P1 | ENSG00000229485.1 | ENST00000446298.1 |
| 10 | 42379781 | 42381169 | Distal Intergenic | 263.828 | KSR1P1 | ENSG00000229485.1 | ENST00000446298.1 |
| 10 | 42401750 | 42401772 | Distal Intergenic | 243.225 | KSR1P1 | ENSG00000229485.1 | ENST00000446298.1 |
| 10 | 42401772 | 42402574 | Distal Intergenic | 242.423 | KSR1P1 | ENSG00000229485.1 | ENST00000446298.1 |
| 10 | 42402574 | 42402598 | Distal Intergenic | 242.399 | KSR1P1 | ENSG00000229485.1 | ENST00000446298.1 |
| 10 | 42409016 | 42409549 | Distal Intergenic | 235.448 | KSR1P1 | ENSG00000229485.1 | ENST00000446298.1 |
| 10 | 42527160 | 42528605 | Distal Intergenic | 116.392 | KSR1P1 | ENSG00000229485.1 | ENST00000446298.1 |
| 10 | 42536320 | 42536337 | Distal Intergenic | 108.66 | KSR1P1 | ENSG00000229485.1 | ENST00000446298.1 |
| 10 | 42536337 | 42537747 | Distal Intergenic | 107.25 | KSR1P1 | ENSG00000229485.1 | ENST00000446298.1 |
| 10 | 42538402 | 42538475 | Distal Intergenic | 106.522 | KSR1P1 | ENSG00000229485.1 | ENST00000446298.1 |
| 10 | 42538475 | 42539234 | Distal Intergenic | 105.763 | KSR1P1 | ENSG00000229485.1 | ENST00000446298.1 |
| 10 | 42539234 | 42539266 | Distal Intergenic | 105.731 | KSR1P1 | ENSG00000229485.1 | ENST00000446298.1 |
| 10 | 42539324 | 42539352 | Distal Intergenic | 105.645 | KSR1P1 | ENSG00000229485.1 | ENST00000446298.1 |
| 10 | 42539352 | 42540794 | Distal Intergenic | 104.203 | KSR1P1 | ENSG00000229485.1 | ENST00000446298.1 |
| 10 | 42541256 | 42541283 | Distal Intergenic | 103.714 | KSR1P1 | ENSG00000229485.1 | ENST00000446298.1 |
| 10 | 42541283 | 42542185 | Distal Intergenic | 102.812 | KSR1P1 | ENSG00000229485.1 | ENST00000446298.1 |
| 10 | 42542185 | 42542215 | Distal Intergenic | 102.782 | KSR1P1 | ENSG00000229485.1 | ENST00000446298.1 |
| 10 | 42545650 | 42546401 | Distal Intergenic | 98.596 | KSR1P1 | ENSG00000229485.1 | ENST00000446298.1 |
| 10 | 39125317 | 39125344 | Distal Intergenic | 49.183 | RP11-96F8.1 | ENSG00000233832.1 | ENST00000427864.1 |
| 10 | 39125344 | 39125860 | Distal Intergenic | 49.21 | RP11-96F8.1 | ENSG00000233832.1 | ENST00000427864.1 |
| 10 | 39125860 | 39125903 | Distal Intergenic | 49.726 | RP11-96F8.1 | ENSG00000233832.1 | ENST00000427864.1 |
| 10 | 1.3E+08 | 1.3E+08 | Distal Intergenic | -144.029 | RP11-264E18.1 | ENSG00000234640.1 | ENST00000454492.1 |
| 10 | 1.3E+08 | 1.3E+08 | Distal Intergenic | -144.068 | RP11-264E18.1 | ENSG00000234640.1 | ENST00000454492.1 |
| 10 | 1.3E+08 | 1.3E+08 | Distal Intergenic | -144.521 | RP11-264E18.1 | ENSG00000234640.1 | ENST00000454492.1 |
| 10 | 1.32E+08 | 1.32E+08 | Distal Intergenic | -78.187 | RP11-540N6.1 | ENSG00000236303.2 | ENST00000439421.2 |
| 11 | 1.03E+08 | 1.03E+08 | Distal Intergenic | -3.954 | MMP8 | ENSG00000118113.7 | ENST00000528662.2 |
| 11 | 3675039 | 3675846 | Intron (ENST00000529556.1/ENSG00000129744.2, intron 1 of 3) | 8.679 | ART1 | ENSG00000129744.2 | ENST00000250693.1 |
| 11 | 62608843 | 62608858 | Promoter (<=1kb) | 0.423 | WDR74 | ENSG00000133316.11 | ENST00000525239.1 |
| 11 | 62608858 | 62608860 | Promoter (<=1kb) | 0.421 | WDR74 | ENSG00000133316.11 | ENST00000525239.1 |
| 11 | 62608860 | 62608888 | Promoter (<=1kb) | 0.393 | WDR74 | ENSG00000133316.11 | ENST00000525239.1 |
| 11 | 62608888 | 62609460 | Promoter (<=1kb) | 0 | WDR74 | ENSG00000133316.11 | ENST00000525239.1 |
| 11 | 1016555 | 1018129 | Promoter (<=1kb) | -0.403 | MUC6 | ENSG00000184956.11 | ENST00000532016.1 |
| 11 | 62609460 | 62609507 | Promoter (<=1kb) | -0.18 | RNU2-2P | ENSG00000222328.1 | ENST00000410396.1 |
| 11 | 62609507 | 62609533 | Promoter (<=1kb) | -0.227 | RNU2-2P | ENSG00000222328.1 | ENST00000410396.1 |
| 11 | 62609533 | 62609541 | Promoter (<=1kb) | -0.253 | RNU2-2P | ENSG00000222328.1 | ENST00000410396.1 |
| 11 | 51583926 | 51585214 | Distal Intergenic | -56.127 | OR4C50P | ENSG00000237610.1 | ENST00000330155.3 |
| 11 | 51585772 | 51587439 | Distal Intergenic | -57.973 | OR4C50P | ENSG00000237610.1 | ENST00000330155.3 |
| 11 | 1.16E+08 | 1.16E+08 | Distal Intergenic | -179.154 | AP000797.2 | ENSG00000239600.1 | ENST00000478870.1 |
| 11 | 1.16E+08 | 1.16E+08 | Distal Intergenic | -179.183 | AP000797.2 | ENSG00000239600.1 | ENST00000478870.1 |
| 11 | 1.16E+08 | 1.16E+08 | Distal Intergenic | -179.573 | AP000797.2 | ENSG00000239600.1 | ENST00000478870.1 |
| 11 | 1.2E+08 | 1.2E+08 | Distal Intergenic | 14.243 | CTD-2523D13.2 | ENSG00000254854.1 | ENST00000533253.1 |
| 11 | 1.2E+08 | 1.2E+08 | Distal Intergenic | 14.245 | CTD-2523D13.2 | ENSG00000254854.1 | ENST00000533253.1 |
| 11 | 1.2E+08 | 1.2E+08 | Distal Intergenic | 14.795 | CTD-2523D13.2 | ENSG00000254854.1 | ENST00000533253.1 |
| 11 | 11267817 | 11267837 | Promoter (2-3kb) | -2.556 | CTD-3224I3.3 | ENSG00000255260.1 | ENST00000524855.1 |
| 11 | 11267837 | 11267838 | Promoter (2-3kb) | -2.576 | CTD-3224I3.3 | ENSG00000255260.1 | ENST00000524855.1 |
| 11 | 11267838 | 11267879 | Promoter (2-3kb) | -2.577 | CTD-3224I3.3 | ENSG00000255260.1 | ENST00000524855.1 |
| 11 | 11267879 | 11268748 | Promoter (2-3kb) | -2.618 | CTD-3224I3.3 | ENSG00000255260.1 | ENST00000524855.1 |
| 11 | 11268748 | 11268761 | Distal Intergenic | -3.487 | CTD-3224I3.3 | ENSG00000255260.1 | ENST00000524855.1 |
| 11 | 11268761 | 11268775 | Distal Intergenic | -3.5 | CTD-3224I3.3 | ENSG00000255260.1 | ENST00000524855.1 |
| 11 | 11268775 | 11268781 | Distal Intergenic | -3.514 | CTD-3224I3.3 | ENSG00000255260.1 | ENST00000524855.1 |
| 12 | 1.11E+08 | 1.11E+08 | Promoter (<=1kb) | 0.254 | ANAPC7 | ENSG00000196510.8 | ENST00000548234.1 |
| 12 | 1.11E+08 | 1.11E+08 | Promoter (<=1kb) | 0.239 | ANAPC7 | ENSG00000196510.8 | ENST00000548234.1 |
| 12 | 1.11E+08 | 1.11E+08 | Promoter (<=1kb) | 0 | ANAPC7 | ENSG00000196510.8 | ENST00000548234.1 |
| 12 | 1.11E+08 | 1.11E+08 | Promoter (<=1kb) | -0.37 | ANAPC7 | ENSG00000196510.8 | ENST00000548234.1 |
| 12 | 1.11E+08 | 1.11E+08 | Promoter (<=1kb) | -0.388 | ANAPC7 | ENSG00000196510.8 | ENST00000548234.1 |
| 12 | 73629 | 73693 | Promoter (<=1kb) | -0.032 | ABC7-42389800N19.1 | ENSG00000226210.3 | ENST00000400706.3 |
| 12 | 73693 | 74919 | Promoter (<=1kb) | 0 | ABC7-42389800N19.1 | ENSG00000226210.3 | ENST00000400706.3 |
| 12 | 74919 | 75006 | Promoter (1-2kb) | 1.195 | ABC7-42389800N19.1 | ENSG00000226210.3 | ENST00000400706.3 |
| 12 | 1.34E+08 | 1.34E+08 | Distal Intergenic | 26.303 | Y_RNA | ENSG00000238443.1 | ENST00000459107.1 |
| 12 | 2829050 | 2829551 | Distal Intergenic | -28.685 | CACNA1C-AS1 | ENSG00000246627.2 | ENST00000544517.1 |
| 12 | 2829551 | 2830273 | Distal Intergenic | -29.186 | CACNA1C-AS1 | ENSG00000246627.2 | ENST00000544517.1 |
| 12 | 94034 | 94750 | Promoter (<=1kb) | -0.605 | DDX11L8 | ENSG00000256263.1 | ENST00000535849.1 |
| 12 | 95108 | 95115 | Promoter (1-2kb) | -1.679 | DDX11L8 | ENSG00000256263.1 | ENST00000535849.1 |
| 12 | 95115 | 95916 | Promoter (1-2kb) | -1.686 | DDX11L8 | ENSG00000256263.1 | ENST00000535849.1 |
| 12 | 34841259 | 34842423 | Distal Intergenic | 438.844 | RP13-7D7.1 | ENSG00000256614.1 | ENST00000540219.1 |
| 12 | 2364889 | 2365339 | Intron (ENST00000335762.5/ENSG00000151067.16, intron 3 of 47) | -13.603 | CACNA1C-IT3 | ENSG00000256721.1 | ENST00000542680.1 |
| 12 | 1.19E+08 | 1.19E+08 | Distal Intergenic | 4.146 | RP11-3L23.2 | ENSG00000256750.1 | ENST00000536572.1 |
| 12 | 1.19E+08 | 1.19E+08 | Distal Intergenic | 3.722 | RP11-3L23.2 | ENSG00000256750.1 | ENST00000536572.1 |
| 12 | 1.19E+08 | 1.19E+08 | Distal Intergenic | 3.707 | RP11-3L23.2 | ENSG00000256750.1 | ENST00000536572.1 |
| 12 | 1.15E+08 | 1.15E+08 | Distal Intergenic | 105.592 | RP4-601P9.1 | ENSG00000257517.1 | ENST00000547876.1 |
| 12 | 1.15E+08 | 1.15E+08 | Distal Intergenic | 105.608 | RP4-601P9.1 | ENSG00000257517.1 | ENST00000547876.1 |
| 12 | 1.15E+08 | 1.15E+08 | Distal Intergenic | 105.625 | RP4-601P9.1 | ENSG00000257517.1 | ENST00000547876.1 |
| 12 | 38096214 | 38096717 | Distal Intergenic | 126.964 | RP11-125N22.2 | ENSG00000258368.1 | ENST00000547456.1 |
| 14 | 1.06E+08 | 1.06E+08 | Distal Intergenic | 5.722 | IGHA2 | ENSG00000211890.3 | ENST00000390539.2 |
| 14 | 1.06E+08 | 1.06E+08 | Distal Intergenic | 5.27 | IGHA2 | ENSG00000211890.3 | ENST00000390539.2 |
| 14 | 1.06E+08 | 1.06E+08 | Distal Intergenic | 5.242 | IGHA2 | ENSG00000211890.3 | ENST00000390539.2 |
| 14 | 19028157 | 19028970 | Distal Intergenic | -81.233 | RP11-754I20.1 | ENSG00000215398.7 | ENST00000514609.2 |
| 14 | 84864254 | 84864877 | Distal Intergenic | 221.492 | CTD-2320B12.2 | ENSG00000259012.2 | ENST00000555160.2 |
| 14 | 84864877 | 84864898 | Distal Intergenic | 222.115 | CTD-2320B12.2 | ENSG00000259012.2 | ENST00000555160.2 |
| 15 | 89868077 | 89868102 | Promoter (<=1kb) | -0.556 | POLG | ENSG00000140521.7 | ENST00000533857.1 |
| 15 | 89868102 | 89868109 | Promoter (<=1kb) | -0.581 | POLG | ENSG00000140521.7 | ENST00000533857.1 |
| 15 | 89868109 | 89868685 | Promoter (<=1kb) | 0.03 | POLG | ENSG00000140521.7 | ENST00000526573.1 |
| 15 | 44580359 | 44580410 | Promoter (<=1kb) | -0.517 | CASC4 | ENSG00000166734.14 | ENST00000559222.1 |
| 15 | 44580410 | 44580416 | Promoter (<=1kb) | -0.511 | CASC4 | ENSG00000166734.14 | ENST00000559222.1 |
| 15 | 44580416 | 44581024 | Promoter (<=1kb) | 0 | CASC4 | ENSG00000166734.14 | ENST00000559222.1 |
| 15 | 44581024 | 44581080 | Promoter (<=1kb) | 0.067 | CASC4 | ENSG00000166734.14 | ENST00000360824.3 |
| 15 | 44581080 | 44581100 | Promoter (<=1kb) | 0.123 | CASC4 | ENSG00000166734.14 | ENST00000360824.3 |
| 15 | 20305716 | 20305722 | Distal Intergenic | -4.435 | RN7SL584P | ENSG00000239471.2 | ENST00000492309.2 |
| 15 | 20305722 | 20307174 | Distal Intergenic | -4.441 | RN7SL584P | ENSG00000239471.2 | ENST00000492309.2 |
| 15 | 20307174 | 20307241 | Distal Intergenic | -5.893 | RN7SL584P | ENSG00000239471.2 | ENST00000492309.2 |
| 15 | 20307458 | 20308006 | Distal Intergenic | -6.177 | RN7SL584P | ENSG00000239471.2 | ENST00000492309.2 |
| 15 | 20310480 | 20310848 | Distal Intergenic | -9.199 | RN7SL584P | ENSG00000239471.2 | ENST00000492309.2 |
| 15 | 21316690 | 21318138 | Distal Intergenic | -8.224 | RN7SL400P | ENSG00000243059.2 | ENST00000473634.2 |
| 15 | 86439341 | 86439374 | Distal Intergenic | 62.828 | RP11-23A22.1 | ENSG00000259608.1 | ENST00000561364.1 |
| 15 | 86439374 | 86439395 | Distal Intergenic | 62.807 | RP11-23A22.1 | ENSG00000259608.1 | ENST00000561364.1 |
| 15 | 86439395 | 86440643 | Distal Intergenic | 61.559 | RP11-23A22.1 | ENSG00000259608.1 | ENST00000561364.1 |
| 15 | 86440643 | 86440649 | Distal Intergenic | 61.553 | RP11-23A22.1 | ENSG00000259608.1 | ENST00000561364.1 |
| 15 | 86440649 | 86440702 | Distal Intergenic | 61.5 | RP11-23A22.1 | ENSG00000259608.1 | ENST00000561364.1 |
| 16 | 33995389 | 33995863 | Distal Intergenic | 29.964 | RNA5-8SP2 | ENSG00000200434.1 | ENST00000363564.1 |
| 16 | 33237270 | 33237275 | Intron (ENST00000398667.4/ENSG00000205457.6, intron 1 of 1) | -5.688 | RP11-1437A8.6 | ENSG00000261507.1 | ENST00000569199.1 |
| 16 | 33237275 | 33238222 | Intron (ENST00000398667.4/ENSG00000205457.6, intron 1 of 1) | -5.693 | RP11-1437A8.6 | ENSG00000261507.1 | ENST00000569199.1 |
| 16 | 33238826 | 33238938 | Intron (ENST00000398667.4/ENSG00000205457.6, intron 1 of 1) | -7.244 | RP11-1437A8.6 | ENSG00000261507.1 | ENST00000569199.1 |
| 16 | 33238938 | 33239621 | Intron (ENST00000398667.4/ENSG00000205457.6, intron 1 of 1) | -6.802 | RP11-1277H1.3 | ENSG00000263337.1 | ENST00000572906.1 |
| 16 | 33239621 | 33239629 | Intron (ENST00000398667.4/ENSG00000205457.6, intron 1 of 1) | -6.794 | RP11-1277H1.3 | ENSG00000263337.1 | ENST00000572906.1 |
| 16 | 33239629 | 33239650 | Intron (ENST00000398667.4/ENSG00000205457.6, intron 1 of 1) | -6.773 | RP11-1277H1.3 | ENSG00000263337.1 | ENST00000572906.1 |
| 16 | 33239668 | 33240090 | Intron (ENST00000398667.4/ENSG00000205457.6, intron 1 of 1) | -6.333 | RP11-1277H1.3 | ENSG00000263337.1 | ENST00000572906.1 |
| 16 | 33240276 | 33241109 | Intron (ENST00000398667.4/ENSG00000205457.6, intron 1 of 1) | -5.314 | RP11-1277H1.3 | ENSG00000263337.1 | ENST00000572906.1 |
| 17 | 72509904 | 72510015 | Distal Intergenic | 17.59 | CD300LB | ENSG00000178789.4 | ENST00000392621.1 |
| 17 | 72510015 | 72511221 | Distal Intergenic | 16.384 | CD300LB | ENSG00000178789.4 | ENST00000392621.1 |
| 17 | 164409 | 165990 | Intron (ENST00000331302.7/ENSG00000181031.11, intron 5 of 9) | 3.281 | RPH3AL | ENSG00000181031.11 | ENST00000576001.1 |
| 17 | 41465843 | 41466782 | Promoter (<=1kb) | 0 | LINC00910 | ENSG00000188825.9 | ENST00000341011.7 |
| 17 | 41466782 | 41466790 | Promoter (<=1kb) | -0.216 | LINC00910 | ENSG00000188825.9 | ENST00000586231.1 |
| 17 | 36348452 | 36349050 | Promoter (<=1kb) | 0 | TBC1D3 | ENSG00000197681.8 | ENST00000520599.1 |
| 17 | 36349050 | 36349343 | Promoter (<=1kb) | -0.385 | TBC1D3 | ENSG00000197681.8 | ENST00000354664.4 |
| 17 | 41381434 | 41382592 | Promoter (<=1kb) | 0 | LINC00854 | ENSG00000236383.3 | ENST00000608223.1 |
| 17 | 22020532 | 22021023 | Promoter (1-2kb) | -1.414 | MTRNR2L1 | ENSG00000256618.1 | ENST00000540040.1 |
| 17 | 22021023 | 22021068 | Promoter (1-2kb) | -1.369 | MTRNR2L1 | ENSG00000256618.1 | ENST00000540040.1 |
| 17 | 22254419 | 22255360 | Distal Intergenic | 50.678 | RP11-846F4.11 | ENSG00000264970.1 | ENST00000578745.1 |
| 17 | 80629857 | 80630279 | Intron (ENST00000571995.1/ENSG00000141542.6, intron 1 of 5) | -3.675 | MIR4525 | ENSG00000266107.1 | ENST00000580000.1 |
| 17 | 21904582 | 21905851 | Promoter (<=1kb) | 0 | RP11-1109M24.11 | ENSG00000266172.1 | ENST00000583831.1 |
| 17 | 22020510 | 22020532 | Promoter (1-2kb) | 1.568 | RP11-846F4.1 | ENSG00000266529.1 | ENST00000578634.1 |
| 17 | 21905851 | 21905864 | Promoter (<=1kb) | -0.553 | RP11-744K17.9 | ENSG00000266795.2 | ENST00000581223.2 |
| 17 | 21905944 | 21905969 | Promoter (<=1kb) | -0.448 | RP11-744K17.9 | ENSG00000266795.2 | ENST00000581223.2 |
| 18 | 63448993 | 63449322 | Intron (ENST00000323011.3/ENSG00000081138.9, intron 2 of 11) | 18.75 | CDH7 | ENSG00000081138.9 | ENST00000581601.1 |
| 18 | 44553719 | 44554117 | Promoter (2-3kb) | 2.332 | TCEB3C | ENSG00000183791.4 | ENST00000330682.2 |
| 18 | 44554952 | 44555082 | Promoter (1-2kb) | 1.367 | TCEB3C | ENSG00000183791.4 | ENST00000330682.2 |
| 18 | 44555115 | 44556182 | Promoter (<=1kb) | 0.267 | TCEB3C | ENSG00000183791.4 | ENST00000330682.2 |
| 18 | 22613294 | 22613349 | Distal Intergenic | -16.127 | WBP2P1 | ENSG00000229248.1 | ENST00000427605.1 |
| 18 | 22613349 | 22613996 | Distal Intergenic | -16.182 | WBP2P1 | ENSG00000229248.1 | ENST00000427605.1 |
| 18 | 22613996 | 22614005 | Distal Intergenic | -16.829 | WBP2P1 | ENSG00000229248.1 | ENST00000427605.1 |
| 18 | 44550238 | 44550886 | Promoter (<=1kb) | 0 | TCEB3CL | ENSG00000234298.1 | ENST00000451265.1 |
| 18 | 44551598 | 44552627 | Promoter (1-2kb) | -1.065 | TCEB3CL | ENSG00000234298.1 | ENST00000451265.1 |
| 18 | 44552699 | 44552713 | Promoter (2-3kb) | -2.166 | TCEB3CL | ENSG00000234298.1 | ENST00000451265.1 |
| 18 | 44552713 | 44553356 | Promoter (2-3kb) | -2.18 | TCEB3CL | ENSG00000234298.1 | ENST00000451265.1 |
| 18 | 44553356 | 44553521 | Promoter (2-3kb) | -2.823 | TCEB3CL | ENSG00000234298.1 | ENST00000451265.1 |
| 18 | 9907 | 10460 | Promoter (<=1kb) | -0.643 | AP005530.1 | ENSG00000262352.1 | ENST00000575820.1 |
| 18 | 10460 | 10469 | Promoter (<=1kb) | -0.634 | AP005530.1 | ENSG00000262352.1 | ENST00000575820.1 |
| 18 | 107280 | 107493 | Promoter (1-2kb) | -1.572 | ROCK1P1 | ENSG00000263006.2 | ENST00000608049.1 |
| 18 | 107699 | 107745 | Promoter (1-2kb) | -1.32 | ROCK1P1 | ENSG00000263006.2 | ENST00000608049.1 |
| 18 | 107745 | 109520 | Promoter (<=1kb) | 0 | ROCK1P1 | ENSG00000263006.2 | ENST00000608049.1 |
| 18 | 109520 | 109521 | Promoter (<=1kb) | 0.456 | ROCK1P1 | ENSG00000263006.2 | ENST00000608049.1 |
| 18 | 109521 | 109557 | Promoter (<=1kb) | 0.457 | ROCK1P1 | ENSG00000263006.2 | ENST00000608049.1 |
| 18 | 109557 | 109564 | Promoter (<=1kb) | 0.493 | ROCK1P1 | ENSG00000263006.2 | ENST00000608049.1 |
| 18 | 109564 | 110918 | Promoter (<=1kb) | 0.5 | ROCK1P1 | ENSG00000263006.2 | ENST00000608049.1 |
| 18 | 110918 | 110922 | Promoter (1-2kb) | -1.445 | ROCK1P1 | ENSG00000263006.2 | ENST00000576266.1 |
| 18 | 110922 | 110925 | Promoter (1-2kb) | -1.442 | ROCK1P1 | ENSG00000263006.2 | ENST00000576266.1 |
| 18 | 110925 | 110945 | Promoter (1-2kb) | -1.422 | ROCK1P1 | ENSG00000263006.2 | ENST00000576266.1 |
| 18 | 111842 | 111883 | Promoter (<=1kb) | -0.484 | ROCK1P1 | ENSG00000263006.2 | ENST00000576266.1 |
| 18 | 111883 | 111915 | Promoter (<=1kb) | -0.452 | ROCK1P1 | ENSG00000263006.2 | ENST00000576266.1 |
| 18 | 111915 | 112643 | Promoter (<=1kb) | 0 | ROCK1P1 | ENSG00000263006.2 | ENST00000576266.1 |
| 18 | 1746908 | 1746910 | Intron (ENST00000580524.1/ENSG00000266450.1, intron 2 of 2) | -33.42 | CTD-2015H3.1 | ENSG00000264080.1 | ENST00000583827.1 |
| 18 | 1746910 | 1746911 | Intron (ENST00000580524.1/ENSG00000266450.1, intron 2 of 2) | -33.419 | CTD-2015H3.1 | ENSG00000264080.1 | ENST00000583827.1 |
| 18 | 1746911 | 1747370 | Intron (ENST00000580524.1/ENSG00000266450.1, intron 2 of 2) | -32.96 | CTD-2015H3.1 | ENSG00000264080.1 | ENST00000583827.1 |
| 18 | 1747370 | 1747373 | Intron (ENST00000580524.1/ENSG00000266450.1, intron 2 of 2) | -32.957 | CTD-2015H3.1 | ENSG00000264080.1 | ENST00000583827.1 |
| 18 | 1747373 | 1747384 | Intron (ENST00000580524.1/ENSG00000266450.1, intron 2 of 2) | -32.946 | CTD-2015H3.1 | ENSG00000264080.1 | ENST00000583827.1 |
| 18 | 44542496 | 44543183 | Promoter (1-2kb) | 1.424 | TCEB3CL2 | ENSG00000266996.2 | ENST00000591973.2 |
| 19 | 7515378 | 7516080 | Exon (ENST00000593531.1/ENSG00000268861.1, exon 9 of 22) | 3.43 | ARHGEF18 | ENSG00000104880.13 | ENST00000594665.1 |
| 19 | 7516080 | 7516117 | Exon (ENST00000593531.1/ENSG00000268861.1, exon 9 of 22) | 4.132 | ARHGEF18 | ENSG00000104880.13 | ENST00000594665.1 |
| 19 | 51002172 | 51002397 | Distal Intergenic | 8.889 | JOSD2 | ENSG00000161677.7 | ENST00000602146.1 |
| 19 | 51002590 | 51002683 | Distal Intergenic | 8.603 | JOSD2 | ENSG00000161677.7 | ENST00000602146.1 |
| 19 | 51002683 | 51003117 | Distal Intergenic | 8.169 | JOSD2 | ENSG00000161677.7 | ENST00000602146.1 |
| 19 | 51003117 | 51003148 | Distal Intergenic | 8.138 | JOSD2 | ENSG00000161677.7 | ENST00000602146.1 |
| 19 | 54922602 | 54922603 | Distal Intergenic | -3.79 | TTYH1 | ENSG00000167614.9 | ENST00000423529.1 |
| 19 | 54922603 | 54922611 | Distal Intergenic | -3.782 | TTYH1 | ENSG00000167614.9 | ENST00000423529.1 |
| 19 | 54922611 | 54923291 | Distal Intergenic | -3.102 | TTYH1 | ENSG00000167614.9 | ENST00000423529.1 |
| 19 | 37784058 | 37784081 | Distal Intergenic | -19.658 | HKR1 | ENSG00000181666.13 | ENST00000591471.1 |
| 19 | 37784081 | 37784136 | Distal Intergenic | -19.603 | HKR1 | ENSG00000181666.13 | ENST00000591471.1 |
| 19 | 37784136 | 37784195 | Distal Intergenic | -19.544 | HKR1 | ENSG00000181666.13 | ENST00000591471.1 |
| 19 | 37784195 | 37785807 | Distal Intergenic | -17.932 | HKR1 | ENSG00000181666.13 | ENST00000591471.1 |
| 19 | 37785807 | 37785908 | Distal Intergenic | -17.831 | HKR1 | ENSG00000181666.13 | ENST00000591471.1 |
| 19 | 37785908 | 37785938 | Distal Intergenic | -17.801 | HKR1 | ENSG00000181666.13 | ENST00000591471.1 |
| 19 | 37785938 | 37786027 | Distal Intergenic | -17.712 | HKR1 | ENSG00000181666.13 | ENST00000591471.1 |
| 19 | 37786027 | 37786065 | Distal Intergenic | -17.674 | HKR1 | ENSG00000181666.13 | ENST00000591471.1 |
| 19 | 37788314 | 37789196 | Distal Intergenic | -14.543 | HKR1 | ENSG00000181666.13 | ENST00000591471.1 |
| 19 | 37789196 | 37789723 | Distal Intergenic | -14.016 | HKR1 | ENSG00000181666.13 | ENST00000591471.1 |
| 19 | 37790168 | 37790227 | Distal Intergenic | -13.512 | HKR1 | ENSG00000181666.13 | ENST00000591471.1 |
| 19 | 37790227 | 37791182 | Distal Intergenic | -12.557 | HKR1 | ENSG00000181666.13 | ENST00000591471.1 |
| 19 | 37791182 | 37791927 | Distal Intergenic | -11.812 | HKR1 | ENSG00000181666.13 | ENST00000591471.1 |
| 19 | 37791927 | 37792026 | Distal Intergenic | -11.713 | HKR1 | ENSG00000181666.13 | ENST00000591471.1 |
| 19 | 37792026 | 37792034 | Distal Intergenic | -11.705 | HKR1 | ENSG00000181666.13 | ENST00000591471.1 |
| 19 | 37792042 | 37792141 | Distal Intergenic | -11.598 | HKR1 | ENSG00000181666.13 | ENST00000591471.1 |
| 19 | 37793795 | 37794466 | Distal Intergenic | -9.273 | HKR1 | ENSG00000181666.13 | ENST00000591471.1 |
| 19 | 36765114 | 36765185 | Distal Intergenic | -27.956 | ZNF565 | ENSG00000196357.7 | ENST00000355114.5 |
| 19 | 36765185 | 36765969 | Distal Intergenic | -28.027 | ZNF565 | ENSG00000196357.7 | ENST00000355114.5 |
| 19 | 36765969 | 36766119 | Distal Intergenic | -28.811 | ZNF565 | ENSG00000196357.7 | ENST00000355114.5 |
| 19 | 36766564 | 36768016 | Distal Intergenic | -27.466 | CTD-3162L10.1 | ENSG00000267053.2 | ENST00000586345.1 |
| 19 | 36771443 | 36771466 | Distal Intergenic | -24.016 | CTD-3162L10.1 | ENSG00000267053.2 | ENST00000586345.1 |
| 19 | 36771466 | 36773182 | Distal Intergenic | -22.3 | CTD-3162L10.1 | ENSG00000267053.2 | ENST00000586345.1 |
| 19 | 36778602 | 36778668 | Distal Intergenic | -16.814 | CTD-3162L10.1 | ENSG00000267053.2 | ENST00000586345.1 |
| 19 | 36778668 | 36779290 | Distal Intergenic | -16.192 | CTD-3162L10.1 | ENSG00000267053.2 | ENST00000586345.1 |
| 19 | 36779290 | 36780092 | Distal Intergenic | -15.39 | CTD-3162L10.1 | ENSG00000267053.2 | ENST00000586345.1 |
| 19 | 36780398 | 36780421 | Distal Intergenic | -15.061 | CTD-3162L10.1 | ENSG00000267053.2 | ENST00000586345.1 |
| 19 | 36780421 | 36780499 | Distal Intergenic | -14.983 | CTD-3162L10.1 | ENSG00000267053.2 | ENST00000586345.1 |
| 19 | 36780499 | 36780592 | Distal Intergenic | -14.89 | CTD-3162L10.1 | ENSG00000267053.2 | ENST00000586345.1 |
| 19 | 36780996 | 36781382 | Distal Intergenic | -14.1 | CTD-3162L10.1 | ENSG00000267053.2 | ENST00000586345.1 |
| 19 | 36784337 | 36784395 | Distal Intergenic | -11.087 | CTD-3162L10.1 | ENSG00000267053.2 | ENST00000586345.1 |
| 19 | 36784395 | 36785753 | Distal Intergenic | -9.729 | CTD-3162L10.1 | ENSG00000267053.2 | ENST00000586345.1 |
| 19 | 36787836 | 36787927 | Distal Intergenic | -7.555 | CTD-3162L10.1 | ENSG00000267053.2 | ENST00000586345.1 |
| 19 | 36787927 | 36788207 | Distal Intergenic | -7.275 | CTD-3162L10.1 | ENSG00000267053.2 | ENST00000586345.1 |
| 19 | 36790558 | 36790888 | Distal Intergenic | -4.594 | CTD-3162L10.1 | ENSG00000267053.2 | ENST00000586345.1 |
| 19 | 36790888 | 36790937 | Distal Intergenic | -4.545 | CTD-3162L10.1 | ENSG00000267053.2 | ENST00000586345.1 |
| 19 | 36791207 | 36791226 | Distal Intergenic | -4.256 | CTD-3162L10.1 | ENSG00000267053.2 | ENST00000586345.1 |
| 19 | 36791226 | 36792022 | Distal Intergenic | -3.46 | CTD-3162L10.1 | ENSG00000267053.2 | ENST00000586345.1 |
| 19 | 36792022 | 36792203 | Distal Intergenic | -3.279 | CTD-3162L10.1 | ENSG00000267053.2 | ENST00000586345.1 |
| 19 | 36792320 | 36793161 | Promoter (2-3kb) | -2.321 | CTD-3162L10.1 | ENSG00000267053.2 | ENST00000586345.1 |
| 19 | 36800144 | 36800491 | Promoter (<=1kb) | -0.769 | CTD-3162L10.1 | ENSG00000267053.2 | ENST00000600983.1 |
| 19 | 36800491 | 36800731 | Promoter (<=1kb) | -0.529 | CTD-3162L10.1 | ENSG00000267053.2 | ENST00000600983.1 |
| 19 | 44914925 | 44915098 | Intron (ENST00000588655.1/ENSG00000267188.1, intron 4 of 5) | -9.152 | ZNF285 | ENSG00000267508.1 | ENST00000585868.1 |
| 19 | 44915098 | 44915860 | Intron (ENST00000588655.1/ENSG00000267188.1, intron 4 of 5) | -9.325 | ZNF285 | ENSG00000267508.1 | ENST00000585868.1 |
| 19 | 37760605 | 37762387 | Promoter (<=1kb) | 0 | CTD-3220F14.1 | ENSG00000267605.1 | ENST00000588763.1 |
| 19 | 37763174 | 37764070 | Promoter (<=1kb) | -0.755 | CTD-3220F14.1 | ENSG00000267605.1 | ENST00000586442.1 |
| 19 | 37771180 | 37771553 | Distal Intergenic | -8.761 | CTD-3220F14.1 | ENSG00000267605.1 | ENST00000586442.1 |
| 19 | 37771553 | 37771681 | Distal Intergenic | -9.134 | CTD-3220F14.1 | ENSG00000267605.1 | ENST00000586442.1 |
| 19 | 37771681 | 37771751 | Distal Intergenic | -9.262 | CTD-3220F14.1 | ENSG00000267605.1 | ENST00000586442.1 |
| 19 | 37771751 | 37771940 | Distal Intergenic | -9.332 | CTD-3220F14.1 | ENSG00000267605.1 | ENST00000586442.1 |
| 19 | 37771940 | 37772591 | Distal Intergenic | -9.521 | CTD-3220F14.1 | ENSG00000267605.1 | ENST00000586442.1 |
| 19 | 37772591 | 37772687 | Distal Intergenic | -10.172 | CTD-3220F14.1 | ENSG00000267605.1 | ENST00000586442.1 |
| 19 | 37772687 | 37772726 | Distal Intergenic | -10.268 | CTD-3220F14.1 | ENSG00000267605.1 | ENST00000586442.1 |
| 19 | 37772726 | 37772754 | Distal Intergenic | -10.307 | CTD-3220F14.1 | ENSG00000267605.1 | ENST00000586442.1 |
| 19 | 37772807 | 37772813 | Distal Intergenic | -10.388 | CTD-3220F14.1 | ENSG00000267605.1 | ENST00000586442.1 |
| 19 | 37772813 | 37773468 | Distal Intergenic | -10.394 | CTD-3220F14.1 | ENSG00000267605.1 | ENST00000586442.1 |
| 19 | 37773468 | 37773492 | Distal Intergenic | -11.049 | CTD-3220F14.1 | ENSG00000267605.1 | ENST00000586442.1 |
| 19 | 37775409 | 37776894 | Distal Intergenic | -12.99 | CTD-3220F14.1 | ENSG00000267605.1 | ENST00000586442.1 |
| 19 | 37779652 | 37779675 | Distal Intergenic | -17.233 | CTD-3220F14.1 | ENSG00000267605.1 | ENST00000586442.1 |
| 19 | 37779675 | 37779762 | Distal Intergenic | -17.256 | CTD-3220F14.1 | ENSG00000267605.1 | ENST00000586442.1 |
| 19 | 37779762 | 37780377 | Distal Intergenic | -17.343 | CTD-3220F14.1 | ENSG00000267605.1 | ENST00000586442.1 |
| 19 | 37780377 | 37781150 | Distal Intergenic | -17.958 | CTD-3220F14.1 | ENSG00000267605.1 | ENST00000586442.1 |
| 19 | 37781150 | 37781301 | Distal Intergenic | -18.731 | CTD-3220F14.1 | ENSG00000267605.1 | ENST00000586442.1 |
| 20 | 29829137 | 29829943 | Distal Intergenic | -15.524 | DEFB115 | ENSG00000215547.1 | ENST00000400552.1 |
| 20 | 22742745 | 22742751 | Distal Intergenic | -28.019 | KRT18P3 | ENSG00000215553.3 | ENST00000451602.1 |
| 20 | 22742751 | 22742764 | Distal Intergenic | -28.025 | KRT18P3 | ENSG00000215553.3 | ENST00000451602.1 |
| 20 | 22742764 | 22743217 | Distal Intergenic | -28.038 | KRT18P3 | ENSG00000215553.3 | ENST00000451602.1 |
| 20 | 22743217 | 22743228 | Distal Intergenic | -28.491 | KRT18P3 | ENSG00000215553.3 | ENST00000451602.1 |
| 20 | 26313312 | 26313582 | Distal Intergenic | -81.151 | MIR663A | ENSG00000227195.4 | ENST00000601119.1 |
| 20 | 62324663 | 62325153 | Promoter (2-3kb) | -2.868 | TNFRSF6B | ENSG00000243509.4 | ENST00000369996.1 |
| 21 | 47400327 | 47400335 | Promoter (1-2kb) | -1.316 | COL6A1 | ENSG00000142156.10 | ENST00000361866.3 |
| 21 | 47400335 | 47400350 | Promoter (1-2kb) | -1.301 | COL6A1 | ENSG00000142156.10 | ENST00000361866.3 |
| 21 | 47400350 | 47401139 | Promoter (<=1kb) | -0.512 | COL6A1 | ENSG00000142156.10 | ENST00000361866.3 |
| 21 | 47401139 | 47401170 | Promoter (<=1kb) | -0.481 | COL6A1 | ENSG00000142156.10 | ENST00000361866.3 |
| 21 | 47401170 | 47401204 | Promoter (<=1kb) | -0.447 | COL6A1 | ENSG00000142156.10 | ENST00000361866.3 |
| 21 | 34143858 | 34143959 | Promoter (<=1kb) | 0.02 | PAXBP1 | ENSG00000159086.10 | ENST00000290178.4 |
| 21 | 34143959 | 34143960 | Promoter (<=1kb) | 0.019 | PAXBP1 | ENSG00000159086.10 | ENST00000290178.4 |
| 21 | 34143960 | 34144547 | Promoter (<=1kb) | 0 | PAXBP1 | ENSG00000159086.10 | ENST00000331923.4 |
| 21 | 10698256 | 10698432 | Distal Intergenic | -164.19 | IGHV1OR21-1 | ENSG00000169861.8 | ENST00000302092.5 |
| 21 | 10698432 | 10698444 | Distal Intergenic | -164.178 | IGHV1OR21-1 | ENSG00000169861.8 | ENST00000302092.5 |
| 21 | 10698444 | 10698457 | Distal Intergenic | -164.165 | IGHV1OR21-1 | ENSG00000169861.8 | ENST00000302092.5 |
| 21 | 10698457 | 10698487 | Distal Intergenic | -164.135 | IGHV1OR21-1 | ENSG00000169861.8 | ENST00000302092.5 |
| 21 | 10698487 | 10698495 | Distal Intergenic | -164.127 | IGHV1OR21-1 | ENSG00000169861.8 | ENST00000302092.5 |
| 21 | 10698495 | 10698560 | Distal Intergenic | -164.062 | IGHV1OR21-1 | ENSG00000169861.8 | ENST00000302092.5 |
| 21 | 10698560 | 10698703 | Distal Intergenic | -163.919 | IGHV1OR21-1 | ENSG00000169861.8 | ENST00000302092.5 |
| 21 | 10698703 | 10698901 | Distal Intergenic | -163.721 | IGHV1OR21-1 | ENSG00000169861.8 | ENST00000302092.5 |
| 21 | 10698901 | 10699032 | Distal Intergenic | -163.59 | IGHV1OR21-1 | ENSG00000169861.8 | ENST00000302092.5 |
| 21 | 10699032 | 10699091 | Distal Intergenic | -163.531 | IGHV1OR21-1 | ENSG00000169861.8 | ENST00000302092.5 |
| 21 | 10699091 | 10699120 | Distal Intergenic | -163.502 | IGHV1OR21-1 | ENSG00000169861.8 | ENST00000302092.5 |
| 21 | 10699120 | 10699238 | Distal Intergenic | -163.384 | IGHV1OR21-1 | ENSG00000169861.8 | ENST00000302092.5 |
| 21 | 10699238 | 10699606 | Distal Intergenic | -163.016 | IGHV1OR21-1 | ENSG00000169861.8 | ENST00000302092.5 |
| 21 | 10699606 | 10699719 | Distal Intergenic | -162.903 | IGHV1OR21-1 | ENSG00000169861.8 | ENST00000302092.5 |
| 21 | 10699719 | 10699726 | Distal Intergenic | -162.896 | IGHV1OR21-1 | ENSG00000169861.8 | ENST00000302092.5 |
| 21 | 10700997 | 10701124 | Distal Intergenic | -161.498 | IGHV1OR21-1 | ENSG00000169861.8 | ENST00000302092.5 |
| 21 | 10701124 | 10701365 | Distal Intergenic | -161.257 | IGHV1OR21-1 | ENSG00000169861.8 | ENST00000302092.5 |
| 21 | 10701365 | 10701794 | Distal Intergenic | -160.828 | IGHV1OR21-1 | ENSG00000169861.8 | ENST00000302092.5 |
| 21 | 10774227 | 10774734 | Distal Intergenic | -87.888 | IGHV1OR21-1 | ENSG00000169861.8 | ENST00000302092.5 |
| 21 | 10806769 | 10807964 | Distal Intergenic | -54.658 | IGHV1OR21-1 | ENSG00000169861.8 | ENST00000302092.5 |
| 21 | 41346483 | 41346966 | Distal Intergenic | 76.083 | PCP4 | ENSG00000183036.6 | ENST00000467565.1 |
| 21 | 34144547 | 34144556 | Promoter (<=1kb) | 0.049 | C21orf49 | ENSG00000205930.4 | ENST00000477513.1 |
| 21 | 34144556 | 34144594 | Promoter (<=1kb) | 0.058 | C21orf49 | ENSG00000205930.4 | ENST00000477513.1 |
| 21 | 48119581 | 48119754 | Distal Intergenic | 8.787 | RPL23AP4 | ENSG00000212932.2 | ENST00000434638.1 |
| 21 | 40114763 | 40115467 | Promoter (<=1kb) | -0.323 | LINC00114 | ENSG00000223806.3 | ENST00000411989.1 |
| 21 | 40115467 | 40115469 | Promoter (1-2kb) | -1.027 | LINC00114 | ENSG00000223806.3 | ENST00000411989.1 |
| 21 | 40115469 | 40115519 | Promoter (1-2kb) | -1.029 | LINC00114 | ENSG00000223806.3 | ENST00000411989.1 |
| 21 | 11185884 | 11187066 | Distal Intergenic | -4.07 | EIF3FP1 | ENSG00000234643.1 | ENST00000414503.1 |
| 21 | 9695485 | 9695491 | Distal Intergenic | 12.295 | CR381670.1 | ENSG00000238411.1 | ENST00000459169.1 |
| 21 | 9695491 | 9696392 | Distal Intergenic | 12.301 | CR381670.1 | ENSG00000238411.1 | ENST00000459169.1 |
| 21 | 9698503 | 9698518 | Distal Intergenic | 15.313 | CR381670.1 | ENSG00000238411.1 | ENST00000459169.1 |
| 21 | 9698518 | 9698763 | Distal Intergenic | 15.328 | CR381670.1 | ENSG00000238411.1 | ENST00000459169.1 |
| 21 | 9698763 | 9698784 | Distal Intergenic | 15.573 | CR381670.1 | ENSG00000238411.1 | ENST00000459169.1 |
| 21 | 9698784 | 9700270 | Distal Intergenic | 15.594 | CR381670.1 | ENSG00000238411.1 | ENST00000459169.1 |
| 21 | 9700270 | 9700300 | Distal Intergenic | 17.08 | CR381670.1 | ENSG00000238411.1 | ENST00000459169.1 |
| 21 | 9700300 | 9700314 | Distal Intergenic | 17.11 | CR381670.1 | ENSG00000238411.1 | ENST00000459169.1 |
| 21 | 9700314 | 9700343 | Distal Intergenic | 17.124 | CR381670.1 | ENSG00000238411.1 | ENST00000459169.1 |
| 21 | 9825537 | 9826535 | Promoter (<=1kb) | 0 | MIR3687 | ENSG00000264063.1 | ENST00000577708.1 |
| 21 | 9826535 | 9826543 | Promoter (<=1kb) | 0.333 | MIR3687 | ENSG00000264063.1 | ENST00000577708.1 |
| 21 | 9826543 | 9826567 | Promoter (<=1kb) | 0.341 | MIR3687 | ENSG00000264063.1 | ENST00000577708.1 |
| 21 | 9826609 | 9826611 | Promoter (<=1kb) | 0.407 | MIR3687 | ENSG00000264063.1 | ENST00000577708.1 |
| 21 | 9826611 | 9826621 | Promoter (<=1kb) | 0.409 | MIR3687 | ENSG00000264063.1 | ENST00000577708.1 |
| 21 | 9826621 | 9826626 | Promoter (<=1kb) | 0.419 | MIR3687 | ENSG00000264063.1 | ENST00000577708.1 |
| 21 | 9826626 | 9827579 | Promoter (<=1kb) | 0.424 | MIR3687 | ENSG00000264063.1 | ENST00000577708.1 |
| 21 | 9827579 | 9827635 | Promoter (1-2kb) | 1.377 | MIR3687 | ENSG00000264063.1 | ENST00000577708.1 |
| 21 | 9827635 | 9827641 | Promoter (1-2kb) | 1.433 | MIR3687 | ENSG00000264063.1 | ENST00000577708.1 |
| 21 | 9825506 | 9825510 | Promoter (<=1kb) | -0.322 | MIR3648 | ENSG00000264462.1 | ENST00000581792.1 |
| 21 | 9825510 | 9825523 | Promoter (<=1kb) | -0.309 | MIR3648 | ENSG00000264462.1 | ENST00000581792.1 |
| 21 | 9825523 | 9825537 | Promoter (<=1kb) | -0.295 | MIR3648 | ENSG00000264462.1 | ENST00000581792.1 |
| 22 | 37724688 | 37725348 | Distal Intergenic | 18.68 | CYTH4 | ENSG00000100055.16 | ENST00000446506.1 |
| 22 | 51082024 | 51082722 | Distal Intergenic | -15.418 | ARSA | ENSG00000100299.13 | ENST00000547805.1 |
| 22 | 18717662 | 18717665 | Distal Intergenic | -3.762 | AC008132.1 | ENSG00000182824.6 | ENST00000342888.3 |
| 22 | 18717665 | 18717674 | Distal Intergenic | -3.753 | AC008132.1 | ENSG00000182824.6 | ENST00000342888.3 |
| 22 | 18717674 | 18717734 | Distal Intergenic | -3.693 | AC008132.1 | ENSG00000182824.6 | ENST00000342888.3 |
| 22 | 18717734 | 18718386 | Distal Intergenic | -3.041 | AC008132.1 | ENSG00000182824.6 | ENST00000342888.3 |
| 22 | 18718386 | 18718388 | Distal Intergenic | -3.039 | AC008132.1 | ENSG00000182824.6 | ENST00000342888.3 |
| 22 | 18721110 | 18721773 | Promoter (<=1kb) | 0 | AC008132.1 | ENSG00000182824.6 | ENST00000342888.3 |
| 22 | 18721773 | 18721851 | Promoter (<=1kb) | 0.347 | AC008132.1 | ENSG00000182824.6 | ENST00000342888.3 |
| 22 | 18877892 | 18877900 | Distal Intergenic | -15.641 | DGCR6 | ENSG00000183628.8 | ENST00000413981.1 |
| 22 | 18877900 | 18878732 | Distal Intergenic | -14.809 | DGCR6 | ENSG00000183628.8 | ENST00000413981.1 |
| 22 | 18878732 | 18878745 | Distal Intergenic | -14.796 | DGCR6 | ENSG00000183628.8 | ENST00000413981.1 |
| 22 | 18879643 | 18880339 | Distal Intergenic | -13.202 | DGCR6 | ENSG00000183628.8 | ENST00000413981.1 |
| 22 | 18880339 | 18880354 | Distal Intergenic | -13.187 | DGCR6 | ENSG00000183628.8 | ENST00000413981.1 |
| 22 | 18883482 | 18884329 | Distal Intergenic | -9.212 | DGCR6 | ENSG00000183628.8 | ENST00000413981.1 |
| 22 | 18884329 | 18884374 | Distal Intergenic | -9.167 | DGCR6 | ENSG00000183628.8 | ENST00000413981.1 |
| 22 | 25489443 | 25489552 | Intron (ENST00000406486.4/ENSG00000197077.8, intron 7 of 13) | 19.107 | CTA-221G9.11 | ENSG00000203280.3 | ENST00000366110.3 |
| 22 | 25489552 | 25489956 | Intron (ENST00000406486.4/ENSG00000197077.8, intron 7 of 13) | 18.703 | CTA-221G9.11 | ENSG00000203280.3 | ENST00000366110.3 |
| 22 | 25489956 | 25489979 | Intron (ENST00000406486.4/ENSG00000197077.8, intron 7 of 13) | 18.68 | CTA-221G9.11 | ENSG00000203280.3 | ENST00000366110.3 |
| 22 | 21521781 | 21521997 | Promoter (<=1kb) | -0.05 | FAM230B | ENSG00000215498.4 | ENST00000451257.1 |
| 22 | 21521997 | 21522644 | Promoter (<=1kb) | 0 | FAM230B | ENSG00000215498.4 | ENST00000451257.1 |
| 22 | 34792370 | 34792400 | Distal Intergenic | -187.12 | LL22NC03-13G6.2 | ENSG00000224404.1 | ENST00000450365.1 |
| 22 | 34792400 | 34792805 | Distal Intergenic | -187.15 | LL22NC03-13G6.2 | ENSG00000224404.1 | ENST00000450365.1 |
| 22 | 34792805 | 34792832 | Distal Intergenic | -187.555 | LL22NC03-13G6.2 | ENSG00000224404.1 | ENST00000450365.1 |
| X | 1800279 | 1800284 | Distal Intergenic | 44.989 | ASMT | ENSG00000196433.7 | ENST00000432523.1 |
| X | 1800284 | 1800325 | Distal Intergenic | 44.994 | ASMT | ENSG00000196433.7 | ENST00000432523.1 |
| X | 1800325 | 1800380 | Distal Intergenic | 45.035 | ASMT | ENSG00000196433.7 | ENST00000432523.1 |
| X | 1800380 | 1800951 | Distal Intergenic | 45.09 | ASMT | ENSG00000196433.7 | ENST00000432523.1 |
| X | 1800951 | 1800953 | Distal Intergenic | 45.661 | ASMT | ENSG00000196433.7 | ENST00000432523.1 |
| X | 1800953 | 1801085 | Distal Intergenic | 45.663 | ASMT | ENSG00000196433.7 | ENST00000432523.1 |
| X | 1801085 | 1801433 | Distal Intergenic | 45.795 | ASMT | ENSG00000196433.7 | ENST00000432523.1 |
| X | 1259408 | 1259520 | Distal Intergenic | 72.007 | CRLF2 | ENSG00000205755.6 | ENST00000381567.3 |
| X | 1259520 | 1261273 | Distal Intergenic | 70.254 | CRLF2 | ENSG00000205755.6 | ENST00000381567.3 |
| X | 1261273 | 1261295 | Distal Intergenic | 70.232 | CRLF2 | ENSG00000205755.6 | ENST00000381567.3 |
| X | 283227 | 283926 | Promoter (1-2kb) | 1.503 | LINC00685 | ENSG00000226179.1 | ENST00000391707.2 |
| X | 283926 | 284883 | Promoter (2-3kb) | 2.202 | LINC00685 | ENSG00000226179.1 | ENST00000391707.2 |
| X | 284883 | 284933 | Distal Intergenic | 3.159 | LINC00685 | ENSG00000226179.1 | ENST00000391707.2 |
| X | 1.55E+08 | 1.55E+08 | Promoter (1-2kb) | -1.647 | DDX11L16 | ENSG00000227159.3 | ENST00000445777.1 |
| X | 1.55E+08 | 1.55E+08 | Promoter (1-2kb) | -1.791 | DDX11L16 | ENSG00000227159.3 | ENST00000445777.1 |
| X | 1.55E+08 | 1.55E+08 | Promoter (1-2kb) | -1.847 | DDX11L16 | ENSG00000227159.3 | ENST00000445777.1 |
| X | 1.55E+08 | 1.55E+08 | Promoter (1-2kb) | -1.879 | DDX11L16 | ENSG00000227159.3 | ENST00000445777.1 |
| X | 1.55E+08 | 1.55E+08 | Promoter (1-2kb) | -1.893 | DDX11L16 | ENSG00000227159.3 | ENST00000445777.1 |
| X | 1.15E+08 | 1.15E+08 | Distal Intergenic | 44.214 | RP1-241P17.1 | ENSG00000229335.1 | ENST00000415394.1 |
| X | 1.15E+08 | 1.15E+08 | Distal Intergenic | 44.254 | RP1-241P17.1 | ENSG00000229335.1 | ENST00000415394.1 |
| X | 1.15E+08 | 1.15E+08 | Distal Intergenic | 45.611 | RP1-241P17.1 | ENSG00000229335.1 | ENST00000415394.1 |
| X | 1.15E+08 | 1.15E+08 | Distal Intergenic | 46.213 | RP1-241P17.1 | ENSG00000229335.1 | ENST00000415394.1 |
| X | 61693273 | 61693683 | Distal Intergenic | -305.037 | RP11-3D23.1 | ENSG00000236852.1 | ENST00000455793.1 |
| X | 61699764 | 61699787 | Distal Intergenic | -298.933 | RP11-3D23.1 | ENSG00000236852.1 | ENST00000455793.1 |
| X | 61699787 | 61700183 | Distal Intergenic | -298.537 | RP11-3D23.1 | ENSG00000236852.1 | ENST00000455793.1 |
| X | 61700240 | 61701486 | Distal Intergenic | -297.234 | RP11-3D23.1 | ENSG00000236852.1 | ENST00000455793.1 |
| X | 61703725 | 61704594 | Distal Intergenic | -294.126 | RP11-3D23.1 | ENSG00000236852.1 | ENST00000455793.1 |
| X | 61704594 | 61704624 | Distal Intergenic | -294.096 | RP11-3D23.1 | ENSG00000236852.1 | ENST00000455793.1 |
| Y | 13842341 | 13843121 | Distal Intergenic | 60.112 | RCC2P1 | ENSG00000234385.1 | ENST00000452257.1 |
| Y | 13868207 | 13868806 | Distal Intergenic | 34.427 | RCC2P1 | ENSG00000234385.1 | ENST00000452257.1 |
| Y | 58975968 | 58976193 | Distal Intergenic | -25.198 | CTBP2P1 | ENSG00000235857.1 | ENST00000431853.1 |
| Y | 58976665 | 58976935 | Distal Intergenic | -24.456 | CTBP2P1 | ENSG00000235857.1 | ENST00000431853.1 |
| Y | 58976935 | 58977073 | Distal Intergenic | -24.318 | CTBP2P1 | ENSG00000235857.1 | ENST00000431853.1 |
| Y | 58977073 | 58977451 | Distal Intergenic | -23.94 | CTBP2P1 | ENSG00000235857.1 | ENST00000431853.1 |
| Y | 58977451 | 58977529 | Distal Intergenic | -23.862 | CTBP2P1 | ENSG00000235857.1 | ENST00000431853.1 |
| Y | 58977760 | 58978309 | Distal Intergenic | -23.082 | CTBP2P1 | ENSG00000235857.1 | ENST00000431853.1 |
| Y | 58978309 | 58979061 | Distal Intergenic | -22.33 | CTBP2P1 | ENSG00000235857.1 | ENST00000431853.1 |
| Y | 58979346 | 58979785 | Distal Intergenic | -21.606 | CTBP2P1 | ENSG00000235857.1 | ENST00000431853.1 |
| Y | 58979832 | 58979842 | Distal Intergenic | -21.549 | CTBP2P1 | ENSG00000235857.1 | ENST00000431853.1 |
| Y | 58979842 | 58980928 | Distal Intergenic | -20.463 | CTBP2P1 | ENSG00000235857.1 | ENST00000431853.1 |
| Y | 28815230 | 28815284 | Distal Intergenic | -34.432 | PARP4P1 | ENSG00000237917.1 | ENST00000435945.1 |
| Y | 28815284 | 28815332 | Distal Intergenic | -34.486 | PARP4P1 | ENSG00000237917.1 | ENST00000435945.1 |
| Y | 28815332 | 28815780 | Distal Intergenic | -34.534 | PARP4P1 | ENSG00000237917.1 | ENST00000435945.1 |
| Y | 28816508 | 28818056 | Distal Intergenic | -35.71 | PARP4P1 | ENSG00000237917.1 | ENST00000435945.1 |
| Y | 28818502 | 28819075 | Distal Intergenic | -37.704 | PARP4P1 | ENSG00000237917.1 | ENST00000435945.1 |
| Y | 13446335 | 13446981 | Distal Intergenic | -15.613 | DUX4L16 | ENSG00000258567.1 | ENST00000555130.1 |
| Y | 13446981 | 13447006 | Distal Intergenic | -15.588 | DUX4L16 | ENSG00000258567.1 | ENST00000555130.1 |
| Y | 13447155 | 13447622 | Distal Intergenic | -14.972 | DUX4L16 | ENSG00000258567.1 | ENST00000555130.1 |
| Y | 13447714 | 13447817 | Distal Intergenic | -14.777 | DUX4L16 | ENSG00000258567.1 | ENST00000555130.1 |
| Y | 13447817 | 13447834 | Distal Intergenic | -14.76 | DUX4L16 | ENSG00000258567.1 | ENST00000555130.1 |
| Y | 13448086 | 13448643 | Distal Intergenic | -13.951 | DUX4L16 | ENSG00000258567.1 | ENST00000555130.1 |
| Y | 13448643 | 13448707 | Distal Intergenic | -13.887 | DUX4L16 | ENSG00000258567.1 | ENST00000555130.1 |
| Y | 13450786 | 13451707 | Distal Intergenic | -10.887 | DUX4L16 | ENSG00000258567.1 | ENST00000555130.1 |
| Y | 13484686 | 13486108 | Promoter (1-2kb) | -1.897 | DUX4L19 | ENSG00000258991.1 | ENST00000557448.1 |
| Y | 13488196 | 13488197 | Promoter (<=1kb) | 0.192 | DUX4L19 | ENSG00000258991.1 | ENST00000557448.1 |
| Y | 13488197 | 13488207 | Promoter (<=1kb) | 0.193 | DUX4L19 | ENSG00000258991.1 | ENST00000557448.1 |
| Y | 13488207 | 13488309 | Promoter (<=1kb) | 0.203 | DUX4L19 | ENSG00000258991.1 | ENST00000557448.1 |
| Y | 13488309 | 13490153 | Promoter (<=1kb) | 0.305 | DUX4L19 | ENSG00000258991.1 | ENST00000557448.1 |
| Y | 13478114 | 13478767 | Promoter (<=1kb) | 0.882 | DUX4L18 | ENSG00000259029.1 | ENST00000553347.1 |
| Y | 13467664 | 13467775 | Promoter (2-3kb) | -2.822 | DUX4L17 | ENSG00000259154.1 | ENST00000557360.1 |
| Y | 13467775 | 13467798 | Promoter (2-3kb) | -2.799 | DUX4L17 | ENSG00000259154.1 | ENST00000557360.1 |
| Y | 13467798 | 13469609 | Promoter (<=1kb) | -0.988 | DUX4L17 | ENSG00000259154.1 | ENST00000557360.1 |
| Y | 13469609 | 13469646 | Promoter (<=1kb) | -0.951 | DUX4L17 | ENSG00000259154.1 | ENST00000557360.1 |
| Y | 13469646 | 13469684 | Promoter (<=1kb) | -0.913 | DUX4L17 | ENSG00000259154.1 | ENST00000557360.1 |
| Y | 13490153 | 13490165 | Promoter (1-2kb) | -1.138 | PABPC1P5 | ENSG00000270455.1 | ENST00000603738.1 |
| Y | 13140784 | 13141470 | Distal Intergenic | 121.469 | RP1-85D24.3 | ENSG00000271365.1 | ENST00000605663.1 |
| Y | 13141470 | 13141471 | Distal Intergenic | 121.468 | RP1-85D24.3 | ENSG00000271365.1 | ENST00000605663.1 |
| Y | 13704269 | 13704750 | Distal Intergenic | -74.357 | RP11-295P22.2 | ENSG00000271375.1 | ENST00000604178.1 |
| Y | 13712378 | 13713636 | Distal Intergenic | -82.466 | RP11-295P22.2 | ENSG00000271375.1 | ENST00000604178.1 |
| Y | 13713636 | 13713675 | Distal Intergenic | -83.724 | RP11-295P22.2 | ENSG00000271375.1 | ENST00000604178.1 |

Supplementary Table S4: Sites of IGF-1R recruitment identified in cultured prostate cancer cells and clinical prostate cancers. Table shows chromosomal (Chr) location of IGF-1R peaks identified in both re-analysis of our previous DU145 cell line ChIP-seq (Aleksic et al., 2018) and identified in ChIP-seq performed here from fresh RP tissue samples, using MACS2 narrow (blue, 20 peaks) and broad (green, 27) parameters and LanceOtron (yellow, 908). Also shown are annotation of relevant region, distance of peak from nearest TSS, gene name, gene ID and transcript ID. Peaks detected in multiple callers are shown in the relevant colour for each one.

| ***Chrom*** | ***Start*** | ***End*** | ***Annotation*** | ***Distance to TSS (kb)*** | ***Gene Name*** | ***Gene ID*** | ***TranscriptID*** |
| --- | --- | --- | --- | --- | --- | --- | --- |
| 1 | 567565 | 567614 | Promoter (<=1kb) | -0.523 | RP5-857K21.7 | ENSG00000229344.1 | ENST00000427426.1 |
| 1 | 569908 | 569961 | Promoter (<=1kb) | 0.153 | RP5-857K21.11 | ENSG00000198744.5 | ENST00000416718.2 |
| 1 | 1165119 | 1165235 | Promoter (1-2kb) | -1.144 | SDF4 | ENSG00000078808.12 | ENST00000403997.2 |
| 1 | 8317800 | 8318022 | Distal Intergenic | 17.416 | RN7SL729P | ENSG00000265414.1 | ENST00000585092.1 |
| 1 | 16948281 | 16948498 | Intron (ENST00000540383.1/ENSG00000215908.5, intron 5 of 6) | 4.396 | RP5-1182A14.5 | ENSG00000271732.1 | ENST00000607700.1 |
| 1 | 16952253 | 16952490 | Exon (ENST00000540383.1/ENSG00000215908.5, exon 5 of 7) | 4.911 | CROCCP2 | ENSG00000215908.5 | ENST00000540383.1 |
| 1 | 16954932 | 16955088 | Promoter (2-3kb) | 2.313 | CROCCP2 | ENSG00000215908.5 | ENST00000540383.1 |
| 1 | 46954023 | 46954277 | Distal Intergenic | -18.391 | DMBX1 | ENSG00000197587.6 | ENST00000371956.4 |
| 1 | 91852781 | 91853149 | Intron (ENST00000370425.3/ENSG00000162669.11, intron 4 of 38) | 17.143 | HFM1 | ENSG00000162669.11 | ENST00000455133.1 |
| 1 | 121357993 | 121358048 | Distal Intergenic | -35.672 | RP11-344P13.1 | ENSG00000224857.1 | ENST00000450546.1 |
| 1 | 121483347 | 121483405 | Distal Intergenic | -161.026 | RP11-344P13.1 | ENSG00000224857.1 | ENST00000450546.1 |
| 1 | 121483501 | 121483577 | Distal Intergenic | -161.18 | RP11-344P13.1 | ENSG00000224857.1 | ENST00000450546.1 |
| 1 | 121484126 | 121484568 | Distal Intergenic | -161.805 | RP11-344P13.1 | ENSG00000224857.1 | ENST00000450546.1 |
| 1 | 121484585 | 121485434 | Distal Intergenic | -162.264 | RP11-344P13.1 | ENSG00000224857.1 | ENST00000450546.1 |
| 1 | 142536214 | 142536310 | Distal Intergenic | -16.983 | RP11-417J8.1 | ENSG00000227552.1 | ENST00000445662.1 |
| 1 | 142537329 | 142537503 | Distal Intergenic | -15.79 | RP11-417J8.1 | ENSG00000227552.1 | ENST00000445662.1 |
| 1 | 142537666 | 142537802 | Distal Intergenic | -15.491 | RP11-417J8.1 | ENSG00000227552.1 | ENST00000445662.1 |
| 1 | 142537936 | 142538189 | Distal Intergenic | -15.104 | RP11-417J8.1 | ENSG00000227552.1 | ENST00000445662.1 |
| 1 | 142538260 | 142538353 | Distal Intergenic | -14.94 | RP11-417J8.1 | ENSG00000227552.1 | ENST00000445662.1 |
| 1 | 142538783 | 142538951 | Distal Intergenic | -14.342 | RP11-417J8.1 | ENSG00000227552.1 | ENST00000445662.1 |
| 1 | 143283614 | 143283659 | Distal Intergenic | 37.792 | RP11-782C8.8 | ENSG00000234654.1 | ENST00000445742.1 |
| 1 | 144539479 | 144539583 | Distal Intergenic | 5.442 | RNU1-59P | ENSG00000201699.1 | ENST00000364829.1 |
| 1 | 144539970 | 144540170 | Distal Intergenic | 5.933 | RNU1-59P | ENSG00000201699.1 | ENST00000364829.1 |
| 1 | 145028530 | 145028672 | Intron (ENST00000313382.9/ENSG00000178104.15, intron 1 of 45) | 10.136 | RP11-326G21.1 | ENSG00000272755.1 | ENST00000610119.1 |
| 2 | 33141278 | 33141353 | Intron (ENST00000414054.1/ENSG00000230876.2, intron 4 of 4) | -10.841 | AL121656.5 | ENSG00000236854.1 | ENST00000440786.1 |
| 2 | 33141505 | 33141573 | Intron (ENST00000414054.1/ENSG00000230876.2, intron 4 of 4) | -10.621 | AL121656.5 | ENSG00000236854.1 | ENST00000440786.1 |
| 2 | 33141613 | 33141683 | Intron (ENST00000414054.1/ENSG00000230876.2, intron 4 of 4) | -10.511 | AL121656.5 | ENSG00000236854.1 | ENST00000440786.1 |
| 2 | 87623895 | 87623980 | Distal Intergenic | -16.006 | AC068279.3 | ENSG00000224881.1 | ENST00000444323.1 |
| 2 | 91598212 | 91598218 | Distal Intergenic | -37.713 | AC018696.1 | ENSG00000226615.1 | ENST00000428749.1 |
| 2 | 91599705 | 91599787 | Distal Intergenic | -36.144 | AC018696.1 | ENSG00000226615.1 | ENST00000428749.1 |
| 2 | 91605942 | 91605989 | Distal Intergenic | -29.942 | AC018696.1 | ENSG00000226615.1 | ENST00000428749.1 |
| 2 | 91607487 | 91607619 | Distal Intergenic | -28.312 | AC018696.1 | ENSG00000226615.1 | ENST00000428749.1 |
| 2 | 91610245 | 91610319 | Distal Intergenic | -25.612 | AC018696.1 | ENSG00000226615.1 | ENST00000428749.1 |
| 2 | 91613662 | 91613710 | Distal Intergenic | -22.221 | AC018696.1 | ENSG00000226615.1 | ENST00000428749.1 |
| 2 | 91762248 | 91762629 | Distal Intergenic | -3.908 | AC018696.4 | ENSG00000230964.1 | ENST00000454518.1 |
| 2 | 91813980 | 91813987 | Intron (ENST00000609777.1/ENSG00000143429.5, intron 2 of 2) | 29.417 | AC027612.6 | ENSG00000143429.5 | ENST00000608018.1 |
| 2 | 91814003 | 91814170 | Intron (ENST00000609777.1/ENSG00000143429.5, intron 2 of 2) | 29.234 | AC027612.6 | ENSG00000143429.5 | ENST00000608018.1 |
| 2 | 92269415 | 92269522 | Distal Intergenic | -46.39 | AC128677.4 | ENSG00000223816.4 | ENST00000451163.2 |
| 2 | 92273069 | 92273116 | Distal Intergenic | -50.044 | AC128677.4 | ENSG00000223816.4 | ENST00000451163.2 |
| 2 | 92275917 | 92275980 | Distal Intergenic | -52.892 | AC128677.4 | ENSG00000223816.4 | ENST00000451163.2 |
| 2 | 92281296 | 92281377 | Distal Intergenic | -58.271 | AC128677.4 | ENSG00000223816.4 | ENST00000451163.2 |
| 2 | 92290482 | 92290628 | Distal Intergenic | -67.457 | AC128677.4 | ENSG00000223816.4 | ENST00000451163.2 |
| 2 | 92296716 | 92296772 | Distal Intergenic | -73.691 | AC128677.4 | ENSG00000223816.4 | ENST00000451163.2 |
| 2 | 92296818 | 92296926 | Distal Intergenic | -73.793 | AC128677.4 | ENSG00000223816.4 | ENST00000451163.2 |
| 2 | 92305658 | 92305809 | Distal Intergenic | -82.633 | AC128677.4 | ENSG00000223816.4 | ENST00000451163.2 |
| 2 | 92308043 | 92308092 | Distal Intergenic | -85.018 | AC128677.4 | ENSG00000223816.4 | ENST00000451163.2 |
| 2 | 92317722 | 92317817 | Distal Intergenic | -94.697 | AC128677.4 | ENSG00000223816.4 | ENST00000451163.2 |
| 2 | 92318682 | 92318775 | Distal Intergenic | -95.657 | AC128677.4 | ENSG00000223816.4 | ENST00000451163.2 |
| 2 | 92319109 | 92319170 | Distal Intergenic | -96.084 | AC128677.4 | ENSG00000223816.4 | ENST00000451163.2 |
| 2 | 92321296 | 92321344 | Distal Intergenic | -98.271 | AC128677.4 | ENSG00000223816.4 | ENST00000451163.2 |
| 2 | 92323551 | 92323592 | Distal Intergenic | -100.526 | AC128677.4 | ENSG00000223816.4 | ENST00000451163.2 |
| 2 | 92324182 | 92324267 | Distal Intergenic | -101.157 | AC128677.4 | ENSG00000223816.4 | ENST00000451163.2 |
| 2 | 133011918 | 133012070 | Promoter (1-2kb) | -1.041 | RNA5-8SP5 | ENSG00000202174.1 | ENST00000365304.1 |
| 2 | 133016755 | 133016815 | Promoter (1-2kb) | -1.214 | ANKRD30BL | ENSG00000163046.11 | ENST00000470729.1 |
| 2 | 133016882 | 133017022 | Promoter (1-2kb) | -1.341 | ANKRD30BL | ENSG00000163046.11 | ENST00000470729.1 |
| 2 | 133017159 | 133017366 | Promoter (1-2kb) | -1.618 | ANKRD30BL | ENSG00000163046.11 | ENST00000470729.1 |
| 2 | 133022665 | 133022766 | Promoter (2-3kb) | 2.765 | CDC27P1 | ENSG00000233786.1 | ENST00000445384.1 |
| 2 | 133023080 | 133023288 | Distal Intergenic | 3.18 | CDC27P1 | ENSG00000233786.1 | ENST00000445384.1 |
| 2 | 133023315 | 133023585 | Distal Intergenic | 3.415 | CDC27P1 | ENSG00000233786.1 | ENST00000445384.1 |
| 2 | 133025169 | 133025393 | Distal Intergenic | 5.269 | CDC27P1 | ENSG00000233786.1 | ENST00000445384.1 |
| 2 | 133025612 | 133025963 | Distal Intergenic | 5.712 | CDC27P1 | ENSG00000233786.1 | ENST00000445384.1 |
| 2 | 133029100 | 133029110 | Distal Intergenic | 9.2 | CDC27P1 | ENSG00000233786.1 | ENST00000445384.1 |
| 2 | 133029237 | 133029382 | Distal Intergenic | 9.337 | CDC27P1 | ENSG00000233786.1 | ENST00000445384.1 |
| 2 | 133029588 | 133029716 | Distal Intergenic | 9.688 | CDC27P1 | ENSG00000233786.1 | ENST00000445384.1 |
| 2 | 133029771 | 133029853 | Distal Intergenic | 9.871 | CDC27P1 | ENSG00000233786.1 | ENST00000445384.1 |
| 2 | 162136137 | 162136197 | Promoter (2-3kb) | -2.216 | AC009299.4 | ENSG00000225813.1 | ENST00000429684.1 |
| 3 | 75718337 | 75718481 | Promoter (<=1kb) | 0.256 | RP11-413E6.7 | ENSG00000236138.4 | ENST00000489078.1 |
| 3 | 75718726 | 75718768 | Promoter (<=1kb) | 0.645 | RP11-413E6.7 | ENSG00000236138.4 | ENST00000489078.1 |
| 3 | 75719640 | 75719737 | Promoter (1-2kb) | 1.559 | RP11-413E6.7 | ENSG00000236138.4 | ENST00000489078.1 |
| 4 | 49101116 | 49101147 | Distal Intergenic | 70.408 | CWH43 | ENSG00000109182.7 | ENST00000507372.1 |
| 4 | 49101404 | 49101478 | Distal Intergenic | 70.696 | CWH43 | ENSG00000109182.7 | ENST00000507372.1 |
| 4 | 49102238 | 49102424 | Distal Intergenic | 71.53 | CWH43 | ENSG00000109182.7 | ENST00000507372.1 |
| 4 | 49107270 | 49107465 | Distal Intergenic | 76.562 | CWH43 | ENSG00000109182.7 | ENST00000507372.1 |
| 4 | 49123037 | 49123130 | Distal Intergenic | -76.09 | AC118282.3 | ENSG00000222437.1 | ENST00000410505.1 |
| 4 | 49151213 | 49151252 | Distal Intergenic | -47.968 | AC118282.3 | ENSG00000222437.1 | ENST00000410505.1 |
| 4 | 49151692 | 49151822 | Distal Intergenic | -47.398 | AC118282.3 | ENSG00000222437.1 | ENST00000410505.1 |
| 4 | 49152065 | 49152265 | Distal Intergenic | -46.955 | AC118282.3 | ENSG00000222437.1 | ENST00000410505.1 |
| 4 | 49156498 | 49156537 | Distal Intergenic | -42.683 | AC118282.3 | ENSG00000222437.1 | ENST00000410505.1 |
| 4 | 49512343 | 49512384 | Promoter (1-2kb) | -1.521 | RP11-241F15.3 | ENSG00000250769.2 | ENST00000514077.2 |
| 4 | 49641084 | 49641285 | Distal Intergenic | -39.559 | AC119751.4 | ENSG00000223099.1 | ENST00000411167.1 |
| 4 | 49641439 | 49641471 | Distal Intergenic | -39.914 | AC119751.4 | ENSG00000223099.1 | ENST00000411167.1 |
| 4 | 49645168 | 49645200 | Distal Intergenic | -43.643 | AC119751.4 | ENSG00000223099.1 | ENST00000411167.1 |
| 4 | 49650860 | 49651048 | Distal Intergenic | -49.335 | AC119751.4 | ENSG00000223099.1 | ENST00000411167.1 |
| 4 | 49651151 | 49651340 | Distal Intergenic | -49.626 | AC119751.4 | ENSG00000223099.1 | ENST00000411167.1 |
| 4 | 49659253 | 49659456 | Distal Intergenic | -57.728 | AC119751.4 | ENSG00000223099.1 | ENST00000411167.1 |
| 4 | 49659556 | 49659661 | Distal Intergenic | -58.031 | AC119751.4 | ENSG00000223099.1 | ENST00000411167.1 |
| 4 | 68265540 | 68265771 | Distal Intergenic | 21.001 | RP11-584P21.2 | ENSG00000250075.1 | ENST00000503987.1 |
| 4 | 68265888 | 68265992 | Distal Intergenic | 20.78 | RP11-584P21.2 | ENSG00000250075.1 | ENST00000503987.1 |
| 4 | 68266330 | 68266459 | Distal Intergenic | 20.313 | RP11-584P21.2 | ENSG00000250075.1 | ENST00000503987.1 |
| 4 | 68266574 | 68266648 | Distal Intergenic | 20.124 | RP11-584P21.2 | ENSG00000250075.1 | ENST00000503987.1 |
| 4 | 140097662 | 140097753 | Promoter (<=1kb) | 0.6 | ELF2 | ENSG00000109381.15 | ENST00000511184.1 |
| 4 | 190201023 | 190201272 | Distal Intergenic | 11.218 | RP11-706F1.2 | ENSG00000249877.1 | ENST00000504057.1 |
| 5 | 131339190 | 131339268 | Promoter (<=1kb) | -0.017 | AC034228.3 | ENSG00000223548.1 | ENST00000424244.1 |
| 6 | 58776205 | 58776306 | Distal Intergenic | 287.318 | RP11-143A22.1 | ENSG00000223633.2 | ENST00000457561.2 |
| 6 | 58776501 | 58776627 | Distal Intergenic | 287.614 | RP11-143A22.1 | ENSG00000223633.2 | ENST00000457561.2 |
| 6 | 58776687 | 58776989 | Distal Intergenic | 287.8 | RP11-143A22.1 | ENSG00000223633.2 | ENST00000457561.2 |
| 6 | 58777077 | 58777509 | Distal Intergenic | 288.19 | RP11-143A22.1 | ENSG00000223633.2 | ENST00000457561.2 |
| 6 | 58777573 | 58778080 | Distal Intergenic | 288.686 | RP11-143A22.1 | ENSG00000223633.2 | ENST00000457561.2 |
| 6 | 58778129 | 58778855 | Distal Intergenic | 289.242 | RP11-143A22.1 | ENSG00000223633.2 | ENST00000457561.2 |
| 6 | 58778859 | 58779290 | Distal Intergenic | 289.972 | RP11-143A22.1 | ENSG00000223633.2 | ENST00000457561.2 |
| 7 | 61794583 | 61794635 | Distal Intergenic | -27.234 | RP11-715L17.1 | ENSG00000233918.1 | ENST00000454392.1 |
| 7 | 61967418 | 61967466 | Distal Intergenic | 145.55 | RP11-715L17.1 | ENSG00000233918.1 | ENST00000454392.1 |
| 7 | 61967522 | 61967630 | Distal Intergenic | 145.654 | RP11-715L17.1 | ENSG00000233918.1 | ENST00000454392.1 |
| 7 | 61968939 | 61969084 | Distal Intergenic | 147.071 | RP11-715L17.1 | ENSG00000233918.1 | ENST00000454392.1 |
| 7 | 61969211 | 61969562 | Distal Intergenic | 147.343 | RP11-715L17.1 | ENSG00000233918.1 | ENST00000454392.1 |
| 7 | 61970358 | 61970397 | Distal Intergenic | 148.49 | RP11-715L17.1 | ENSG00000233918.1 | ENST00000454392.1 |
| 7 | 61973049 | 61973215 | Distal Intergenic | 151.181 | RP11-715L17.1 | ENSG00000233918.1 | ENST00000454392.1 |
| 7 | 61975036 | 61975094 | Distal Intergenic | 153.168 | RP11-715L17.1 | ENSG00000233918.1 | ENST00000454392.1 |
| 7 | 61976387 | 61976494 | Distal Intergenic | 154.519 | RP11-715L17.1 | ENSG00000233918.1 | ENST00000454392.1 |
| 7 | 152107755 | 152107994 | Intron (ENST00000262189.6/ENSG00000055609.13, intron 1 of 58) | 7.891 | Y_RNA | ENSG00000253088.1 | ENST00000517279.1 |
| 8 | 43092888 | 43092960 | Distal Intergenic | 9.118 | RP11-726G23.2 | ENSG00000253884.1 | ENST00000518796.1 |
| 8 | 43093078 | 43093162 | Distal Intergenic | 8.916 | RP11-726G23.2 | ENSG00000253884.1 | ENST00000518796.1 |
| 8 | 43093226 | 43093333 | Distal Intergenic | 8.745 | RP11-726G23.2 | ENSG00000253884.1 | ENST00000518796.1 |
| 8 | 43093372 | 43093467 | Distal Intergenic | 8.611 | RP11-726G23.2 | ENSG00000253884.1 | ENST00000518796.1 |
| 8 | 43094677 | 43094722 | Distal Intergenic | 7.356 | RP11-726G23.2 | ENSG00000253884.1 | ENST00000518796.1 |
| 8 | 43094762 | 43094818 | Distal Intergenic | 7.26 | RP11-726G23.2 | ENSG00000253884.1 | ENST00000518796.1 |
| 8 | 43095187 | 43095242 | Distal Intergenic | 6.836 | RP11-726G23.2 | ENSG00000253884.1 | ENST00000518796.1 |
| 8 | 43096883 | 43096924 | Distal Intergenic | 5.154 | RP11-726G23.2 | ENSG00000253884.1 | ENST00000518796.1 |
| 8 | 43794603 | 43794647 | Distal Intergenic | -264.87 | RP11-643N23.1 | ENSG00000253198.1 | ENST00000519417.1 |
| 8 | 43830082 | 43830125 | Distal Intergenic | -300.349 | RP11-643N23.1 | ENSG00000253198.1 | ENST00000519417.1 |
| 8 | 46852458 | 46852515 | Distal Intergenic | -319.667 | AC113134.1 | ENSG00000255915.1 | ENST00000539774.1 |
| 8 | 58118282 | 58118462 | Intron (ENST00000519241.1/ENSG00000253301.1, intron 3 of 3) | 12.104 | RP11-513O17.2 | ENSG00000253301.1 | ENST00000523341.1 |
| 8 | 58118489 | 58118620 | Intron (ENST00000519241.1/ENSG00000253301.1, intron 3 of 3) | -12.215 | RP11-513O17.2 | ENSG00000253301.1 | ENST00000520929.1 |
| 8 | 70602349 | 70602422 | Intron (ENST00000260126.4/ENSG00000137571.6, intron 6 of 9) | -10.037 | RN7SKP29 | ENSG00000222889.1 | ENST00000410957.1 |
| 8 | 70602451 | 70602504 | Intron (ENST00000260126.4/ENSG00000137571.6, intron 6 of 9) | -9.955 | RN7SKP29 | ENSG00000222889.1 | ENST00000410957.1 |
| 9 | 68412475 | 68412813 | Promoter (1-2kb) | 1.383 | LINC00537 | ENSG00000232815.1 | ENST00000455245.1 |
| 9 | 68412877 | 68412884 | Promoter (1-2kb) | 1.312 | LINC00537 | ENSG00000232815.1 | ENST00000455245.1 |
| 9 | 68413791 | 68414160 | Promoter (<=1kb) | 0.036 | LINC00537 | ENSG00000232815.1 | ENST00000455245.1 |
| 9 | 68414445 | 68414525 | Promoter (<=1kb) | -0.25 | LINC00537 | ENSG00000232815.1 | ENST00000455245.1 |
| 9 | 68416017 | 68416130 | Promoter (<=1kb) | 0.71 | MIR4477B | ENSG00000266017.1 | ENST00000581659.1 |
| 9 | 68416166 | 68416238 | Promoter (<=1kb) | 0.859 | MIR4477B | ENSG00000266017.1 | ENST00000581659.1 |
| 9 | 68417178 | 68417425 | Promoter (1-2kb) | 1.871 | MIR4477B | ENSG00000266017.1 | ENST00000581659.1 |
| 9 | 68418793 | 68418892 | Distal Intergenic | 3.486 | MIR4477B | ENSG00000266017.1 | ENST00000581659.1 |
| 9 | 68420217 | 68420352 | Distal Intergenic | 4.91 | MIR4477B | ENSG00000266017.1 | ENST00000581659.1 |
| 9 | 68420466 | 68420605 | Distal Intergenic | 5.159 | MIR4477B | ENSG00000266017.1 | ENST00000581659.1 |
| 9 | 68425694 | 68425827 | Distal Intergenic | 10.387 | MIR4477B | ENSG00000266017.1 | ENST00000581659.1 |
| 9 | 68425838 | 68426050 | Distal Intergenic | 10.531 | MIR4477B | ENSG00000266017.1 | ENST00000581659.1 |
| 9 | 68426146 | 68426292 | Distal Intergenic | 10.839 | MIR4477B | ENSG00000266017.1 | ENST00000581659.1 |
| 9 | 93942669 | 93942883 | Promoter (1-2kb) | -1.456 | RP11-305L7.6 | ENSG00000229694.2 | ENST00000421595.1 |
| 10 | 38817857 | 38818201 | Distal Intergenic | -73.565 | CICP9 | ENSG00000228882.1 | ENST00000444451.1 |
| 10 | 42355325 | 42355415 | Distal Intergenic | 289.582 | KSR1P1 | ENSG00000229485.1 | ENST00000446298.1 |
| 10 | 42355428 | 42355565 | Distal Intergenic | 289.432 | KSR1P1 | ENSG00000229485.1 | ENST00000446298.1 |
| 10 | 42361356 | 42361418 | Distal Intergenic | 283.579 | KSR1P1 | ENSG00000229485.1 | ENST00000446298.1 |
| 10 | 42364734 | 42364794 | Distal Intergenic | 280.203 | KSR1P1 | ENSG00000229485.1 | ENST00000446298.1 |
| 10 | 42365062 | 42365132 | Distal Intergenic | 279.865 | KSR1P1 | ENSG00000229485.1 | ENST00000446298.1 |
| 10 | 42369238 | 42369356 | Distal Intergenic | 275.641 | KSR1P1 | ENSG00000229485.1 | ENST00000446298.1 |
| 10 | 42378054 | 42378319 | Distal Intergenic | 266.678 | KSR1P1 | ENSG00000229485.1 | ENST00000446298.1 |
| 10 | 42379882 | 42379947 | Distal Intergenic | 265.05 | KSR1P1 | ENSG00000229485.1 | ENST00000446298.1 |
| 10 | 42380238 | 42380325 | Distal Intergenic | 264.672 | KSR1P1 | ENSG00000229485.1 | ENST00000446298.1 |
| 10 | 42380747 | 42381058 | Distal Intergenic | 263.939 | KSR1P1 | ENSG00000229485.1 | ENST00000446298.1 |
| 10 | 42381286 | 42381390 | Distal Intergenic | 263.607 | KSR1P1 | ENSG00000229485.1 | ENST00000446298.1 |
| 10 | 42381727 | 42381791 | Distal Intergenic | 263.206 | KSR1P1 | ENSG00000229485.1 | ENST00000446298.1 |
| 10 | 42384241 | 42384380 | Distal Intergenic | 260.617 | KSR1P1 | ENSG00000229485.1 | ENST00000446298.1 |
| 10 | 42384534 | 42384761 | Distal Intergenic | 260.236 | KSR1P1 | ENSG00000229485.1 | ENST00000446298.1 |
| 10 | 42385092 | 42385257 | Distal Intergenic | 259.74 | KSR1P1 | ENSG00000229485.1 | ENST00000446298.1 |
| 10 | 42385513 | 42385585 | Distal Intergenic | 259.412 | KSR1P1 | ENSG00000229485.1 | ENST00000446298.1 |
| 10 | 42387181 | 42387360 | Distal Intergenic | 257.637 | KSR1P1 | ENSG00000229485.1 | ENST00000446298.1 |
| 10 | 42390955 | 42391140 | Distal Intergenic | 253.857 | KSR1P1 | ENSG00000229485.1 | ENST00000446298.1 |
| 10 | 42393377 | 42393479 | Distal Intergenic | 251.518 | KSR1P1 | ENSG00000229485.1 | ENST00000446298.1 |
| 10 | 42393967 | 42394115 | Distal Intergenic | 250.882 | KSR1P1 | ENSG00000229485.1 | ENST00000446298.1 |
| 10 | 42394466 | 42394522 | Distal Intergenic | 250.475 | KSR1P1 | ENSG00000229485.1 | ENST00000446298.1 |
| 10 | 42395185 | 42395235 | Distal Intergenic | 249.762 | KSR1P1 | ENSG00000229485.1 | ENST00000446298.1 |
| 10 | 42395597 | 42395648 | Distal Intergenic | 249.349 | KSR1P1 | ENSG00000229485.1 | ENST00000446298.1 |
| 10 | 42396121 | 42396208 | Distal Intergenic | 248.789 | KSR1P1 | ENSG00000229485.1 | ENST00000446298.1 |
| 10 | 42396432 | 42396491 | Distal Intergenic | 248.506 | KSR1P1 | ENSG00000229485.1 | ENST00000446298.1 |
| 10 | 42396853 | 42396939 | Distal Intergenic | 248.058 | KSR1P1 | ENSG00000229485.1 | ENST00000446298.1 |
| 10 | 42398355 | 42398655 | Distal Intergenic | 246.342 | KSR1P1 | ENSG00000229485.1 | ENST00000446298.1 |
| 10 | 42398686 | 42398738 | Distal Intergenic | 246.259 | KSR1P1 | ENSG00000229485.1 | ENST00000446298.1 |
| 10 | 42399413 | 42399479 | Distal Intergenic | 245.518 | KSR1P1 | ENSG00000229485.1 | ENST00000446298.1 |
| 10 | 42399658 | 42399723 | Distal Intergenic | 245.274 | KSR1P1 | ENSG00000229485.1 | ENST00000446298.1 |
| 10 | 42399872 | 42399977 | Distal Intergenic | 245.02 | KSR1P1 | ENSG00000229485.1 | ENST00000446298.1 |
| 10 | 42401827 | 42401871 | Distal Intergenic | 243.126 | KSR1P1 | ENSG00000229485.1 | ENST00000446298.1 |
| 10 | 42529245 | 42529386 | Distal Intergenic | 115.611 | KSR1P1 | ENSG00000229485.1 | ENST00000446298.1 |
| 10 | 42529567 | 42529621 | Distal Intergenic | 115.376 | KSR1P1 | ENSG00000229485.1 | ENST00000446298.1 |
| 10 | 42529783 | 42530047 | Distal Intergenic | 114.95 | KSR1P1 | ENSG00000229485.1 | ENST00000446298.1 |
| 10 | 42530206 | 42530252 | Distal Intergenic | 114.745 | KSR1P1 | ENSG00000229485.1 | ENST00000446298.1 |
| 10 | 42530617 | 42530671 | Distal Intergenic | 114.326 | KSR1P1 | ENSG00000229485.1 | ENST00000446298.1 |
| 10 | 42531904 | 42531967 | Distal Intergenic | 113.03 | KSR1P1 | ENSG00000229485.1 | ENST00000446298.1 |
| 10 | 42532524 | 42532613 | Distal Intergenic | 112.384 | KSR1P1 | ENSG00000229485.1 | ENST00000446298.1 |
| 10 | 42532695 | 42532828 | Distal Intergenic | 112.169 | KSR1P1 | ENSG00000229485.1 | ENST00000446298.1 |
| 10 | 42534715 | 42534771 | Distal Intergenic | 110.226 | KSR1P1 | ENSG00000229485.1 | ENST00000446298.1 |
| 10 | 42596796 | 42596838 | Distal Intergenic | 48.159 | KSR1P1 | ENSG00000229485.1 | ENST00000446298.1 |
| 10 | 42597133 | 42597216 | Distal Intergenic | 47.781 | KSR1P1 | ENSG00000229485.1 | ENST00000446298.1 |
| 10 | 42599772 | 42599810 | Distal Intergenic | 45.187 | KSR1P1 | ENSG00000229485.1 | ENST00000446298.1 |
| 10 | 42600023 | 42600098 | Distal Intergenic | 44.899 | KSR1P1 | ENSG00000229485.1 | ENST00000446298.1 |
| 10 | 42600153 | 42600192 | Distal Intergenic | 44.805 | KSR1P1 | ENSG00000229485.1 | ENST00000446298.1 |
| 10 | 127584918 | 127585091 | Promoter (<=1kb) | 0 | DHX32 | ENSG00000089876.7 | ENST00000415732.1 |
| 10 | 127585213 | 127585335 | Promoter (<=1kb) | 0.034 | FANK1 | ENSG00000203780.6 | ENST00000449042.2 |
| 10 | 130262825 | 130263022 | Distal Intergenic | -144.348 | RP11-264E18.1 | ENSG00000234640.1 | ENST00000454492.1 |
| 10 | 135500693 | 135500739 | Distal Intergenic | 3.488 | DUX4L15 | ENSG00000235950.4 | ENST00000557077.1 |
| 11 | 51572128 | 51572242 | Distal Intergenic | -44.329 | OR4C50P | ENSG00000237610.1 | ENST00000330155.3 |
| 11 | 51579589 | 51579637 | Distal Intergenic | -51.79 | OR4C50P | ENSG00000237610.1 | ENST00000330155.3 |
| 11 | 51591304 | 51591359 | Distal Intergenic | -63.505 | OR4C50P | ENSG00000237610.1 | ENST00000330155.3 |
| 12 | 34841547 | 34841610 | Distal Intergenic | 439.132 | RP13-7D7.1 | ENSG00000256614.1 | ENST00000540219.1 |
| 12 | 34846311 | 34846356 | Distal Intergenic | 443.896 | RP13-7D7.1 | ENSG00000256614.1 | ENST00000540219.1 |
| 12 | 58026045 | 58026316 | Promoter (<=1kb) | 0.114 | B4GALNT1 | ENSG00000135454.9 | ENST00000418555.2 |
| 16 | 33241062 | 33241183 | Intron (ENST00000398667.4/ENSG00000205457.6, intron 1 of 1) | -5.24 | RP11-1277H1.3 | ENSG00000263337.1 | ENST00000572906.1 |
| 16 | 33294007 | 33294066 | Distal Intergenic | 4.494 | RP11-23E10.5 | ENSG00000262090.1 | ENST00000573021.1 |
| 16 | 33865242 | 33865438 | Distal Intergenic | 47.514 | RP11-598D12.2 | ENSG00000261197.1 | ENST00000566112.1 |
| 16 | 33950256 | 33950370 | Distal Intergenic | -4.236 | AC136932.2 | ENSG00000265616.1 | ENST00000583871.1 |
| 16 | 33950441 | 33950512 | Distal Intergenic | -4.421 | AC136932.2 | ENSG00000265616.1 | ENST00000583871.1 |
| 16 | 33955620 | 33955701 | Distal Intergenic | -5.951 | AC136932.1 | ENSG00000207986.1 | ENST00000385251.1 |
| 16 | 33955765 | 33955924 | Distal Intergenic | -5.728 | AC136932.1 | ENSG00000207986.1 | ENST00000385251.1 |
| 16 | 33961279 | 33961330 | Promoter (<=1kb) | -0.322 | AC136932.1 | ENSG00000207986.1 | ENST00000385251.1 |
| 16 | 33963980 | 33964029 | Promoter (1-2kb) | -1.397 | RNA5-8SP2 | ENSG00000200434.1 | ENST00000363564.1 |
| 16 | 33964031 | 33964153 | Promoter (1-2kb) | -1.273 | RNA5-8SP2 | ENSG00000200434.1 | ENST00000363564.1 |
| 16 | 33964871 | 33964986 | Promoter (<=1kb) | -0.44 | RNA5-8SP2 | ENSG00000200434.1 | ENST00000363564.1 |
| 16 | 33969707 | 33969927 | Distal Intergenic | 4.282 | RNA5-8SP2 | ENSG00000200434.1 | ENST00000363564.1 |
| 16 | 33971136 | 33971300 | Distal Intergenic | 5.711 | RNA5-8SP2 | ENSG00000200434.1 | ENST00000363564.1 |
| 16 | 33972319 | 33972562 | Distal Intergenic | 6.894 | RNA5-8SP2 | ENSG00000200434.1 | ENST00000363564.1 |
| 16 | 33973744 | 33973839 | Distal Intergenic | 8.319 | RNA5-8SP2 | ENSG00000200434.1 | ENST00000363564.1 |
| 16 | 33987861 | 33988052 | Distal Intergenic | 22.436 | RNA5-8SP2 | ENSG00000200434.1 | ENST00000363564.1 |
| 16 | 46385844 | 46385946 | Distal Intergenic | 126.405 | ANKRD26P1 | ENSG00000261239.2 | ENST00000571606.1 |
| 16 | 46385978 | 46386047 | Distal Intergenic | 126.304 | ANKRD26P1 | ENSG00000261239.2 | ENST00000571606.1 |
| 16 | 46386169 | 46386262 | Distal Intergenic | 126.089 | ANKRD26P1 | ENSG00000261239.2 | ENST00000571606.1 |
| 16 | 46386485 | 46386538 | Distal Intergenic | 125.813 | ANKRD26P1 | ENSG00000261239.2 | ENST00000571606.1 |
| 16 | 46386560 | 46386661 | Distal Intergenic | 125.69 | ANKRD26P1 | ENSG00000261239.2 | ENST00000571606.1 |
| 16 | 46386698 | 46386750 | Distal Intergenic | 125.601 | ANKRD26P1 | ENSG00000261239.2 | ENST00000571606.1 |
| 16 | 46388494 | 46388610 | Distal Intergenic | 123.741 | ANKRD26P1 | ENSG00000261239.2 | ENST00000571606.1 |
| 16 | 46389073 | 46389164 | Distal Intergenic | 123.187 | ANKRD26P1 | ENSG00000261239.2 | ENST00000571606.1 |
| 16 | 46390133 | 46390200 | Distal Intergenic | 122.151 | ANKRD26P1 | ENSG00000261239.2 | ENST00000571606.1 |
| 16 | 46390680 | 46390727 | Distal Intergenic | 121.624 | ANKRD26P1 | ENSG00000261239.2 | ENST00000571606.1 |
| 16 | 46390797 | 46390845 | Distal Intergenic | 121.506 | ANKRD26P1 | ENSG00000261239.2 | ENST00000571606.1 |
| 16 | 46391269 | 46391330 | Distal Intergenic | 121.021 | ANKRD26P1 | ENSG00000261239.2 | ENST00000571606.1 |
| 16 | 46391900 | 46392071 | Distal Intergenic | 120.28 | ANKRD26P1 | ENSG00000261239.2 | ENST00000571606.1 |
| 16 | 46393237 | 46393306 | Distal Intergenic | 119.045 | ANKRD26P1 | ENSG00000261239.2 | ENST00000571606.1 |
| 16 | 46393590 | 46393663 | Distal Intergenic | 118.688 | ANKRD26P1 | ENSG00000261239.2 | ENST00000571606.1 |
| 16 | 46394324 | 46394427 | Distal Intergenic | 117.924 | ANKRD26P1 | ENSG00000261239.2 | ENST00000571606.1 |
| 16 | 46394448 | 46394491 | Distal Intergenic | 117.86 | ANKRD26P1 | ENSG00000261239.2 | ENST00000571606.1 |
| 16 | 46395394 | 46395519 | Distal Intergenic | 116.832 | ANKRD26P1 | ENSG00000261239.2 | ENST00000571606.1 |
| 16 | 46395530 | 46395590 | Distal Intergenic | 116.761 | ANKRD26P1 | ENSG00000261239.2 | ENST00000571606.1 |
| 16 | 46396686 | 46396766 | Distal Intergenic | 115.585 | ANKRD26P1 | ENSG00000261239.2 | ENST00000571606.1 |
| 16 | 46401324 | 46401387 | Distal Intergenic | 110.964 | ANKRD26P1 | ENSG00000261239.2 | ENST00000571606.1 |
| 16 | 46403378 | 46403436 | Distal Intergenic | 108.915 | ANKRD26P1 | ENSG00000261239.2 | ENST00000571606.1 |
| 16 | 46403657 | 46403746 | Distal Intergenic | 108.605 | ANKRD26P1 | ENSG00000261239.2 | ENST00000571606.1 |
| 16 | 46404945 | 46404997 | Distal Intergenic | 107.354 | ANKRD26P1 | ENSG00000261239.2 | ENST00000571606.1 |
| 16 | 46405131 | 46405181 | Distal Intergenic | 107.17 | ANKRD26P1 | ENSG00000261239.2 | ENST00000571606.1 |
| 16 | 46405879 | 46405917 | Distal Intergenic | 106.434 | ANKRD26P1 | ENSG00000261239.2 | ENST00000571606.1 |
| 16 | 46406948 | 46406991 | Distal Intergenic | 105.36 | ANKRD26P1 | ENSG00000261239.2 | ENST00000571606.1 |
| 16 | 46407683 | 46407750 | Distal Intergenic | 104.601 | ANKRD26P1 | ENSG00000261239.2 | ENST00000571606.1 |
| 16 | 46417035 | 46417286 | Distal Intergenic | 95.065 | ANKRD26P1 | ENSG00000261239.2 | ENST00000571606.1 |
| 16 | 46425423 | 46425467 | Distal Intergenic | 86.884 | ANKRD26P1 | ENSG00000261239.2 | ENST00000571606.1 |
| 16 | 46426288 | 46426357 | Distal Intergenic | 85.994 | ANKRD26P1 | ENSG00000261239.2 | ENST00000571606.1 |
| 16 | 46426943 | 46427126 | Distal Intergenic | 85.225 | ANKRD26P1 | ENSG00000261239.2 | ENST00000571606.1 |
| 16 | 46427470 | 46427605 | Distal Intergenic | 84.746 | ANKRD26P1 | ENSG00000261239.2 | ENST00000571606.1 |
| 16 | 46427610 | 46427671 | Distal Intergenic | 84.68 | ANKRD26P1 | ENSG00000261239.2 | ENST00000571606.1 |
| 16 | 46427924 | 46427977 | Distal Intergenic | 84.374 | ANKRD26P1 | ENSG00000261239.2 | ENST00000571606.1 |
| 16 | 46429690 | 46429757 | Distal Intergenic | 82.594 | ANKRD26P1 | ENSG00000261239.2 | ENST00000571606.1 |
| 16 | 46432723 | 46432774 | Distal Intergenic | 79.577 | ANKRD26P1 | ENSG00000261239.2 | ENST00000571606.1 |
| 16 | 46435483 | 46435545 | Distal Intergenic | 76.806 | ANKRD26P1 | ENSG00000261239.2 | ENST00000571606.1 |
| 16 | 67251168 | 67251228 | Intron (ENST00000409509.1/ENSG00000125122.10, intron 2 of 5) | -6.181 | LRRC29 | ENSG00000125122.10 | ENST00000409037.1 |
| 16 | 68397070 | 68397402 | Promoter (<=1kb) | 0.194 | SMPD3 | ENSG00000103056.7 | ENST00000563396.1 |
| 17 | 7324665 | 7324769 | Promoter (<=1kb) | 0.987 | SPEM1 | ENSG00000181323.7 | ENST00000323675.3 |
| 17 | 21209025 | 21209276 | Intron (ENST00000395491.2/ENSG00000034152.14, intron 10 of 12) | -5.723 | MAP2K3 | ENSG00000034152.14 | ENST00000477540.1 |
| 17 | 21906279 | 21906332 | Promoter (<=1kb) | -0.085 | RP11-744K17.9 | ENSG00000266795.2 | ENST00000581223.2 |
| 17 | 21906340 | 21906430 | Promoter (<=1kb) | 0 | RP11-744K17.9 | ENSG00000266795.2 | ENST00000581223.2 |
| 17 | 22020698 | 22020748 | Promoter (1-2kb) | -1.689 | MTRNR2L1 | ENSG00000256618.1 | ENST00000540040.1 |
| 18 | 107604 | 107650 | Promoter (1-2kb) | -1.415 | ROCK1P1 | ENSG00000263006.2 | ENST00000608049.1 |
| 18 | 108191 | 108328 | Promoter (<=1kb) | -0.737 | ROCK1P1 | ENSG00000263006.2 | ENST00000608049.1 |
| 18 | 108366 | 108479 | Promoter (<=1kb) | -0.586 | ROCK1P1 | ENSG00000263006.2 | ENST00000608049.1 |
| 18 | 108699 | 108756 | Promoter (<=1kb) | -0.309 | ROCK1P1 | ENSG00000263006.2 | ENST00000608049.1 |
| 18 | 108774 | 108931 | Promoter (<=1kb) | -0.134 | ROCK1P1 | ENSG00000263006.2 | ENST00000608049.1 |
| 18 | 109064 | 109204 | Promoter (<=1kb) | 0 | ROCK1P1 | ENSG00000263006.2 | ENST00000608049.1 |
| 18 | 109259 | 109606 | Promoter (<=1kb) | 0.195 | ROCK1P1 | ENSG00000263006.2 | ENST00000608049.1 |
| 18 | 18511657 | 18511703 | Distal Intergenic | 23.128 | ROCK1 | ENSG00000067900.6 | ENST00000578051.1 |
| 18 | 18512052 | 18512206 | Distal Intergenic | 22.625 | ROCK1 | ENSG00000067900.6 | ENST00000578051.1 |
| 18 | 18512255 | 18512377 | Distal Intergenic | 22.454 | ROCK1 | ENSG00000067900.6 | ENST00000578051.1 |
| 18 | 18512946 | 18513140 | Distal Intergenic | 21.691 | ROCK1 | ENSG00000067900.6 | ENST00000578051.1 |
| 18 | 18513181 | 18513313 | Distal Intergenic | 21.518 | ROCK1 | ENSG00000067900.6 | ENST00000578051.1 |
| 18 | 18514973 | 18515062 | Distal Intergenic | 19.769 | ROCK1 | ENSG00000067900.6 | ENST00000578051.1 |
| 18 | 18516129 | 18516194 | Distal Intergenic | 18.637 | ROCK1 | ENSG00000067900.6 | ENST00000578051.1 |
| 18 | 18516836 | 18517200 | Distal Intergenic | 17.631 | ROCK1 | ENSG00000067900.6 | ENST00000578051.1 |
| 18 | 18518095 | 18518177 | Distal Intergenic | 16.654 | ROCK1 | ENSG00000067900.6 | ENST00000578051.1 |
| 18 | 18518712 | 18519101 | Distal Intergenic | 15.73 | ROCK1 | ENSG00000067900.6 | ENST00000578051.1 |
| 18 | 18519140 | 18519227 | Distal Intergenic | 15.604 | ROCK1 | ENSG00000067900.6 | ENST00000578051.1 |
| 18 | 18519803 | 18520327 | Distal Intergenic | 14.504 | ROCK1 | ENSG00000067900.6 | ENST00000578051.1 |
| 18 | 69340918 | 69341203 | Distal Intergenic | 8.535 | RP11-752P2.2 | ENSG00000265946.1 | ENST00000582687.1 |
| 19 | 7515378 | 7515397 | Intron (ENST00000593531.1/ENSG00000268861.1, intron 8 of 21) | 3.43 | ARHGEF18 | ENSG00000104880.13 | ENST00000594665.1 |
| 19 | 27731999 | 27732277 | Distal Intergenic | 399.269 | CTB-151G24.1 | ENSG00000267696.2 | ENST00000598435.1 |
| 19 | 27732284 | 27732340 | Distal Intergenic | 399.206 | CTB-151G24.1 | ENSG00000267696.2 | ENST00000598435.1 |
| 19 | 27732694 | 27732855 | Distal Intergenic | 398.691 | CTB-151G24.1 | ENSG00000267696.2 | ENST00000598435.1 |
| 19 | 27732977 | 27733126 | Distal Intergenic | 398.42 | CTB-151G24.1 | ENSG00000267696.2 | ENST00000598435.1 |
| 19 | 27733183 | 27733438 | Distal Intergenic | 398.108 | CTB-151G24.1 | ENSG00000267696.2 | ENST00000598435.1 |
| 19 | 27733853 | 27733907 | Distal Intergenic | 397.639 | CTB-151G24.1 | ENSG00000267696.2 | ENST00000598435.1 |
| 19 | 27734227 | 27734274 | Distal Intergenic | 397.272 | CTB-151G24.1 | ENSG00000267696.2 | ENST00000598435.1 |
| 19 | 27734611 | 27734797 | Distal Intergenic | 396.749 | CTB-151G24.1 | ENSG00000267696.2 | ENST00000598435.1 |
| 19 | 27735318 | 27735516 | Distal Intergenic | 396.03 | CTB-151G24.1 | ENSG00000267696.2 | ENST00000598435.1 |
| 19 | 27735908 | 27735975 | Distal Intergenic | 395.571 | CTB-151G24.1 | ENSG00000267696.2 | ENST00000598435.1 |
| 19 | 27736445 | 27736588 | Distal Intergenic | 394.958 | CTB-151G24.1 | ENSG00000267696.2 | ENST00000598435.1 |
| 19 | 27736710 | 27736805 | Distal Intergenic | 394.741 | CTB-151G24.1 | ENSG00000267696.2 | ENST00000598435.1 |
| 19 | 27737436 | 27737542 | Distal Intergenic | 394.004 | CTB-151G24.1 | ENSG00000267696.2 | ENST00000598435.1 |
| 19 | 27738381 | 27738804 | Distal Intergenic | 392.742 | CTB-151G24.1 | ENSG00000267696.2 | ENST00000598435.1 |
| 19 | 27739194 | 27739211 | Distal Intergenic | 392.335 | CTB-151G24.1 | ENSG00000267696.2 | ENST00000598435.1 |
| 19 | 27739278 | 27739372 | Distal Intergenic | 392.174 | CTB-151G24.1 | ENSG00000267696.2 | ENST00000598435.1 |
| 19 | 27740192 | 27740379 | Distal Intergenic | 391.167 | CTB-151G24.1 | ENSG00000267696.2 | ENST00000598435.1 |
| 19 | 54641179 | 54641608 | Promoter (<=1kb) | 0 | CNOT3 | ENSG00000088038.13 | ENST00000221232.5 |
| 20 | 26318461 | 26318565 | Distal Intergenic | -86.3 | MIR663A | ENSG00000227195.4 | ENST00000601119.1 |
| 20 | 29582860 | 29582972 | Intron (ENST00000432067.1/ENSG00000231934.1, intron 3 of 3) | -12.498 | RP4-610C12.1 | ENSG00000231934.1 | ENST00000445151.1 |
| 20 | 29593170 | 29593266 | Intron (ENST00000432067.1/ENSG00000231934.1, intron 1 of 3) | 3.14 | RP4-610C12.1 | ENSG00000231934.1 | ENST00000432067.1 |
| 20 | 29618975 | 29619119 | Intron (ENST00000278882.3/ENSG00000149531.10, intron 2 of 8) | 6.715 | FRG1B | ENSG00000149531.10 | ENST00000482423.1 |
| 20 | 29646736 | 29646931 | Distal Intergenic | -8.677 | MLLT10P1 | ENSG00000238151.1 | ENST00000418346.1 |
| 20 | 47132160 | 47132251 | Distal Intergenic | -9.606 | RNU7-144P | ENSG00000238452.1 | ENST00000459405.1 |
| 21 | 9825536 | 9825676 | Promoter (<=1kb) | -0.156 | MIR3648 | ENSG00000264462.1 | ENST00000581792.1 |
| 21 | 9825755 | 9825952 | Promoter (<=1kb) | 0 | MIR3648 | ENSG00000264462.1 | ENST00000581792.1 |
| 21 | 9825976 | 9826033 | Promoter (<=1kb) | 0.145 | MIR3648 | ENSG00000264462.1 | ENST00000581792.1 |
| 21 | 9826043 | 9826114 | Promoter (<=1kb) | -0.089 | MIR3687 | ENSG00000264063.1 | ENST00000577708.1 |
| 21 | 9826173 | 9826262 | Promoter (<=1kb) | 0 | MIR3687 | ENSG00000264063.1 | ENST00000577708.1 |
| 21 | 9826323 | 9826505 | Promoter (<=1kb) | 0.121 | MIR3687 | ENSG00000264063.1 | ENST00000577708.1 |
| 21 | 9826522 | 9826623 | Promoter (<=1kb) | 0.32 | MIR3687 | ENSG00000264063.1 | ENST00000577708.1 |
| 21 | 9826659 | 9826910 | Promoter (<=1kb) | 0.457 | MIR3687 | ENSG00000264063.1 | ENST00000577708.1 |
| 21 | 9827016 | 9827082 | Promoter (<=1kb) | 0.814 | MIR3687 | ENSG00000264063.1 | ENST00000577708.1 |
| 21 | 9827133 | 9827218 | Promoter (<=1kb) | 0.931 | MIR3687 | ENSG00000264063.1 | ENST00000577708.1 |
| 21 | 9827318 | 9827362 | Promoter (1-2kb) | 1.116 | MIR3687 | ENSG00000264063.1 | ENST00000577708.1 |
| 21 | 9827469 | 9827517 | Promoter (1-2kb) | 1.267 | MIR3687 | ENSG00000264063.1 | ENST00000577708.1 |
| 21 | 10699745 | 10699892 | Distal Intergenic | -162.73 | IGHV1OR21-1 | ENSG00000169861.8 | ENST00000302092.5 |
| 21 | 10701130 | 10701786 | Distal Intergenic | -160.836 | IGHV1OR21-1 | ENSG00000169861.8 | ENST00000302092.5 |
| 21 | 10702041 | 10702269 | Distal Intergenic | -160.353 | IGHV1OR21-1 | ENSG00000169861.8 | ENST00000302092.5 |
| 21 | 10702432 | 10702567 | Distal Intergenic | -160.055 | IGHV1OR21-1 | ENSG00000169861.8 | ENST00000302092.5 |
| 21 | 10702581 | 10702833 | Distal Intergenic | -159.789 | IGHV1OR21-1 | ENSG00000169861.8 | ENST00000302092.5 |
| 21 | 10702883 | 10702929 | Distal Intergenic | -159.693 | IGHV1OR21-1 | ENSG00000169861.8 | ENST00000302092.5 |
| 21 | 10704876 | 10705019 | Distal Intergenic | -157.603 | IGHV1OR21-1 | ENSG00000169861.8 | ENST00000302092.5 |
| 21 | 10716067 | 10716193 | Distal Intergenic | -146.429 | IGHV1OR21-1 | ENSG00000169861.8 | ENST00000302092.5 |
| 21 | 10716235 | 10716379 | Distal Intergenic | -146.243 | IGHV1OR21-1 | ENSG00000169861.8 | ENST00000302092.5 |
| 21 | 10719959 | 10720160 | Distal Intergenic | -142.462 | IGHV1OR21-1 | ENSG00000169861.8 | ENST00000302092.5 |
| 21 | 10721066 | 10721357 | Distal Intergenic | -141.265 | IGHV1OR21-1 | ENSG00000169861.8 | ENST00000302092.5 |
| 21 | 10721728 | 10721880 | Distal Intergenic | -140.742 | IGHV1OR21-1 | ENSG00000169861.8 | ENST00000302092.5 |
| 21 | 10721932 | 10722092 | Distal Intergenic | -140.53 | IGHV1OR21-1 | ENSG00000169861.8 | ENST00000302092.5 |
| 21 | 10773403 | 10773486 | Distal Intergenic | -89.136 | IGHV1OR21-1 | ENSG00000169861.8 | ENST00000302092.5 |
| 21 | 10773931 | 10774194 | Distal Intergenic | -88.428 | IGHV1OR21-1 | ENSG00000169861.8 | ENST00000302092.5 |
| 21 | 11056700 | 11056889 | Promoter (1-2kb) | 1.434 | BAGE2 | ENSG00000187172.11 | ENST00000496773.1 |
| 21 | 11059891 | 11060052 | Promoter (1-2kb) | -1.569 | BAGE2 | ENSG00000187172.11 | ENST00000496773.1 |
| 21 | 11112499 | 11112624 | Distal Intergenic | -13.52 | BAGE2 | ENSG00000187172.11 | ENST00000474011.1 |
| 21 | 11114919 | 11115136 | Distal Intergenic | -15.94 | BAGE2 | ENSG00000187172.11 | ENST00000474011.1 |
| 21 | 11147732 | 11147923 | Distal Intergenic | 7.134 | VN1R7P | ENSG00000231962.1 | ENST00000414133.1 |
| 21 | 11186184 | 11186289 | Distal Intergenic | -4.37 | EIF3FP1 | ENSG00000234643.1 | ENST00000414503.1 |
| 21 | 11186576 | 11186675 | Distal Intergenic | -4.762 | EIF3FP1 | ENSG00000234643.1 | ENST00000414503.1 |
| 22 | 35623017 | 35623305 | Intron (ENST00000423311.1/ENSG00000233080.2, intron 1 of 5) | 3.744 | CTA-714B7.5 | ENSG00000233080.2 | ENST00000423311.1 |
| X | 61733813 | 61734055 | Distal Intergenic | -264.665 | RP11-3D23.1 | ENSG00000236852.1 | ENST00000455793.1 |
| Y | 9929967 | 9930129 | Promoter (<=1kb) | 0.473 | RNA5SP519 | ENSG00000252289.1 | ENST00000516480.1 |
| Y | 9984607 | 9985174 | Distal Intergenic | 22.749 | AC006987.6 | ENSG00000226975.1 | ENST00000413610.1 |
| Y | 10012660 | 10012812 | Promoter (1-2kb) | 1.199 | PCMTD1P1 | ENSG00000226061.1 | ENST00000455560.1 |
| Y | 10019359 | 10019405 | Distal Intergenic | 7.898 | PCMTD1P1 | ENSG00000226061.1 | ENST00000455560.1 |
| Y | 10021829 | 10021890 | Distal Intergenic | 8.017 | CDC27P2 | ENSG00000237447.1 | ENST00000425026.1 |
| Y | 10022443 | 10022498 | Distal Intergenic | 7.409 | CDC27P2 | ENSG00000237447.1 | ENST00000425026.1 |
| Y | 10023013 | 10023060 | Distal Intergenic | 6.847 | CDC27P2 | ENSG00000237447.1 | ENST00000425026.1 |
| Y | 10023188 | 10023254 | Distal Intergenic | 6.653 | CDC27P2 | ENSG00000237447.1 | ENST00000425026.1 |
| Y | 13447348 | 13447477 | Distal Intergenic | -15.117 | DUX4L16 | ENSG00000258567.1 | ENST00000555130.1 |
| Y | 13448271 | 13448320 | Distal Intergenic | -14.274 | DUX4L16 | ENSG00000258567.1 | ENST00000555130.1 |
| Y | 13451216 | 13451292 | Distal Intergenic | -11.302 | DUX4L16 | ENSG00000258567.1 | ENST00000555130.1 |
| Y | 13451303 | 13451468 | Distal Intergenic | -11.126 | DUX4L16 | ENSG00000258567.1 | ENST00000555130.1 |
| Y | 13453598 | 13453771 | Distal Intergenic | -8.823 | DUX4L16 | ENSG00000258567.1 | ENST00000555130.1 |
| Y | 13460420 | 13460472 | Promoter (2-3kb) | -2.122 | DUX4L16 | ENSG00000258567.1 | ENST00000555130.1 |
| Y | 13460987 | 13461165 | Promoter (1-2kb) | -1.429 | DUX4L16 | ENSG00000258567.1 | ENST00000555130.1 |
| Y | 13461205 | 13461389 | Promoter (1-2kb) | -1.205 | DUX4L16 | ENSG00000258567.1 | ENST00000555130.1 |
| Y | 13462128 | 13462246 | Promoter (<=1kb) | -0.348 | DUX4L16 | ENSG00000258567.1 | ENST00000555130.1 |
| Y | 13462350 | 13462394 | Promoter (<=1kb) | -0.2 | DUX4L16 | ENSG00000258567.1 | ENST00000555130.1 |
| Y | 13463258 | 13463316 | Promoter (<=1kb) | 0.665 | DUX4L16 | ENSG00000258567.1 | ENST00000555130.1 |
| Y | 13465916 | 13465969 | Distal Intergenic | 3.323 | DUX4L16 | ENSG00000258567.1 | ENST00000555130.1 |
| Y | 13466021 | 13466077 | Distal Intergenic | 3.428 | DUX4L16 | ENSG00000258567.1 | ENST00000555130.1 |
| Y | 13466505 | 13466548 | Distal Intergenic | 3.912 | DUX4L16 | ENSG00000258567.1 | ENST00000555130.1 |
| Y | 13466747 | 13466941 | Distal Intergenic | -3.656 | DUX4L17 | ENSG00000259154.1 | ENST00000557360.1 |
| Y | 13467322 | 13467459 | Distal Intergenic | -3.138 | DUX4L17 | ENSG00000259154.1 | ENST00000557360.1 |
| Y | 13467561 | 13467591 | Distal Intergenic | -3.006 | DUX4L17 | ENSG00000259154.1 | ENST00000557360.1 |
| Y | 13467775 | 13467879 | Promoter (2-3kb) | -2.718 | DUX4L17 | ENSG00000259154.1 | ENST00000557360.1 |
| Y | 13468072 | 13468134 | Promoter (2-3kb) | -2.463 | DUX4L17 | ENSG00000259154.1 | ENST00000557360.1 |
| Y | 13468461 | 13468622 | Promoter (1-2kb) | -1.975 | DUX4L17 | ENSG00000259154.1 | ENST00000557360.1 |
| Y | 13468953 | 13469021 | Promoter (1-2kb) | -1.576 | DUX4L17 | ENSG00000259154.1 | ENST00000557360.1 |
| Y | 13469276 | 13469443 | Promoter (1-2kb) | -1.154 | DUX4L17 | ENSG00000259154.1 | ENST00000557360.1 |
| Y | 13470172 | 13470220 | Promoter (<=1kb) | -0.377 | DUX4L17 | ENSG00000259154.1 | ENST00000557360.1 |
| Y | 13470451 | 13470645 | Promoter (<=1kb) | 0 | DUX4L17 | ENSG00000259154.1 | ENST00000557360.1 |
| Y | 13473346 | 13473506 | Promoter (2-3kb) | 2.75 | DUX4L17 | ENSG00000259154.1 | ENST00000557360.1 |
| Y | 13476556 | 13476740 | Promoter (<=1kb) | -0.493 | DUX4L18 | ENSG00000259029.1 | ENST00000553347.1 |
| Y | 13478220 | 13478339 | Promoter (<=1kb) | 0.988 | DUX4L18 | ENSG00000259029.1 | ENST00000553347.1 |
| Y | 13479327 | 13479435 | Promoter (2-3kb) | 2.095 | DUX4L18 | ENSG00000259029.1 | ENST00000553347.1 |
| Y | 13479533 | 13479653 | Promoter (2-3kb) | 2.301 | DUX4L18 | ENSG00000259029.1 | ENST00000553347.1 |
| Y | 13479943 | 13480229 | Promoter (2-3kb) | 2.711 | DUX4L18 | ENSG00000259029.1 | ENST00000553347.1 |
| Y | 13480346 | 13480458 | Distal Intergenic | 3.114 | DUX4L18 | ENSG00000259029.1 | ENST00000553347.1 |
| Y | 13481470 | 13481605 | Distal Intergenic | 4.238 | DUX4L18 | ENSG00000259029.1 | ENST00000553347.1 |
| Y | 13481658 | 13481664 | Distal Intergenic | 4.426 | DUX4L18 | ENSG00000259029.1 | ENST00000553347.1 |
| Y | 13482122 | 13482277 | Distal Intergenic | 4.89 | DUX4L18 | ENSG00000259029.1 | ENST00000553347.1 |
| Y | 13482309 | 13482416 | Distal Intergenic | 5.077 | DUX4L18 | ENSG00000259029.1 | ENST00000553347.1 |
| Y | 13482448 | 13482490 | Distal Intergenic | 5.216 | DUX4L18 | ENSG00000259029.1 | ENST00000553347.1 |
| Y | 13484732 | 13484844 | Distal Intergenic | -3.161 | DUX4L19 | ENSG00000258991.1 | ENST00000557448.1 |
| Y | 13485231 | 13485395 | Promoter (2-3kb) | -2.61 | DUX4L19 | ENSG00000258991.1 | ENST00000557448.1 |
| Y | 13485407 | 13485456 | Promoter (2-3kb) | -2.549 | DUX4L19 | ENSG00000258991.1 | ENST00000557448.1 |
| Y | 13485584 | 13485695 | Promoter (2-3kb) | -2.31 | DUX4L19 | ENSG00000258991.1 | ENST00000557448.1 |
| Y | 13485854 | 13485924 | Promoter (2-3kb) | -2.081 | DUX4L19 | ENSG00000258991.1 | ENST00000557448.1 |
| Y | 13485960 | 13486229 | Promoter (1-2kb) | -1.776 | DUX4L19 | ENSG00000258991.1 | ENST00000557448.1 |
| Y | 13487683 | 13487888 | Promoter (<=1kb) | -0.117 | DUX4L19 | ENSG00000258991.1 | ENST00000557448.1 |
| Y | 13488856 | 13489195 | Promoter (<=1kb) | 0.852 | DUX4L19 | ENSG00000258991.1 | ENST00000557448.1 |
| Y | 13489566 | 13489745 | Promoter (1-2kb) | -1.558 | PABPC1P5 | ENSG00000270455.1 | ENST00000603738.1 |
| Y | 13489769 | 13489864 | Promoter (1-2kb) | -1.439 | PABPC1P5 | ENSG00000270455.1 | ENST00000603738.1 |
| Y | 13489911 | 13489965 | Promoter (1-2kb) | -1.338 | PABPC1P5 | ENSG00000270455.1 | ENST00000603738.1 |
| Y | 13490018 | 13490076 | Promoter (1-2kb) | -1.227 | PABPC1P5 | ENSG00000270455.1 | ENST00000603738.1 |
| Y | 28817294 | 28817356 | Distal Intergenic | -36.496 | PARP4P1 | ENSG00000237917.1 | ENST00000435945.1 |
| Y | 58856054 | 58856100 | Distal Intergenic | -145.291 | CTBP2P1 | ENSG00000235857.1 | ENST00000431853.1 |
| Y | 58862694 | 58862754 | Distal Intergenic | -138.637 | CTBP2P1 | ENSG00000235857.1 | ENST00000431853.1 |
| Y | 58975787 | 58975873 | Distal Intergenic | -25.518 | CTBP2P1 | ENSG00000235857.1 | ENST00000431853.1 |
| Y | 58977756 | 58977891 | Distal Intergenic | -23.5 | CTBP2P1 | ENSG00000235857.1 | ENST00000431853.1 |
| Y | 58979009 | 58979061 | Distal Intergenic | -22.33 | CTBP2P1 | ENSG00000235857.1 | ENST00000431853.1 |
| Y | 58979378 | 58979452 | Distal Intergenic | -21.939 | CTBP2P1 | ENSG00000235857.1 | ENST00000431853.1 |
| Y | 58980654 | 58980767 | Distal Intergenic | -20.624 | CTBP2P1 | ENSG00000235857.1 | ENST00000431853.1 |
| Y | 58984999 | 58985071 | Distal Intergenic | -16.32 | CTBP2P1 | ENSG00000235857.1 | ENST00000431853.1 |
| Y | 58985173 | 58985250 | Distal Intergenic | -16.141 | CTBP2P1 | ENSG00000235857.1 | ENST00000431853.1 |
| Y | 58985551 | 58985669 | Distal Intergenic | -15.722 | CTBP2P1 | ENSG00000235857.1 | ENST00000431853.1 |
| Y | 58996627 | 58996761 | Distal Intergenic | -4.63 | CTBP2P1 | ENSG00000235857.1 | ENST00000431853.1 |
| Y | 59004799 | 59005050 | Distal Intergenic | 3.409 | CTBP2P1 | ENSG00000235857.1 | ENST00000431853.1 |

**Supplementary Table S5. Sites of IGF-1R enrichment coincident with ARBSs in clinical prostate cancers.** Table shows 407 IGF-1R peaks identified here in clinical prostate cancers by ChIP-seq that overlap with ARBSs detected in 2 cases of treatment naïve prostate cancer tissue by (Sharma et al., 2013). Table shows chromosome coordinates for each peak, annotation of relevant region, distance of peak from nearest TSS, gene name, gene ID and transcript ID.

| ***Sample/Gene-set*** | ***Total genes with TSS proximal to peak*** |
| --- | --- |
| RP2_1 | 54 |
| RP2_2 | 36 |
| RP3 | 55 |
| RP4 | 102 |
| RP5 | 1760 |
| RP6 | 43 |
|  |  |
| Union gene-set | 1875 |
| Consensus gene-set (genes in 2 or more samples) | 95 |
| Consensus genes in 2 samples only | 62 |
| Consensus genes in 3 samples only | 24 |
| Consensus genes in 4 samples only | 4 |
| Consensus genes in 5 samples only | 4 |
| Consensus genes in 6 samples only | 1 |

**Supplementary Table S6: Numbers of genes with proximal peak of IGF-1R recruitment.** Table shows numbers of genes per individual tissue sample, union gene set of sites proximal to the TSS of any gene in any sample, and ‘consensus’ genes with a TSS proximal to sites of IGF-1R recruitment detected by any peak caller in more than one tissue.

| ***Tissues with IGF-1R peak proximal to TSS (n)*** | ***Gene name*** | ***Function*** |
| --- | --- | --- |
| 4 | ANKRD30BL (Ankyrin Repeat Domain 30B Like) | Protein coding gene |
|  | RP11-241F15.3 | Psuedogene |
|  | CTD-3224I3.3 | Non-coding RNA gene |
|  | DDX11L8 (DEAD/H-Box Helicase 11 Like 8) | Pseudogene - Predicted to enable DNA helicase activity. Predicted to be involved in DNA duplex unwinding and establishment of sister chromatid cohesion. |
| 5 | DDX11L1 (DEAD/H-Box Helicase 11 Like 1) | Pseudogene |
|  | DDX11L16 (DEAD/H-Box Helicase 11 Like 16) | Pseudogene |
|  | DUX4L17 (Double Homeobox 4 Like 17) | Pseudogene |
|  | MIR3687 (MicroRNA 3687-2) | Non-coding RNA gene |
| 6 | ROCK1P1 (Rho Associated Coiled-Coil Containing Protein Kinase 1 Pseudogene 1) | Pseudogene |

Supplementary Table S7: Genes containing IGF-1R peak proximal to their TSS in 4 or more samples. Table shows number of samples that contained an IGF-1R peak within gene TSS, gene name and basic description of gene function. From GeneCards: The Human Gene Database (https://www.genecards.org).

| ***ORA*** | ***Gene-sets in which significant*** | ***ID*** | ***Description*** | ***Gene ratio*** | ***p-value*** |
| --- | --- | --- | --- | --- | --- |
| GO | Union | GO:0090630 | Activation of GTPase activity | 27/118 | 1.75E-07 |
| KEGG | Consensus | hsa03008 | Ribosome biogenesis in eukaryotes | 5/105 | 1.34E-06 |
|  |  | hsa03010 | Ribosome | 5/105 | 8.59E-06 |

**Supplementary Table S8: Pathways significantly over-represented in union and consensus gene-sets.** GO enrichment analysis did not identify significantly over-represented pathways in the Consensus gene set, and KEGG enrichment analysis did not identify significantly over-represented pathways in the union gene set.

| *Motif* | *E-value* | *Matches per sequence* | *Similar known motifs* |
| --- | --- | --- | --- |
| 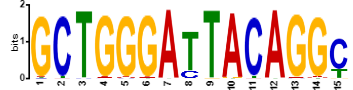 | 1.75e-032 | 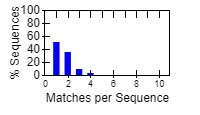 | PITX2 |
| 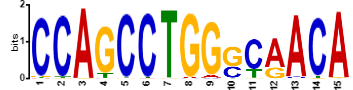 | 1.01e-028 | 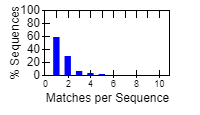 | ZN121 |
| 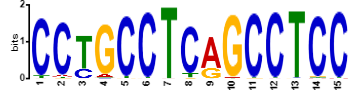 | 8.27e-026 | 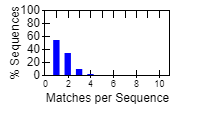 | ZN770 |
| 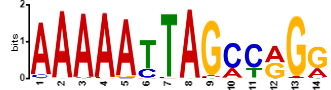 | 3.00e-027 | 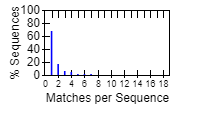 | MEF2D |
| 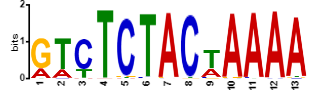 | 2.22e-022 | 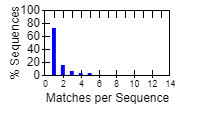 |  |
| 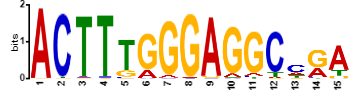 | 1.44e-005 | 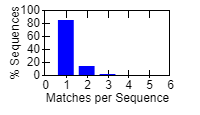 | ZN770, IKZF1 |
| 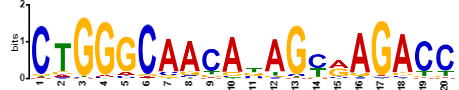 | 6.51e-172 | 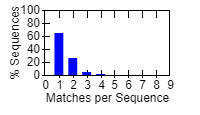 | ZN121 |
| 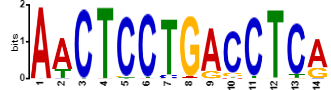 | 4.66e-017 | 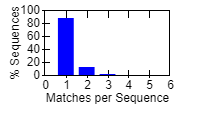 | RXRB |
| 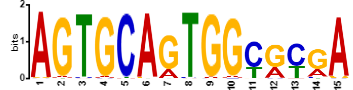 | 4.46e-021 | 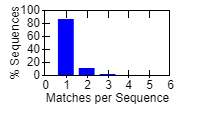 |  |
| 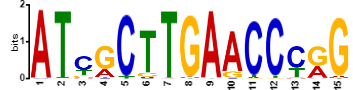 | 3.87e-019 | 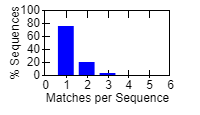 | VDR |
| 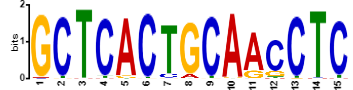 | 4.98e-022 | 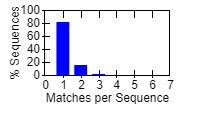 |  |
| 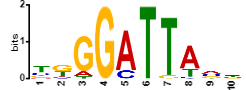 | 1.35e-129 | 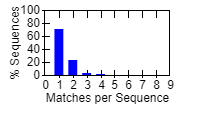 | PITX2 |
| 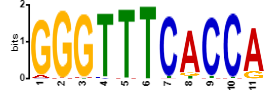 | 3.92e-016 | 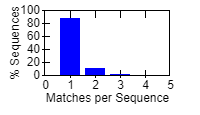 | ZEP1 |
| 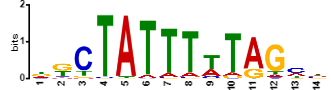 | 2.87e-092 | 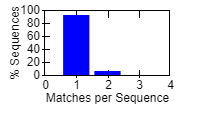 |  |
| 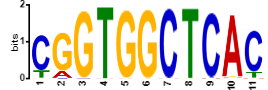 | 6.09e-012 | 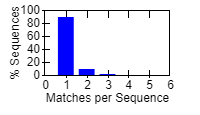 | JUND |
| 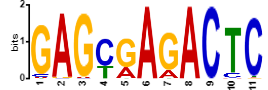 | 1.27e-010 | 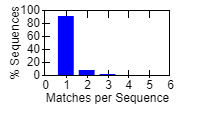 | ZN121 |
| 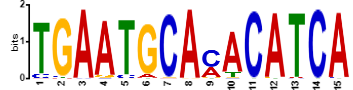 | 4.71e-010 | 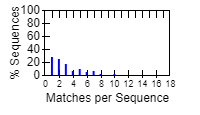 | ZN582, PO5F1, FOXH1 |
| 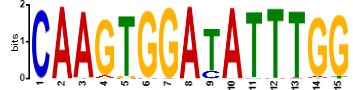 | 1.23e-007 | 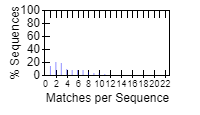 |  |
| 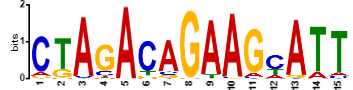 | 3.78e-002 | 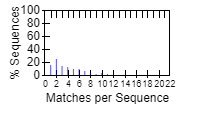 | SMCA1 |
| 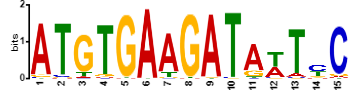 | 1.71e-002 | 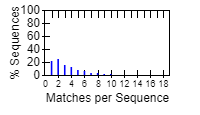 |  |
| 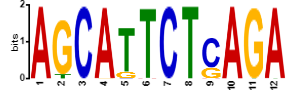 | 8.10e-010 | 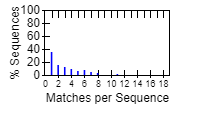 |  |
| 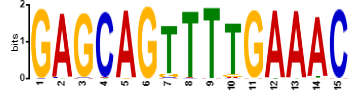 | 2.94e-008 | 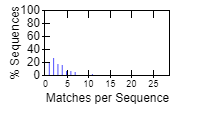 | PRDM4 |
| 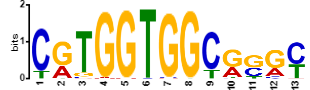 | 4.97e-011 | 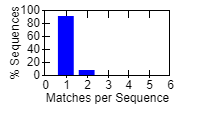 | ARNT2, ZNF41 |
| 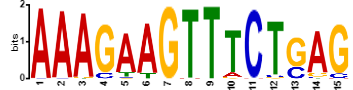 | 3.63e-005 | 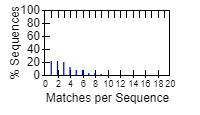 |  |
| 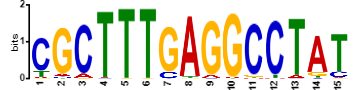 | 9.54e-007 | 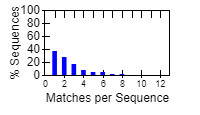 |  |
| 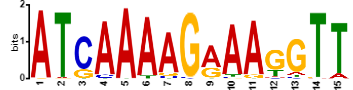 | 1.44e-005 | 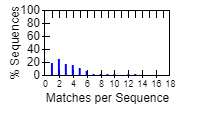 |  |
| 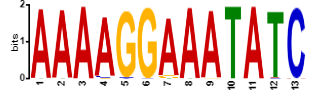 | 2.94e-008 | 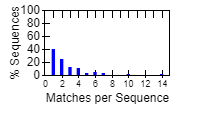 | ELF3 |
| 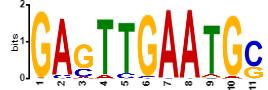 | 1.67e-003 | 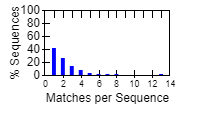 | SMCA5, ZN502, ZN582 |
| 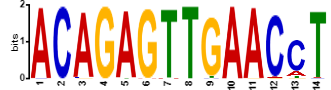 | 3.72e-006 | 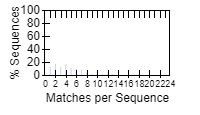 |  |
| 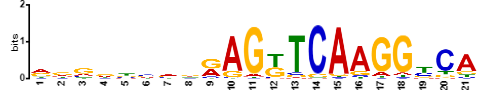 | 2.14e-061 | 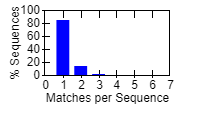 |  |
| 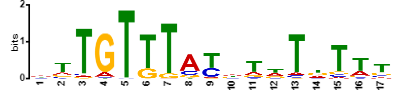 | 2.44e-050 | 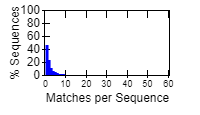 | FOXG1 |
| 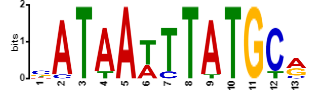 | 5.49e-047 | 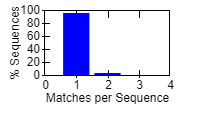 | PO6F1 |
| 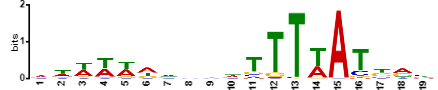 | 4.09e-046 | 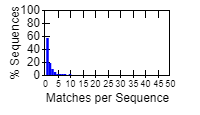 | HXC10 |
| 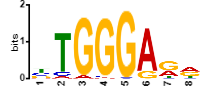 | 1.52e-041 | 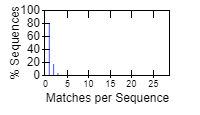 | IKZF1 |
| 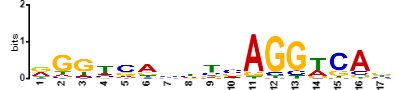 | 1.04e-039 | 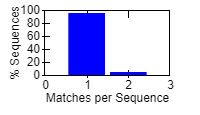 | THA |
| 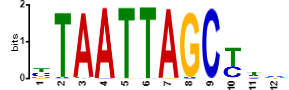 | 2.46e-038 | 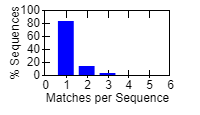 | VSX2 |
| 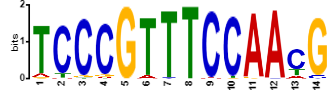 | 1.06e-002 | 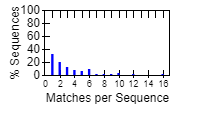 | CENPB |
| 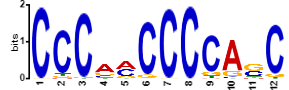 | 1.23e-002 | 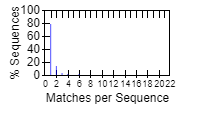 | ZN281 |
| 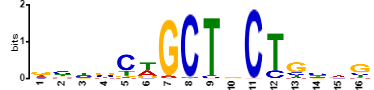 | 1.02e-019 | 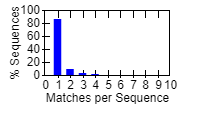 | OSR2 |
| 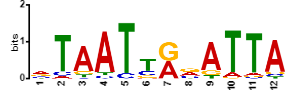 | 1.54e-019 | 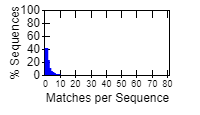 | ALX1 |
| 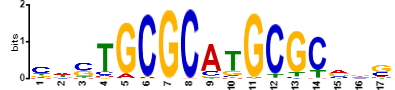 | 6.66e-017 | 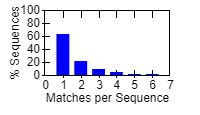 | NRF1 |
| 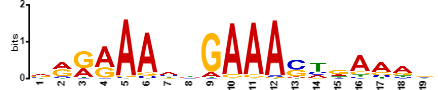 | 1.42e-012 | 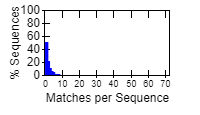 | STAT1 |
| 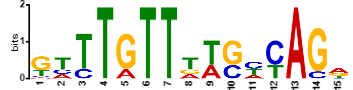 | 3.74e-012 | 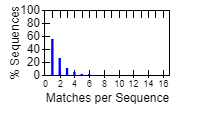 | FOXC2 |
| 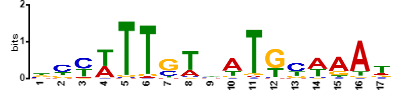 | 3.37e-011 | 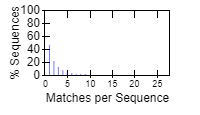 |  |
| 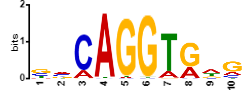 | 4.03e-011 | 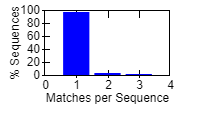 | ZEB1 |
| 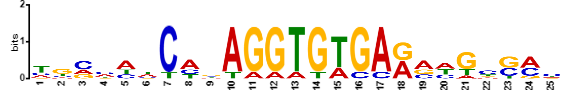 | 7.33e-011 | 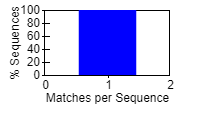 | TBX2 |
| 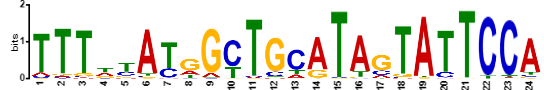 | 1.22e-010 | 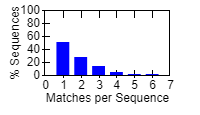 | ZN260 |
| 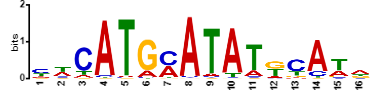 | 1.05e-009 | 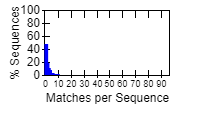 |  |
| 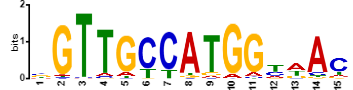 | 3.99e-009 |  | RFX4 |
|  | 3.26e-008 |  | ZF64A |
|  | 9.76e-008 |  | STA5A |
|  | 9.84e-007 |  |  |
|  | 1.18e-006 |  | FOXB1 |
|  | 2.81e-005 |  | ZNF85 |
|  | 3.98e-005 |  | CEBPB |
|  | 4.57e-005 |  |  |
|  | 4.61e-005 |  | KLF14 |
|  | 5.53e-005 |  | ATF2 |
|  | 7.75e-005 |  | ZN554 |
|  | 1.93e-003 |  | ZN436 |
|  | 2.01e-003 |  | ZBTB6 |
|  | 2.80e-003 |  | TYY1 |
|  | 6.22e-003 |  | SMAD1 |
|  | 7.23e-003 |  | NR1D1 |
|  | 7.59e-003 |  | ERG |
|  | 1.01e-002 |  |  |
|  | 4.21e-002 |  | P73 |
|  | 4.42e-002 |  | IRX3 |

Supplementary Table S9: IGF-1R DNA binding motifs identified using MEME-suite. Table showing significantly enriched *de novo* motifs identified using the MEME-suite XSTREME algorithm of the 5743 unique IGF-1R binding sites identified in RP biopsies. *E*-values for each motif are shown along with the number of occurrences of the motif within each individual peak sequence and the percentage of peak sequences that the motif was identified in. Known or similar motifs from the human Hocomoco v11 database identified by the Tomtom algorithm are also shown.

# *References*

1. Singh AA, Schuurman K, Nevedomskaya E, Stelloo S, Linder S, Droog M, Kim Y, Sanders J, Van Der Poel H, Bergman AM, Wessels LFA, Zwart W (2019) Optimized ChIP-seq method facilitates transcription factor profiling in human tumors. *Life Science Alliance*; **2**(1):1-12.

2. Aleksic T, Gray N, Wu X, Rieunier G, Osher E, Mills J, Verrill C, Bryant RJ, Han C, Hutchinson K, Lambert AG, Kumar R, Hamdy FC, Weyer-Czernilofsky U, Sanderson MP, Bogenrieder T, Taylor S, Macaulay VM (2018) Nuclear IGF1R Interacts with Regulatory Regions of Chromatin to Promote RNA Polymerase II Recruitment and Gene Expression Associated with Advanced Tumor Stage. *Cancer Res*; **78**(13):3497-509.

3. Zhang Y, Liu T, Meyer CA, Eeckhoute J, Johnson DS, Bernstein BE, Nussbaum C, Myers RM, Brown M, Li W, Shirley XS (2008) Model-based analysis of ChIP-Seq (MACS). *Genome Biology*; **9**(9).

4. Hentges LD, Sergeant MJ, Cole CB, Downes DJ, Hughes JR, Taylor S (2022) LanceOtron: a deep learning peak caller for genome sequencing experiments. *Bioinformatics*.

5. Mills JV, Osher E, Rieunier G, Mills IG, Macaulay VM (2021) IGF-1R nuclear import and recruitment to chromatin involves both alpha and beta subunits. *Discov Oncol*; **12**(1):13.

6. Yan J, Chen S-AA, Local A, Liu T, Qiu Y, Dorighi KM, Preissl S, Rivera CM, Wang C, Ye Z, Ge K, Hu M, Wysocka J, Ren B (2018) Histone H3 lysine 4 monomethylation modulates long-range chromatin interactions at enhancers. *Cell Research*; **28**(2):204-20.

7. Local A, Huang H, Albuquerque CP, Singh N, Lee AY, Wang W, Wang C, Hsia JE, Shiau AK, Ge K, Corbett KD, Wang D, Zhou H, Ren B (2018) Identification of H3K4me1-associated proteins at mammalian enhancers. *Nat Genet*; **50**(1):73-82.

8. Pundhir S, Bagger FO, Lauridsen FB, Rapin N, Porse BT (2016) Peak-valley-peak pattern of histone modifications delineates active regulatory elements and their directionality. *Nucleic Acids Res*; **44**(9):4037-51.

9. Yashar WM, Kong G, VanCampen J, Curtiss BM, Coleman DJ, Carbone L, Yardimci GG, Maxson JE, Braun TP (2022) GoPeaks: histone modification peak calling for CUT&Tag. *Genome Biology*; **23**(1):144.

10. Bae S, Lesch BJ (2020) H3K4me1 Distribution Predicts Transcription State and Poising at Promoters. *Front Cell Dev Biol*; **8**:289.

11. Landt SG, Marinov GK, Kundaje A, Kheradpour P, Pauli F, Batzoglou S, Bernstein BE, Bickel P, Brown JB, Cayting P, Chen Y, DeSalvo G, Epstein C, Fisher-Aylor KI, Euskirchen G, Gerstein M, Gertz J, Hartemink AJ, Hoffman MM, Iyer VR, Jung YL, Karmakar S, Kellis M, Kharchenko PV, Li Q, Liu T, Liu XS, Ma L, Milosavljevic A, Myers RM, Park PJ, Pazin MJ, Perry MD, Raha D, Reddy TE, Rozowsky J, Shoresh N, Sidow A, Slattery M, Stamatoyannopoulos JA, Tolstorukov MY, White KP, Xi S, Farnham PJ, Lieb JD, Wold BJ, Snyder M (2012) ChIP-seq guidelines and practices of the ENCODE and modENCODE consortia. *Genome Res*; **22**(9):1813-31.

12. Mendoza-Parra MA, Van Gool W, Mohamed Saleem MA, Ceschin DG, Gronemeyer H (2013) A quality control system for profiles obtained by ChIP sequencing. *Nucleic Acids Res*; **41**(21):e196.

13. Nakato R, Shirahige K (2016) Recent advances in ChIP-seq analysis: from quality management to whole-genome annotation. *Briefings in Bioinformatics*; **18**(2):279-90.

14. Jung YL, Luquette LJ, Ho JW, Ferrari F, Tolstorukov M, Minoda A, Issner R, Epstein CB, Karpen GH, Kuroda MI, Park PJ (2014) Impact of sequencing depth in ChIP-seq experiments. *Nucleic Acids Res*; **42**(9):e74.

15. Bailey T, Krajewski P, Ladunga I, Lefebvre C, Li Q, Liu T, Madrigal P, Taslim C, Zhang J (2013) Practical guidelines for the comprehensive analysis of ChIP-seq data. *PLoS Comput Biol*; **9**(11):e1003326.

16. Nakato R, Sakata T (2021) Methods for ChIP-seq analysis: A practical workflow and advanced applications. *Methods*; **187**:44-53.

17. Gates LA, Foulds CE, O'Malley BW (2017) Histone Marks in the 'Driver's Seat': Functional Roles in Steering the Transcription Cycle. *Trends Biochem Sci*; **42**(12):977-89.

18. Mundade R, Ozer HG, Wei H, Prabhu L, Lu T (2014) Role of ChIP-seq in the discovery of transcription factor binding sites, differential gene regulation mechanism, epigenetic marks and beyond. *Cell Cycle*; **13**(18):2847-52.

19. Calo E, Wysocka J (2013) Modification of Enhancer Chromatin: What, How, and Why? *Molecular Cell*; **49**(5):825-37.

20. (2012) An integrated encyclopedia of DNA elements in the human genome. *Nature*; **489**(7414):57-74.

21. Janssen JAMJL (2019) IGF-I and the endocrinology of aging. *Current Opinion in Endocrine and Metabolic Research*; **5**:1-6.

22. Mukama T, Srour B, Johnson T, Katzke V, Kaaks R (2023) IGF-1 and Risk of Morbidity and Mortality From Cancer, Cardiovascular Diseases, and All Causes in EPIC-Heidelberg. *The Journal of Clinical Endocrinology & Metabolism*; **108**(10):e1092-e105.

23. Langmead B, Salzberg SL (2012) Fast gapped-read alignment with Bowtie 2. *Nature Methods*; **9**(4):357-9.

24. Quinlan AR, Hall IM (2010) BEDTools: a flexible suite of utilities for comparing genomic features. *Bioinformatics*; **26**(6):841-2.
